# Helical π‑Conjugated Structure Based on Complementary Helical Monomers with Circularly Polarized Luminescence

**DOI:** 10.1021/acs.orglett.5c02299

**Published:** 2025-07-10

**Authors:** Rafael G. Uceda, Ana M. Ortuño, Luis Álvarez de Cienfuegos, Federico Movilla, Antonio J. Mota, Delia Miguel, Juan M. Cuerva

**Affiliations:** † Department of Organic Chemistry, Faculty of Sciences, Unidad de Excelencia de Química (UEQ), University of Granada, Avda. Fuente Nueva s/n, 18071 Granada, Spain; ‡ Department of Physical Chemistry, Faculty of Pharmacy, (UEQ) University of Granada, C. U. Cartuja, 18071 Granada, Spain; § Department of Inorganic Chemistry, Faculty of Sciences, Unidad de Excelencia de Química (UEQ), University of Granada, Avda. Fuente Nueva s/n, 18071 Granada, Spain

## Abstract

A fully π-conjugated helical system combining rigid [6]­helicenes and a flexible *o*-OPE unit has been developed, yielding enantiopure emitters with up to three helical loops. The compounds display remarkable chiroptical properties in absorption (*g*_abs_ up to 2.3 × 10^–2^) and emission (*g*_lum_ up to 1.3 × 10^–2^), especially in MeOH. A combined DFT and MD approach reveals the conformational dynamics behind the optical behavior, offering a robust methodology for understanding flexible CPL-active systems.

## Graphical abstract

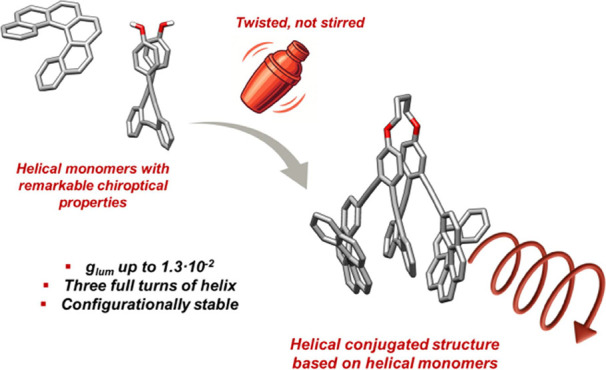

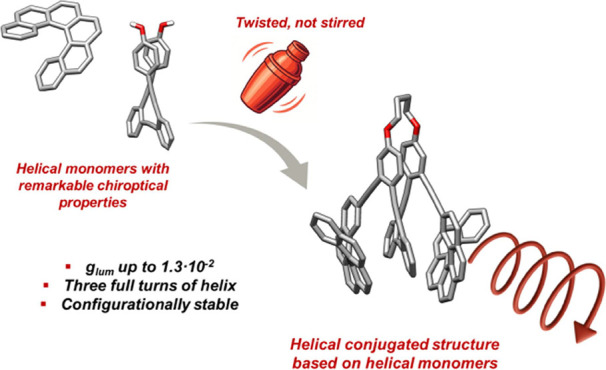

Helical architectures are ubiquitous in nature and central to the function of living systems, inspiring chemists to design synthetic analogues with enhanced properties beyond those of natural monomers.^1–5^ In this context, non-natural foldamers capable of adopting helical (*P*/*M*) conformations have been developed.^6^ In the absence of chiral influences, these systems are racemic, but persistent chiral stimuli can yield enantiopure, shape-persistent helices with stable properties.^7^ Among their features, chiroptical properties are particularly attractive for applications in advanced sensing and anticounterfeiting technologies.^8–12^ Left and right circularly polarized photons can be differently absorbed (Circular Dichroism, CD) or emitted (Circularly Polarized Luminescence, CPL) by chiral matter, creating a new information channel. Nevertheless, for practical applications emitted photons are more interesting than absorbed ones. Therefore, molecules able to produce an unbalanced number of circularly polarized photons are highly appealing.^13–16^ The degree of polarization is quantified by the luminescence dissymmetry factor *g*_lum_ = 2­(I_L_ – I_R_)/(I_L_ + I_R_), which ranges from ±2 for purely circular emissions to 0 for unpolarized light, offering a direct measure of CPL efficiency.^17,18^

Within this context, we have reported that fully conjugated helical structures are especially appealing in terms of chiroptical properties (Figure 1). Thus, for example [n]­helicenes and related structures present intense responses in CD in some S_0_ to S_n_ transitions.^19–22^ We could demonstrate that the reason is based on the instant solenoid behavior of the system during the photon absorption.^23^ Consequently, helical structures encircling higher inner areas should result in enhanced chiroptical properties. It is also worth noting that for CPL the only important transition is the radiative deactivation of the S_1_ excited state to the S_0_ ground state. Although [n]­helicenes give weak CPL transitions,^20,24^ we developed a family of π-conjugated architectures based on *ortho-*oligophenylethynylene (*o*-OPE),^25,26^ which present extraordinary chiroptical responses with *g*_lum_ values in the range of 1 to 5.5 × 10^–2^ due to a larger inner area and the alkyne-mediated delocalization of the S_1_→ S_0_ transition.^27–30^ Some attempts have been made to elongate the structure up to four and a half loops^31^ but carbophilic metals are required to avoid the undesired intrinsic disorder of the system. At this point we wondered if a combination of configurationally rigid [n]­helicenes and the interesting CPL properties of *o*-OPEs could be combined to create a shape-persistent CPL emitter. The combination of chirality and fully π-conjugated structures is also appealing in the context of generating spin polarized currents by the Chiral Induced Spin Selectivity (CISS) effect^32–35^ and the relationship between enhanced chiroptical properties and efficient CISS behavior has been proposed by us^31^ and others.^36–38^

**Figure 1:**
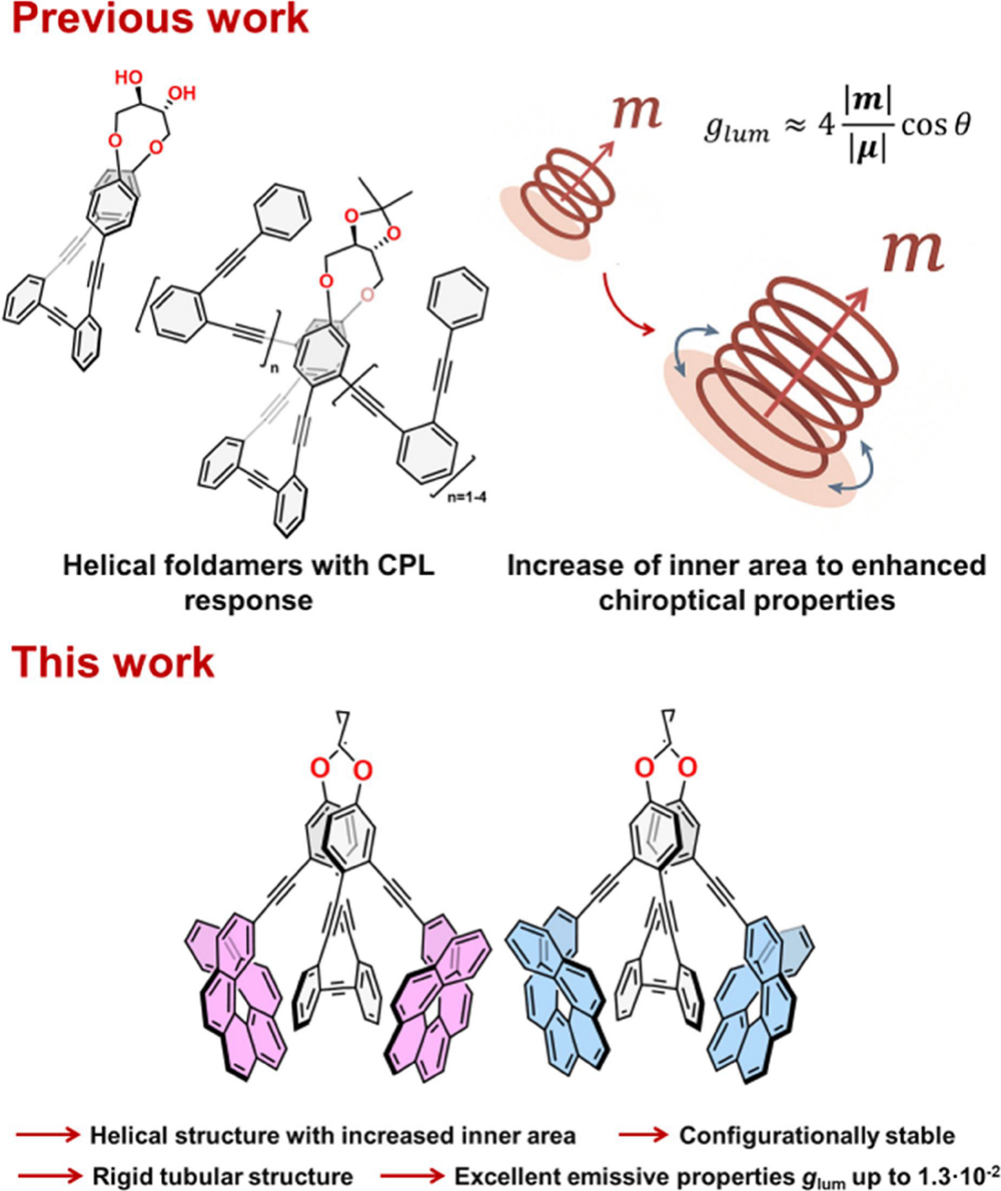
Previous work and working hypothesis.

In this work we have prepared the enantiomeric pair (*P,P,P*)/(*M,M,M*)-**1** using a new strategy consisting in the synergy of two complementary helical monomers. In this way, helicene-like structures can be prepared overpassing the length of parent [n]­helicene, with two and a half loops at the maximum.^39^ Despite its conceptual simplicity, the design is challenging: [n]­helicene-based polymers often fail to form true helicoidal backbones due to geometric mismatch with the linkers, resulting in a helicene containing polymer and not a helical polymer.^40–44^ The simplest solution developed here is the arrangement of configurationally stable [6]­helicenes with a conformationally labile *o*-OPE-based helical monomer. In this case, the global helical chirality would be controlled by the helicene subunits owing to the flexibility of the stapled *o*-OPE core. That is, it can be reaccommodated to both *P*/*M* helicities, depending on its surroundings. In that way, we have created a fully conjugated and folded system with up to three loops without the aid of any metal.^31^ As expected, the dissymmetric factors of the absorption (S_0_ to S_1_) and emission (S_1_ to S_0_) are in the range of 10^–2^. The synthetic route started with the preparation of macrocyclic dibromo precursor **3** using a dialkylation reaction with the corresponding ditosylate **4** of known diphenol **5** ^30^ (Scheme 1). Synthesis of compound **1** was carried out via Sonogashira coupling of stapled *o*-OPE **3** and known racemic alkynyl[6]­helicene **2**.^44^

**Scheme 1:**
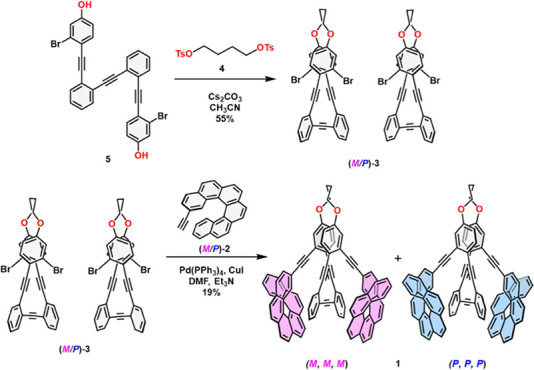
Synthesis of (*P,P,P*)-**1** and (*M,M,M*)-**1**

Subsequent purification by semipreparative chiral HPLC (Supporting Information (SI)) yielded three peaks. The first two correspond to the enantiomeric pair (*P,P,P*)/(*M,M,M*)-**1**, each composed by homochiral (*P*)- or (*M*)- [6]­helicenes coupled by a flexible (*P*)- or (*M*)- *o*-OPE subunit. Although diastereomeric conformations such as (*P*,*M*,*P*)**-1** are theoretically accessible, DFT calculations (Figure 2 and SI) indicate that they are energetically disfavored and therefore negligibly populated. The global minimum corresponds to the fully folded homochiral (*P,P,P*)/(*M,M,M*)-**1** conformer, which accounts for >99% of the ensemble under solvent continuum conditions. This predominance, together with the simplicity of the ^1^H and ^13^C NMR spectra, suggests an effective chirality transfer leading to a single dominant stereoisomer. The remaining HPLC peak contains the (*P*,*P*,*M*)/(*M*,*M*,*P*)-**1** pair, but these diastereomers rapidly racemize at room temperature due to the flexible *o*-OPE core, with a barrier of 1.1 kcal·mol^–1^ at ωB97X-D/6-31G­(d,p) level of theory, preventing their use in chiral emission studies.

**Figure 2:**
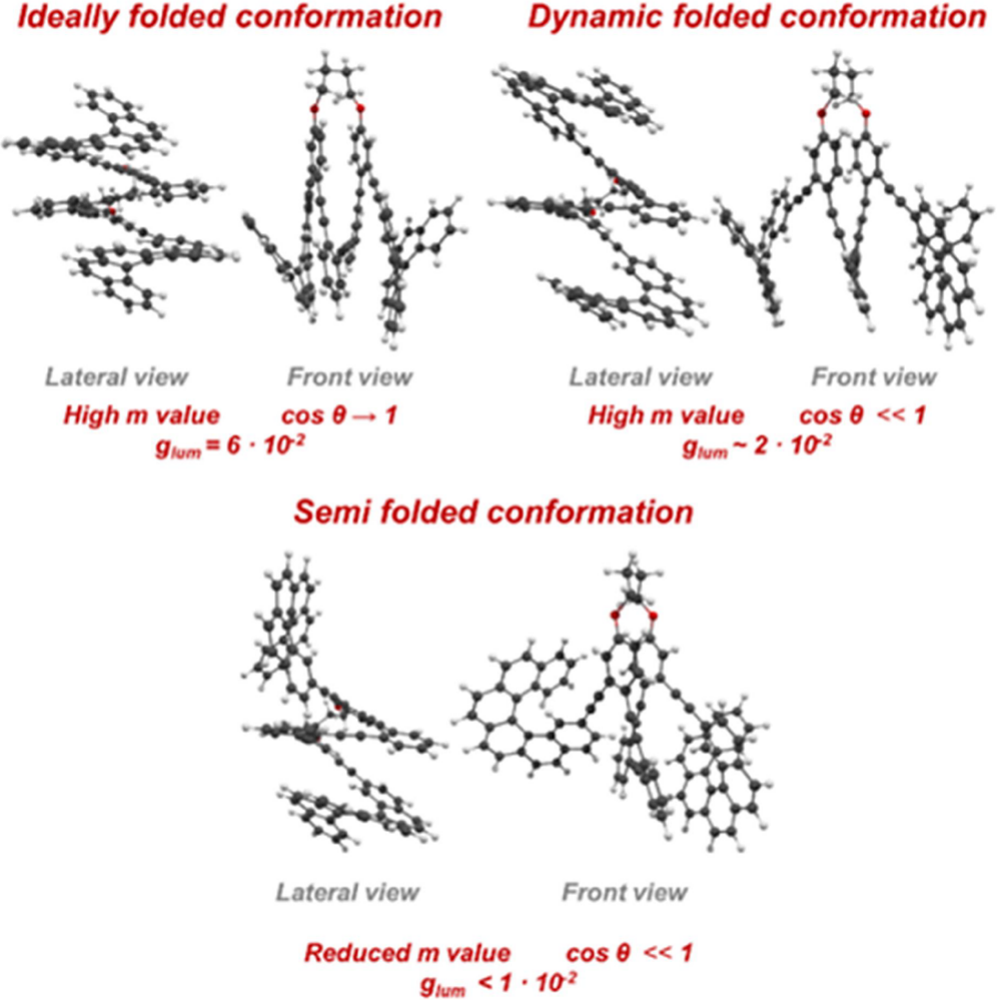
Lateral and front views of main conformers obtained by DFT with their respective *g*_*lum*_ and cos θ values.

We then performed the photophysical properties of the compounds. In this sense, absorbance spectrum of 2.5 × 10^–5^ M solutions in CH_2_Cl_2_ shows a main band centered at 275 nm with a shoulder from 330 to 400 nm, and excitation at 300 nm yields emission peaks at ca. 420 and 445 nm. These features essentially unaltered across solvents of differing polarity (hexane, acetonitrile, methanol; Figure S5). Fluorescence quantum yields (Table S3) vary from 1.8% (hexane) to 4.7% (acetonitrile), and time-resolved decays fit a biexponential model with a short component of ∼2 ns and a long component ranging from 8.0 ns (hexane) to 10.5 ns (CH_2_Cl_2_).

Next, the chiroptical properties of these enantiopure (*P,P,P*)/(*M,M,M*)-**1** were investigated in the four solvents, obtaining in all the cases mirror images for both enantiomers as expected. For the case of (*P,P,P*)-**1**, ECD spectra had similar profile in all the solvents with the same alternance of bands with positive and negative Cotton effects, but with significant differences in intensity among solvents (see Figure S6). The strongest signals were observed in methanol (Figure 3a), with positive Cotton effects at 345, 357, and 382 nm (maximum Δε = 82 M^–1^ cm^–1^ at 357 nm), and a negative band at 296 nm (Δε = −21.3 M^–1^ cm^–1^).

**Figure 3:**
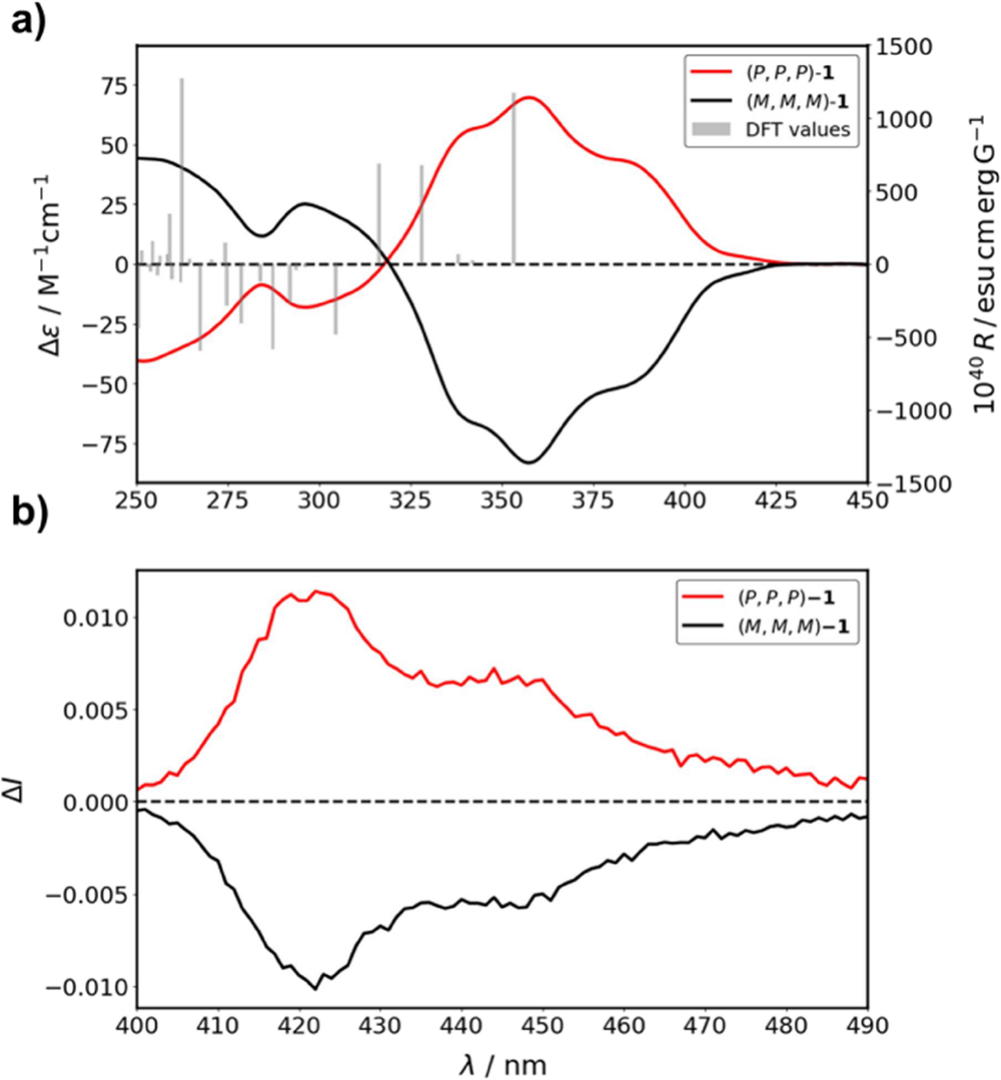
a) Experimental ECD and calculated rotatory strengths (bars outlined in gray) and b) CPL spectra of (*P,P,P*)-**1** (red lines) and (*M,M,M*)-**1** (black lines) in MeOH.

For isotropic samples, chiroptical properties in terms of dissymmetric factors depends on the following equation, *g* ≈ 4|*m*| |μ| cos θ/(|μ|^2^ + |*m*|^2^), being |*m*| and |μ| the modules of the magnetic and electric dipole transition moments and θ the angle between the corresponding vectors. This equation applies for all S_0_→S_n_ transitions for CD and S_1_→S_0_ for CPL. All those parameters can be DFT-calculated, being very informative about the origin of the response of the system to polarized light. When the system is dynamic the experimental signal depends on the Boltzmann weighted signal using the main conformers. In the present case, DFT calculations show that the structure is fully folded (Figure 2, top part), and the different conformers present in the mixture differ in the conformation of the carbon-based staple (SI). From the average, a high intense positive signal was estimated for (*P,P,P*)-**1**, typical for helicene-based systems owing to a parallel orientation of the vectors. Therefore, the first eluted peak corresponds to the (*P,P,P*)-**1** enantiomer, displaying a *g*_abs_ value up to 2.33 × 10^–2^ in MeOH. This remarkable value, exceeding the typical threshold of 10^–3^ for simple organic molecules, can be attributed to the enhanced value of |*m*|, resulting from the large inner area of the compound with three full helical turns.

In the excited state, CPL spectra exhibited consistent profiles in all solvents, with MeOH yielding slightly higher intensities. CPL spectra of both (*P,P,P*) and (*M,M,M*)-**1** in MeOH are shown in Figure 3b, with a maximum *g*_lum_ value of 1.3 × 10^–2^ at 417 nm. The *g*_lum_ values, which depend on vibronic transitions (Figures S7–S8), remained consistently high across solvents, suggesting limited conformational flexibility.

As anticipated, the presence of alkynes connecting the system enhances the CPL signal. To gain insight into the origin of this improvement, we analyzed the HOMO–LUMO distribution and the corresponding transitions during the S_1_→S_0_ relaxation. Figure 4 clearly shows the helical distribution of orbitals and the associated electric and magnetic transition dipole moment densities. Notably, important differences emerge: whereas the emission process (Figure 4a right and 4c) displays a transition density concentrated almost entirely on the *o*-OPE moiety, the absorption process (Figure 4a center and 4b) involves both the *o*-OPE and [6]­helicene subunits. The magnetic transition dipole moment (|*m*|) is 3.75 × 10^–20^ erg G^–1^ for emission and 5.00 × 10^–20^ erg G^–1^ for absorption. This supports our hypothesis that transitions involving large internal areas lead to improved |*m*| values. Further analysis of the components independently (Figure S19 for S_0_→S_1_ and Figure S21 for S_1_→S_0_) reveals that the *o*-OPE unit has a |*m*| value of 4.11 × 10^–20^ erg G^–1^, similar to compound **1**, while the [6]­helicene has a small |*m*| value of only 0.2 × 10^–20^ erg G^–1^. This confirms that the chiroptical response is dominated by the alkyne-rich core. However, after S_1_ relaxation, orbital mixing between the two helical parts appears inefficient, unlike the absorptive process, indicating that the system minimizes energy by spatially bringing the spin-coupled electrons closer.

**Figure 4:**
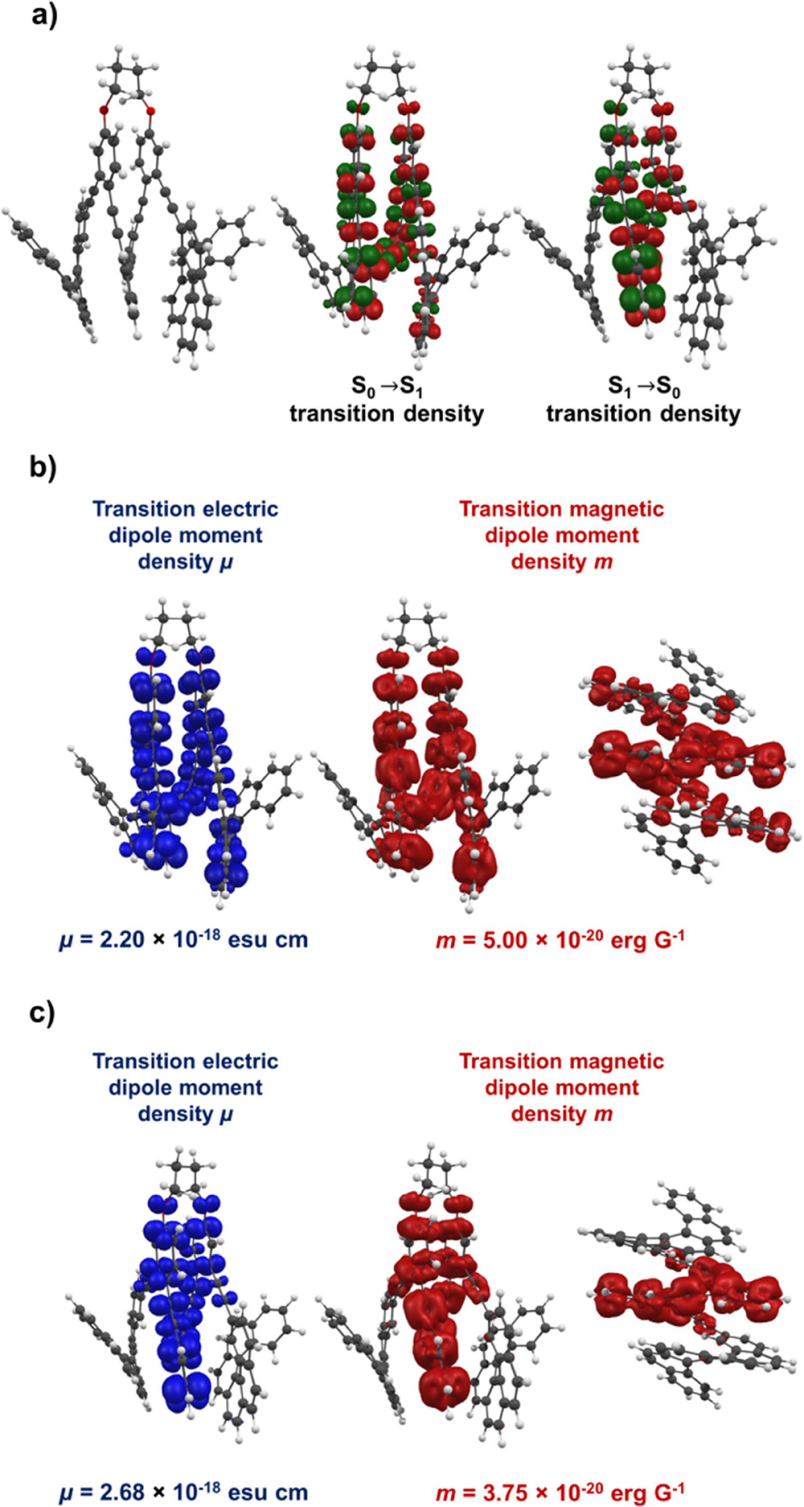
a) Molecular orbitals densities for the S_0_→S_1_ and S_1_→S_0_ transitions (contour value 0.001). Electric and magnetic transition dipole moment densities for b) S_0_→S_1_ and c) S_1_→S_0_ transitions (contour value 0.005).

To assess the generality of this trend, we conducted theoretical studies on systems ranging from benzene to [7]­helicene. For S_0_→S_1_ transitions, |*m*| increases with the size of the conjugated system up to [6]­helicene. Interestingly, for [7]­helicene the transition becomes localized on the helicene subunit, resulting in a drastic drop in |*m*| with a noticeable loss of emissive properties. (Figure S22). In contrast, for S_1_→S_0_ transitions, |*m*| remains in the (3.92–3.57) × 10^–20^ erg G^–1^ range and is localized on the *o*-OPE moiety (Figure S23). These results highlight the differences in the ground and excited states after relaxation, which has a profound impact on the CPL properties^45^ and reveal the essential role of [6]­helicene as the first configurationally stable member, also enabling efficient chirality transfer and strong chiroptical responses.

On the other hand, the calculated *g*_*lum*_ values for the different conformers ((4.1–6.9) × 10^–2^) are overestimated compared to the experimental one. Overestimations of *g*_*lum*_ values are not infrequent but mostly take place due to miscalculations of tiny values for |μ| and |*m*| in the range of 10^–21^, strongly affetting the |*m*|/|μ| ratio. Nevertheless, in this case the values are 268 × 10^–20^ esu cm and 3.75 × 10^–20^ erg G^–1^, respectively for the main conformer (Figure 4c). Therefore, there must be another source of discrepancy. Standard calculations typically rely on static models with a continuum solvent approximation, which fails to capture the complexity introduced by explicit solute–solvent interactions. To address this, we performed Molecular Dynamics (MD) simulations involving over 50 (*P*,*P*,*P*)-**1** molecules to sample the conformational distribution influenced by solvent dynamics. After 5 ns of simulation, all molecules were extracted and their *g*_lum_ values computed via DFT single-point calculations (see SI for details). Although this approach neglects the excited-state nature of solute–solvent interactions, it offers a practical compromise, enabling a statistically relevant *g*_lum_ estimation while avoiding the prohibitive computational cost of including explicit solvent molecules in full DFT calculations. We focused on MeOH as the solvent, and the results indicate that the conformations fluctuate around the energy minimum. In the cell, different conformations can be observed, with the semifolded conformers (Figure 2) accounting for a significant proportion of the molecules. All of them have been calculated presenting *g*_*lum*_ values from 5.44 × 10^–2^ to −5.62 × 10^–2^. When an average of such values is made, a more reliable and statistically robust *g*_*lum*_ value of 1.42 × 10^–2^ can be obtained, in excellent agreement with the experimental results. Interestingly when the same simulation workflow is made with the (*P,P,M*)-**1** diastereosiomer, an average *g*_*lum*_ value of zero is obtained considering the racemization of the central core. These results show that models that take into consideration solvent molecules in a more rigorous way that classical solvent continuum assumptions are required to understand the behavior of CPL emitters in real environments for dynamic systems.

In summary, we have developed a new class of fully π-conjugated helical architectures by combining configurationally stable [6]­helicenes with a flexible *o*-OPE unit. This design enables the formation of persistent, multiloop helical systems without the aid of metals, showing enhanced chiroptical properties in both ground and excited states. The resulting (*P*,*P*,*P*)/(*M*,*M*,*M*)-**1** compounds display significant values for the dissymmetric factors *g*_*abs*_ and *g*_*lum*_. A combined DFT and MD approach was used to elucidate their chiroptical behavior: DFT highlights the roles of helicity, rigidity, and π-conjugation, while MD reveals greater conformational flexibility in solution than static models suggest. This synergy offers a reliable computational framework to describe flexible CPL-active systems and supports the development of next-generation chiral optoelectronic materials. These findings provide valuable insights into the structure–property relationships governing circularly polarized emission and offer a promising platform for the design of advanced chiral optoelectronic materials.

## Supplementary Material



## Data Availability

The data underlying this study are available in the published article and its Supporting Information. Additional primary research data have been uploaded to a compliant third-party repository and are publicly available at the following DOI: https://doi.org/10.5281/zenodo.15535155.

## Notes

Detailed experimental and computational procedures, synthesis and characterization data for all compounds, methods and chromatograms for chiral separation, photophysical measurement data, Cartesian coordinates from quantum chemical calculations, and input files from molecular dynamics (MD) simulations (PDF)
