# Supplementary material for: Helical π‑Conjugated Structure Based on Complementary Helical Monomers with Circularly Polarized Luminescence
Source: Org Lett. 2025 Jul 10;27(29):7967–72. doi: 10.1021/acs.orglett.5c02299 (PMC12305635; doi:10.1021/acs.orglett.5c02299)
Supplement: Supplementary file 1 [file ol5c02299_si_001.pdf]

Supporting Information for:

Helical π-conjugated structure based on complementary helical monomers with circularly polarized luminescence

Rafael G. Uceda,[a] Ana M. Ortuño,[a] Luis Álvarez de Cienfuegos,[a] Federico Movilla[b], Antonio J. Mota,[c*] Delia Miguel[d*] and Juan M. Cuerva[a*]

[a]*Department of Organic Chemistry, Faculty of Sciences, Unidad de Excelencia de Química (UEQ), University of Granada. Avda. Fuente Nueva s/n, 18071 Granada, Spain.*
[b]*Department of Physical Chemistry, Faculty of Pharmacy, (UEQ) University of Granada. C. U. Cartuja, 18071 Granada, Spain*
[c]*Department of Inorganic Chemistry, Faculty of Sciences, Unidad de Excelencia de Química (UEQ), University of Granada. Avda. Fuente Nueva s/n, 18071 Granada, Spain.*

Table of Contents

1. General details. 2
2. Synthesis and characterization of new compounds. 3
3. HPLC traces. 11
4. Photophysical properties. 13
4.1. Absorbance and fluorescence measurement 13
4.2. CD and CPL measurement. 14
4.3. Quantum yields and fluorescence lifetimes 16
5. DFT calculations. 18
5.1. Conformational equilibrium 18
5.2. Chiroptical properties. 20
5.3. Effect of π-extension. 28
5.4. Cartesian coordinates of computed structures 29
6. Molecular dynamics simulations 102
6.1. Input files for MD simulations 105
7. References. 109

1. General details.

All reagents and solvents (AcOEt, Hexane, Et_3N, MeCN, DMF, DCM, MeOH) were purchased from standard chemical suppliers and used without further purification. Thin-layer chromatography analysis was performed on aluminium-backed plates coated with silica gel 60 (230-240 mesh) with F_{254} indicator. The spots were visualized with UV light (254 nm and 360 nm) and/or stained with phosphomolybdic acid (10% ethanol solution) and subsequent heating. Column chromatography purifications were performed with silica gel 60 (40-63 µm). ^{1}H- and ^{13}C-spectra were recorded on Bruker Avance Neo (500 MHz and 126 MHz, respectively) spectrometers at a constant temperature of 298 K. Chemical shifts are reported in ppm using residual solvent peak as reference ($CDCl_3$: δ = 7.26 ppm, CD_2Cl_2: δ = 5.3 ppm for ^{1}H and $CDCl_3$: δ = 77.0 ppm, CD_2Cl_2: δ = 53.5 ppm for ^{13}C). Data are reported as follows: chemical shift, multiplicity (s: singlet, d: doublet, t: triplet, q: quartet, m: multiplet, dd: doublet of doublet, ddd: doublet of doublets of doublets), coupling constant (*J* in Hz) and integration. Carbon multiplicities were determined by DEPT technique.

2. Synthesis and characterization of new compounds.

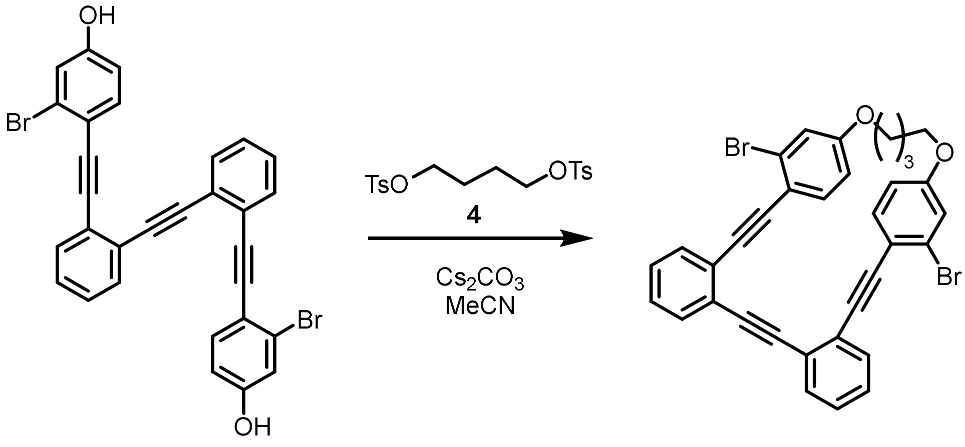

5 **3**

Compound **5** was prepared following described protocols.[1] The product showed NMR spectra identical to reported data. **^{1}H NMR (400 MHz, CD_3OD)**: δ 7.65 – 7.59 (m, 4H), 7.44 - 7.35 (m, 6H), 7.05 (d, *J*=2.4 Hz, 2H), 6.70 (dd, *J* =8.5, 2.4 Hz, 2H).

Compound **4** was prepared following described protocols.[2] The product showed NMR spectra identical to reported data. **^{1}H NMR (400 MHz, $CDCl_3$)**: δ 7.78 (d, *J* = 8.3 Hz, 2H), 7.37 (d, *J* = 8.0 Hz, 2H), 4.01 (t, *J* = 4.0 Hz, 4H), 2.48 (s, 6H), 1.72 (t, *J* = 4.0 Hz, 4H).

Compound **3**: Compound **4** (140 mg, 0.35 mmol, 1.0 eq.) and Cs_2CO_3 (275 mg, 0.84 mmol, 2.4 eq.) were placed in a two-neck round-bottom flask under inert atmosphere,. A solution of compound **5** (200 mg, 0.35 mmol, 1.0 eq.) in dry MeCN (100 mL) was then added. The reaction mixture was heated to reflux in an oil bath for 24 hours and subsequently concentrated under reduced pressure. The crude residue was supported on celite and purified by flash column chromatography (SiO_2, Hexane/AcOEt, 8:2), affording a mixture of product **3** and residual compound **4**. To separate them, a precipitation method was employed: the solid was dissolved in a minimal amount of dichloromethane, followed by addition of hexane, leading to the precipitation of product **3** as a white solid (120 mg, 55%).**^{1}H NMR (500 MHz, $CDCl_3$)** δ 7.72 – 7.62 (m, 4H), 7.37 – 7.32 (m, 4H), 7.22 (d, *J* = 8.6 Hz, 2H), 6.90 (d, *J* = 2.3 Hz, 2H), 6.26 (dd, *J* = 8.6, 2.4 Hz, 2H), 4.22 (s, 4H), 1.95 (s, 4H). **^{13}C NMR (126 MHz, $CDCl_3$)** δ 157.6 (C), 134.1 (CH), 133.4 (CH), 133.0 (CH), 128.1 (CH), 127.8 (CH), 126.4 (C), 125.2 (C), 124.8 (C), 117.1 (C), 116.8 (CH), 115.3 (CH), 92.6 (C), 92.2 (C), 91.3 (C), 67.2 (CH_2), 23.7(CH_2). **HRMS (ESI) m/z:** $[M+Na]^+$ Calcd for $C_{34}H_{22}O_2Br_2Na$ 644.9867; Found: 644.9878.

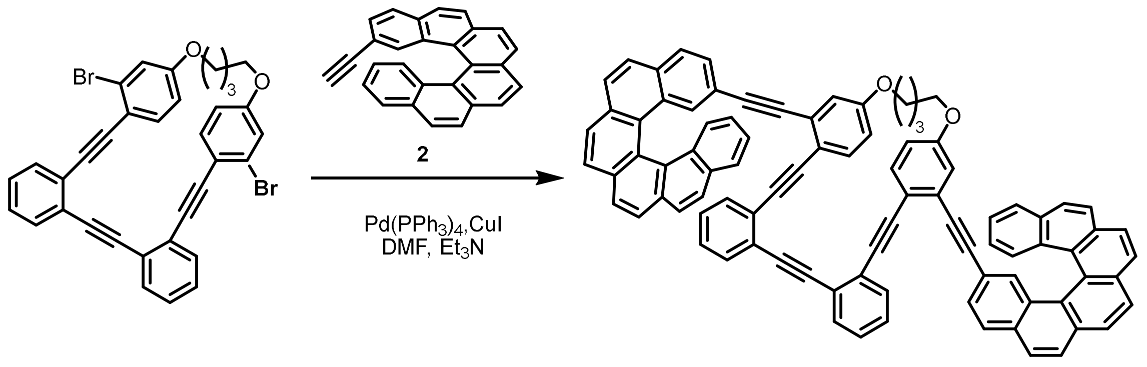

3 **1**

Compound **2** was prepared following described protocols.[3] The product showed NMR spectra in agreement to reported data. **^{1}H NMR (400 MHz, $CDCl_3$)**: δ 8.04 – 7.99 (m, 3H), 7.99 – 7.93 (m, 4H), 7.87 (dd, *J* = 8.3, 1.8 Hz, 2H), 7.79 (bsignal, 1H), 7.76 (d, *J* = 8.2 Hz, 1H), 7.57 (dd, *J* = 8.5, 1.0 Hz, 1H), 7.33 – 7.27 (m, 2H), 6.73 (ddd, *J* = 8.4, 6.9, 1.4 Hz, 1H), 2.72 (s, 1H).

Compound **1**: Compound **3** (97 mg, 0.156 mmol, 1 eq.), $Pd(PPh_3)_4$ (10 mg, 0.0078 mmol, 0.05 eq.), and CuI (1.5 mg, 0.078 mmol, 0.05 eq.) were introduced into a round-bottom flask under argon atmosphere. The mixture was dissolved in dry DMF (10 mL) and NEt_3 (1 mL) and heated in an oil bath to 85°C. Alkyne **2** (165 mg, 0.468 mmol, 3 eq.) was added dropwise dissolved in dry DMF (5 mL). The reaction was stirred at 85°C for 10 hours. After this time, the reaction mixture was extracted with brine to remove DMF, and the solvent was evaporated under reduced pressure. The crude product was then purified by flash column chromatography (SiO_2, Hexane/AcOEt: 100/0 → 50/50), affording the desired product **1** as a yellow solid (34 mg, 19%). The diastereoisomers were subsequently separated on HPLC (see section 3). (*P*,*P*,*P*)/(*M*,*M*,*M*)-**1** **^{1}H NMR (500 MHz, CD_2Cl_2)** δ 8.02 (d, *J* = 8.1 Hz, 2H), 7.95 (d, *J* = 8.2 Hz, 2H), 7.93 (d, *J* = 8.1 Hz, 2H), 7.89 (d, *J* = 8.5 Hz, 2H), 7.75 (dd, *J* = 8.5, 6.2 Hz, 4H), 7.72 (m, 1H), 7.70 (d, *J*=5.0 Hz, 2H), 7.59 (d, *J*=5.0 Hz, 2H), 7.49 (d, *J* = 8.1 Hz, 2H), 7.43 (dd, *J* = 8.6, 4.6 Hz, 4H), 7.11 (m, 2H), 7.08 (d, *J* = 7.4 Hz, 2H), 7.04 (d, *J* = 8.5 Hz, 2H), 6.91 (d, *J* = 8.0 Hz, 2H), 6.88 (d, *J* = 7.8 Hz, 2H), 6.83 (t, *J* = 7.5 Hz, 2H), 6.63 (t, *J* = 7.5 Hz, 2H), 6.55 (t, *J* = 7.7 Hz, 2H), 6.44 (d, *J* = 2.6 Hz, 2H), 6.22 (dd, *J* = 8.6, 2.6 Hz, 2H), 4.27 (m, 2H), 4.17 (m, 2H), 2.07 (m, 2H), 1.85 (m, 2H). **^{13}C NMR (126 MHz, CD_2Cl_2)** δ 157.1 (C), 133.5 (CH), 133.3 (C), 133.0 (CH), 132.4 (CH), 132.2 (CH), 131.8 (C), 131.5 (C), 131.4 (C), 129.7 (C), 129.3 (C), 128.2 (CH), 128.0 (CH), 127.8 (CH), 127.7 (CH), 127.7 (C), 127.5 (CH), 127.4 (CH), 127.4 (CH), 127.4 (CH), 127.3 (CH), 127.2 (CH), 127.1 (CH), 127.0 (CH), 126.7 (C), 126.6 (CH), 126.2 (C), 126.1 (CH), 125.7 (CH), 125.2 (C), 124.5 (CH), 124.5 (C), 124.4 (C), 124.0 (C), 119.6 (C), 117.7 (C), 116.4 (CH), 115.8 (CH), 93.6 (C), 92.5 (C), 91.9 (C), 90.8 (C), 87.9 (C), 67.5 (CH_2), 29.8 (CH_2). **HRMS (MALDI) m/z:** $[M]^+$ Calcd for $C_{90}H_{52}O_2$ 1164.3962; Found: 1164.3955. (*P*,*P*,*M*)-**1** **^{1}H NMR (500 MHz, CD_2Cl_2)** δ 8.10 (d, *J* = 8.2 Hz, 2H), 8.04 (d, *J* = 2.9 Hz, 2H), 8.02 (d, *J* = 2.8 Hz, 2H), 7.94 (d, *J* = 8.5 Hz, 2H), 7.81 (d, *J*=7.0, 2H), 7.78 (d, *J* = 9.4 Hz, 4H), 7.72 (d, J=9.1Hz, 4H), 7.50 (d, *J*=8.6 Hz, 2H), 7.47 (d, *J* = 8.5 Hz, 2H), 7.43 (d, *J* = 8.2 Hz, 2H), 7.17-7.13 (m, 2H), 7.08 (d, *J* = 7.6 Hz, 2H), 7.06 (d, *J* = 8.7 Hz, 2H), 6.85 – 6.81 (m, 2H), 6.80 – 6.74 (m, 4H), 6.67 – 6.61 (m, 4H), 6.51 (d, *J* = 2.6 Hz, 2H), 6.31 (dd, *J* = 8.5, 2.6 Hz, 2H), 4.35 – 4.25 (m, 4H), 2.11 – 2.02 (m, 4H). **^{13}C NMR (126 MHz, CD_2Cl_2)** δ 157.1 (C), 133.2 (CH), 133.1 (C), 132.9 (CH), 132.4 (CH), 132.1 (C), 131.9 (CH), 131.6 (C), 131.3 (C), 131.2 (C), 129.5 (C), 129.1 (C), 128.2 (CH), 127.9 (CH), 127.7 (CH), 127.6 (C), 127.6 (C), 127.6 (CH), 127.4 (CH), 127.2 (CH), 127.2 (CH), 127.1 (CH), 127.1 (CH), 127.1 (CH), 126.8 (CH), 126.6 (C), 126.6 (CH), 126.4 (CH), 125.9 (CH), 125.7 (CH), 124.9 (C), 124.4 (CH), 124.2 (C), 124.0 (C), 119.6 (C), 117.6 (C), 116.7 (CH), 114.9 (CH), 93.6 (C), 92.3 (C), 91.6 (C), 90.5 (C), 87.8 (C), 67.4 (CH_2), 29.7 (CH_2). **HRMS (MALDI) m/z:** $[M]^+$ Calcd for $C_{90}H_{52}O_2$ 1164.3962; Found: 1164.3942.

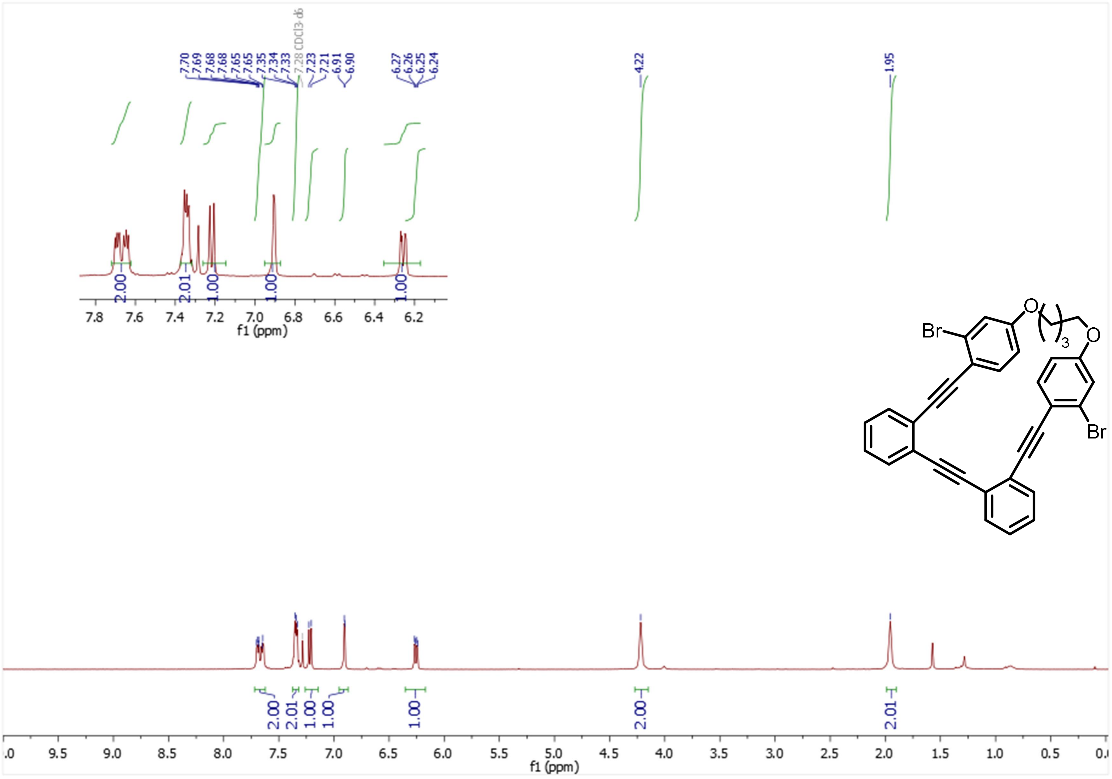

^{1}H-NMR spectra of **3** ($CDCl_3$, 500 MHz).

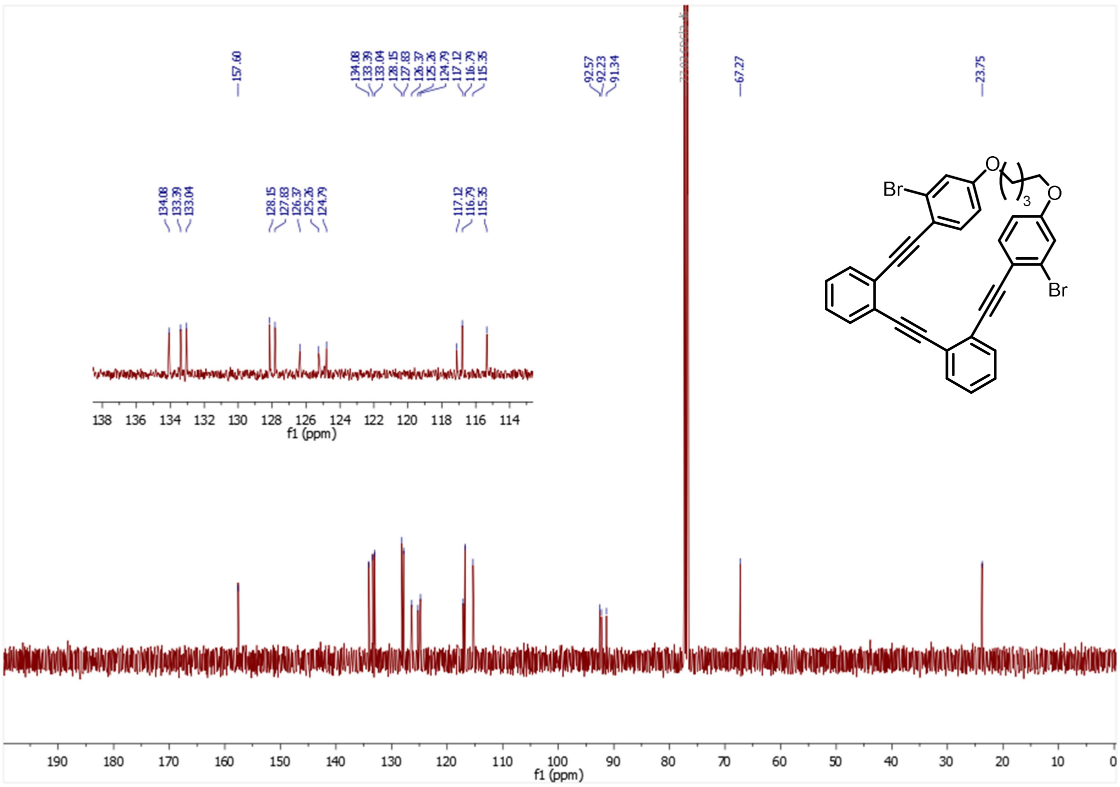

^{13}C-NMR spectra of **3** ($CDCl_3$, 126 MHz).

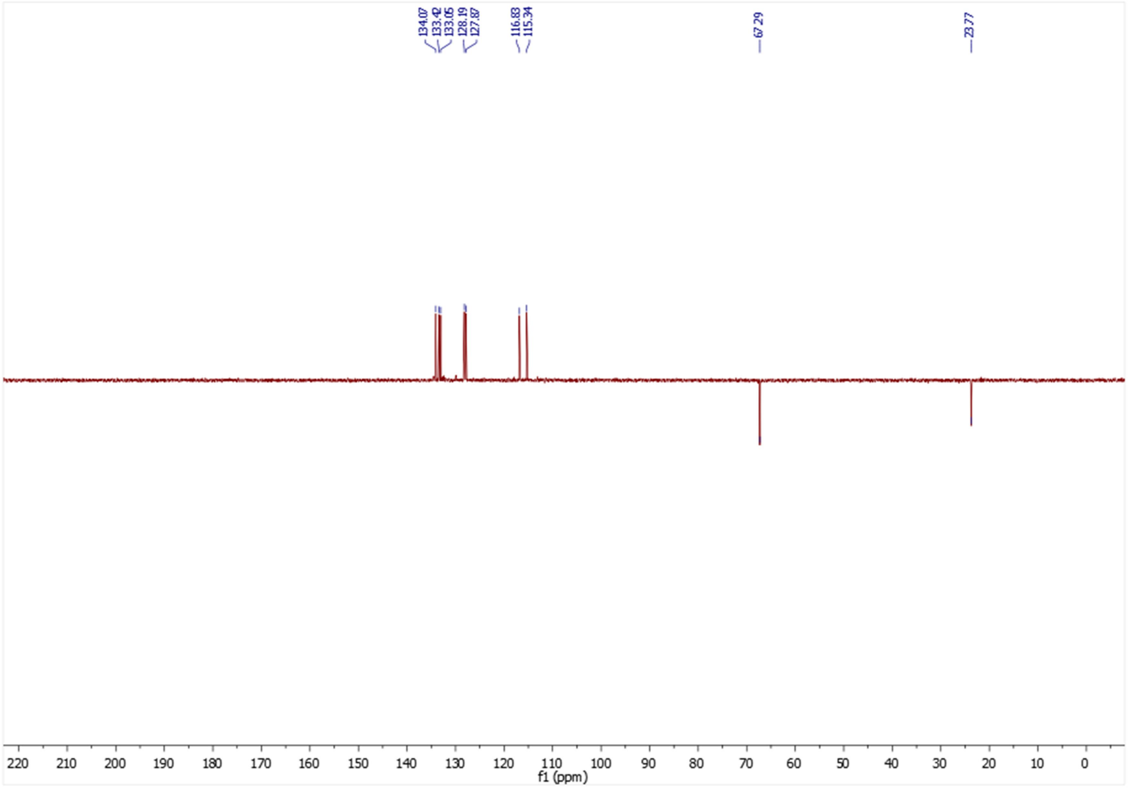

DEPT-135 NMR spectra of **3** ($CDCl_3$, 126 MHz).

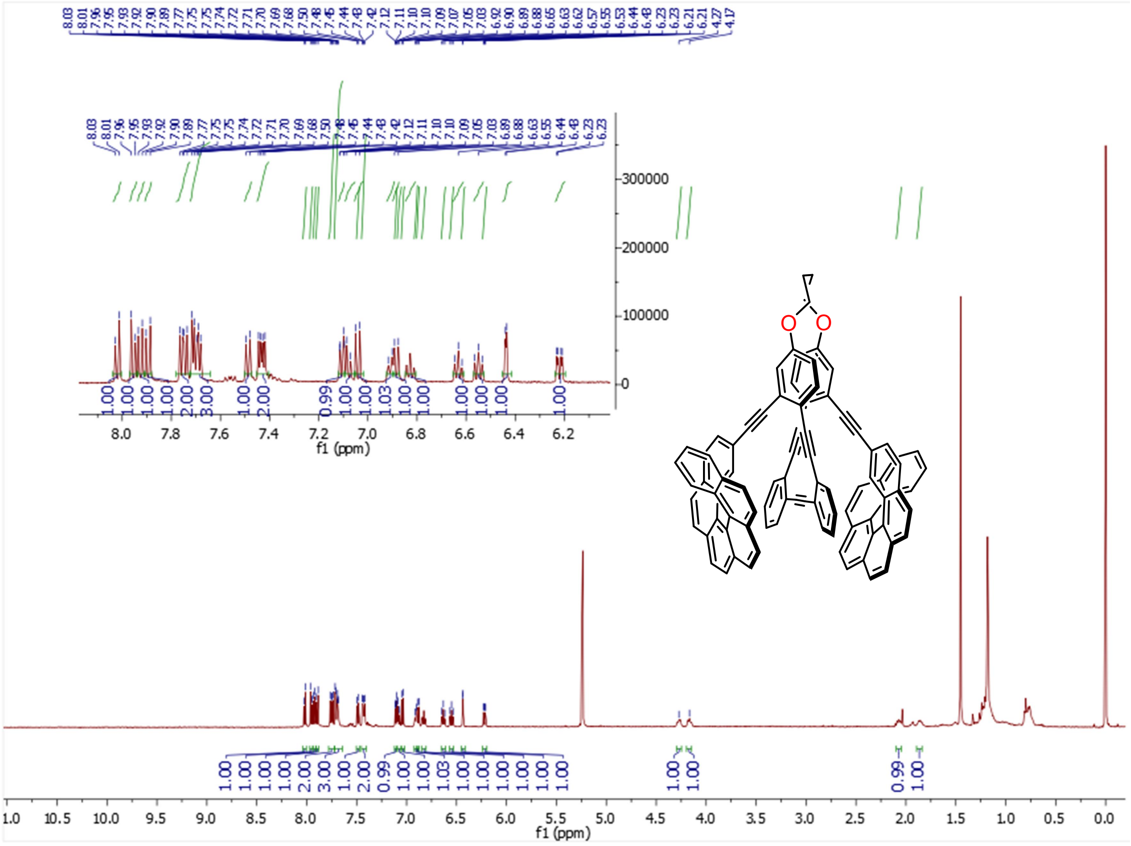

^{1}H-NMR spectra of (*P*,*P*,*P*)/(*M*,*M*,*M*)-**1** (CD_2Cl_2, 500 MHz).

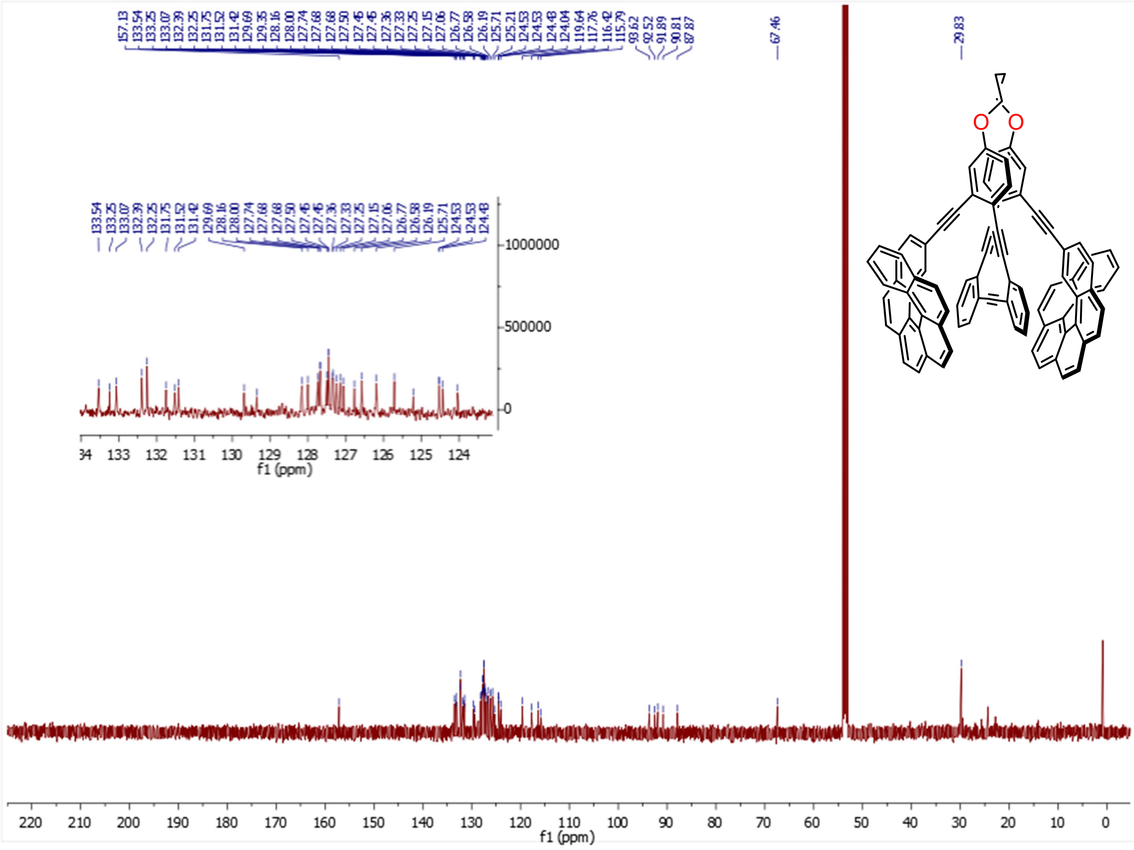

^{13}C-NMR spectra of (*P*,*P*,*P*)/(*M*,*M*,*M*)-**1** (CD_2Cl_2, 126 MHz).

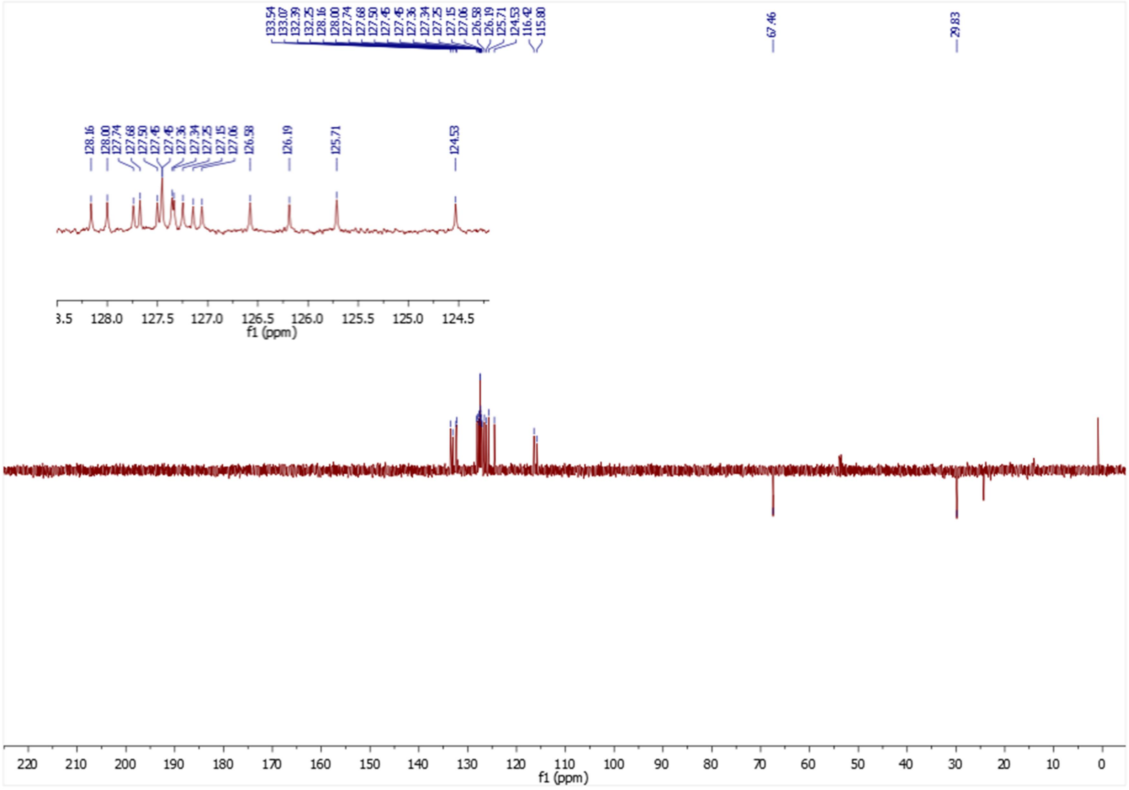

DEPT-135 NMR of (*P*,*P*,*P*)/(*M*,*M*,*M*)-**1** (CD_2Cl_2, 126 MHz).

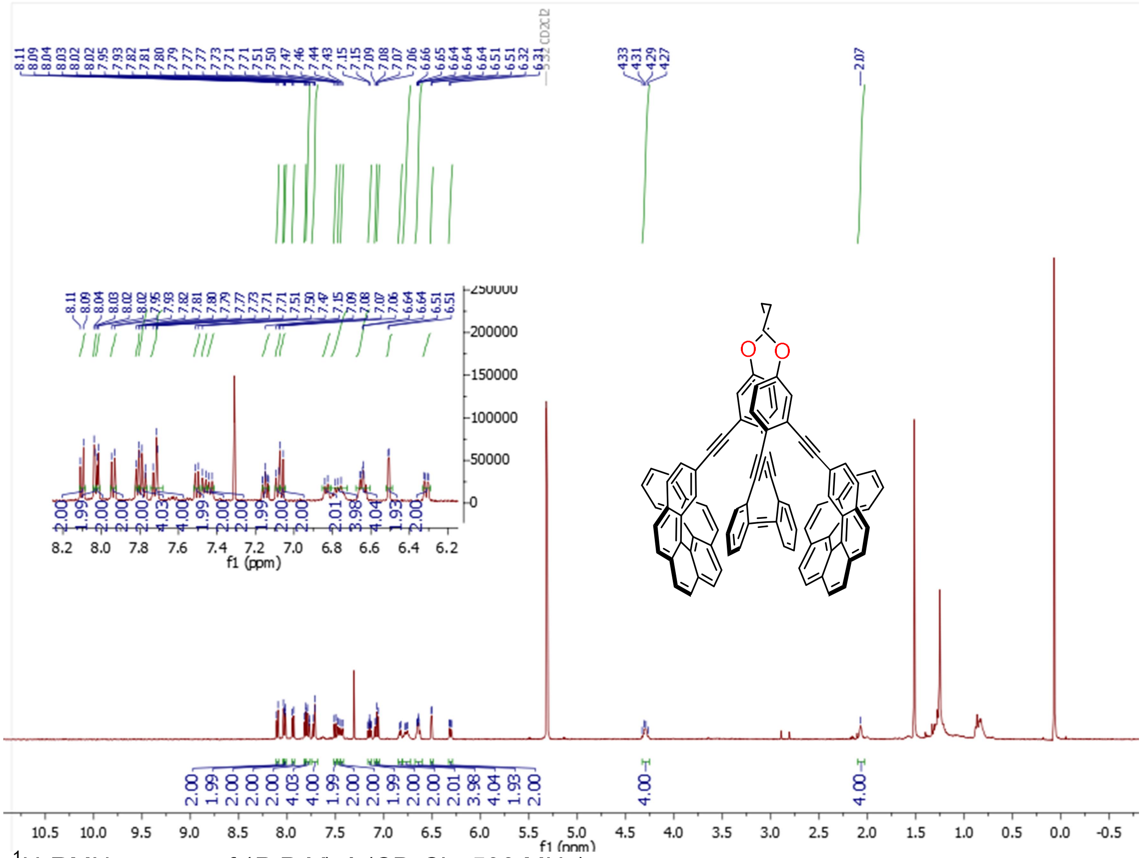

^{1}H-RMN spectra of (*P*,*P*,*M*)-**1** (CD_2Cl_2, 500 MHz).

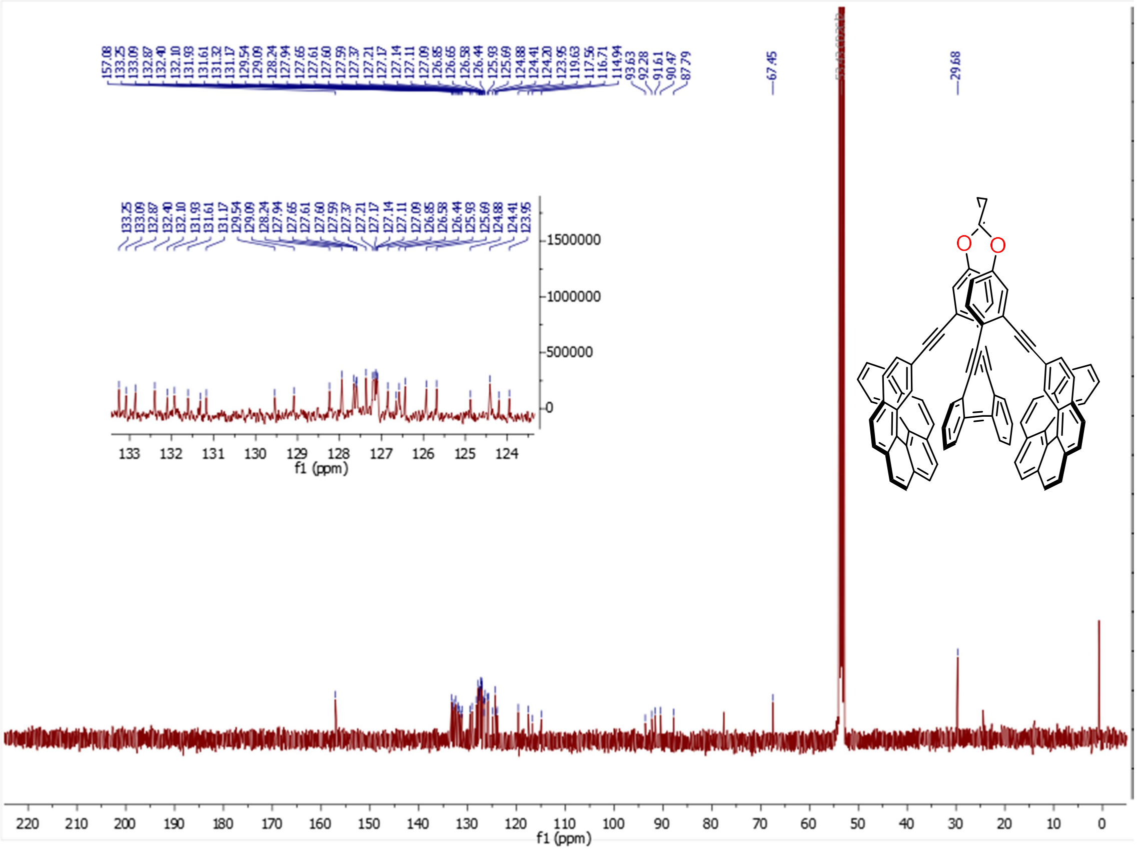

^{13}C-RMN spectra of (*P*,*P*,*M*)-**1** (CD_2Cl_2, 126 MHz).

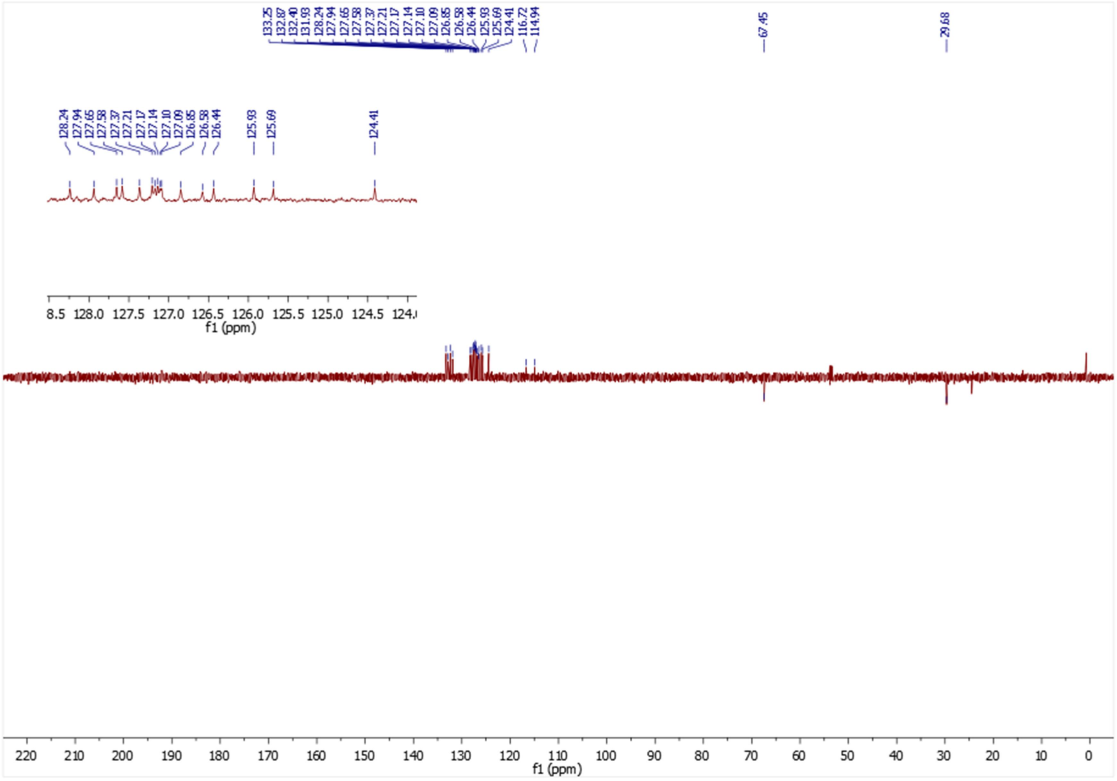

DEPT-135 NMR of (*P*,*P*,*P*)/(*M*,*M*,*M*)-**1** (CD_2Cl_2, 126 MHz).

3. HPLC traces.

HPLC analysis were performed on an Agilent 1260 series equipped with the following modules: quaternary pump (G7111B 1260 Quat Pump), automatic sample injector (G2258A 1260 DL ALS), column thermostat (G1316A 1260 TCC), DAD detector (G7115A 1260 DADWR) and an automatic sample collector (G1364C 1260 FC-AS). CHIRALPAK® 250-10 mm packed with silica gel (5 μm) was used as semipreparative chiral column. The column temperature was set at 20 ˚C and the mobile phase used was Hexane:DCM (75:25). The wavelength selected for the peak detection was 300 nm and the flow was constant during the operation: 3.4 mL/min.

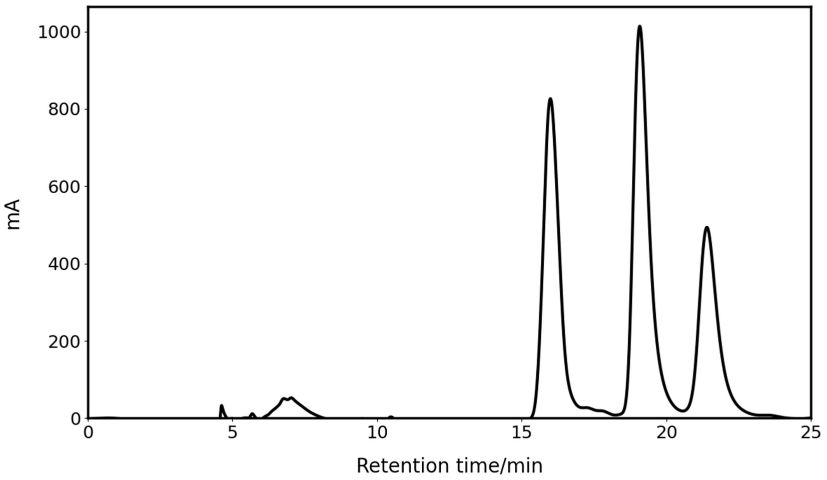

Figure S1: HPLC chromatogram of compound **1**. The chromatogram shows three major peaks with area percentages of 31.31%, 40.01%, and 22.66%, respectively.

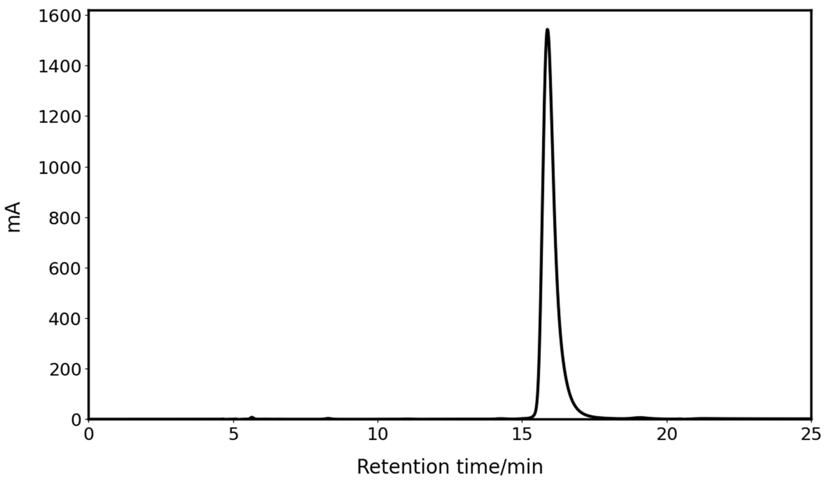

Figure S2: HPLC chromatogram of peak 1 of compound (*P*,*P*,*P*)-**1** (purity = 99.09%).

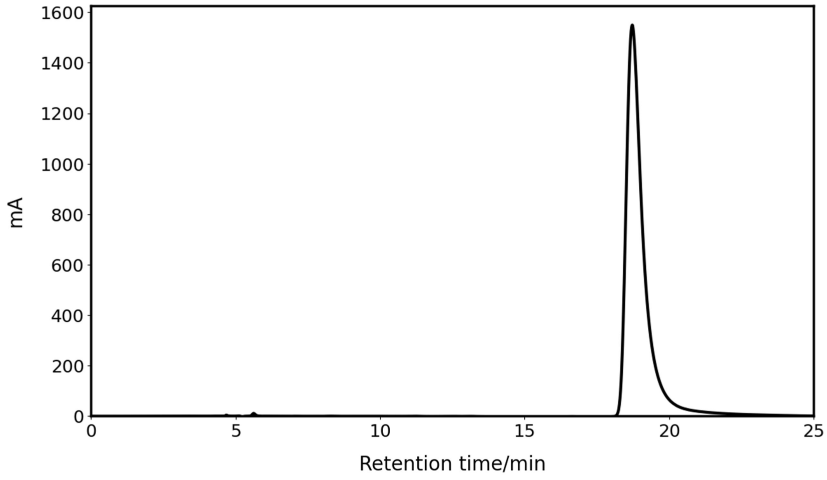

Figure S3: HPLC chromatogram of peak 2 of compound (*P*,*P*,*M*)-**1** (purity = 99.73%).

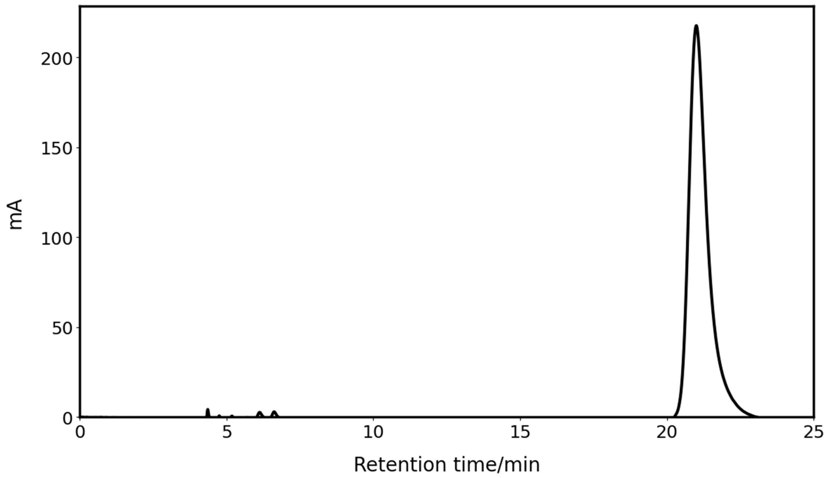

Figure S4: HPLC chromatogram of peak 3 of compound (*M*,*M*,*M*)-**1** (purity = 98.17%).

4. Photophysical properties.

4.1. Absorbance and fluorescence measurement.

Absorption and emission measurements were performed in an Olis DSM172 spectrophotometer using a 1.0 cm path-length quartz cell and $2.5 \cdot 10^{-5}$ M solutions of the ligands in HPLC grade solvents. A xenon lamp of 150 W was used for both measurements. Emission was measured using a fixed wavelength at 300 nm as the excitation source.

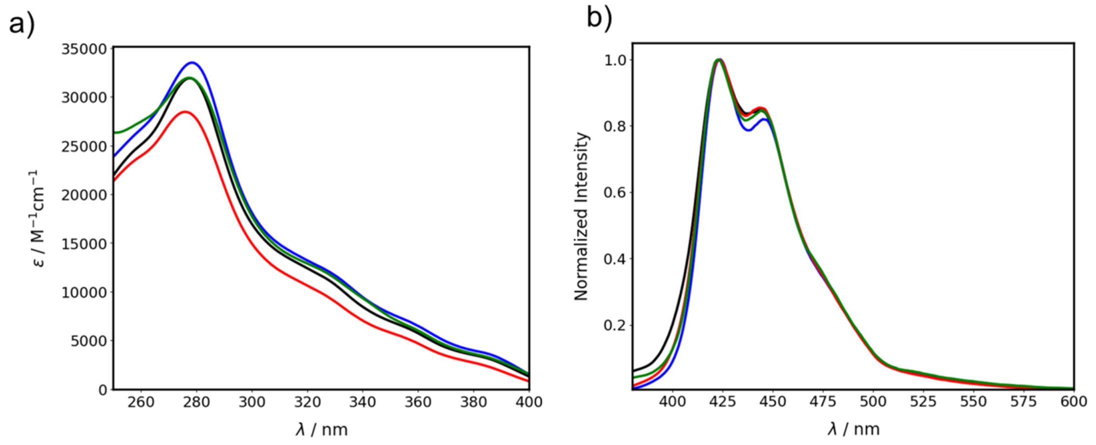

Figure S5: a) Absorption and b) fluorescence emission (λ_{exc} = 300 nm) spectra of compound **1** in hexane (black), dichloromethane (blue), methanol (red) and acetonitrile (green).

4.2. CD and CPL measurement.

Measurements were performed in an Olis DSM172 spectrophotometer using a 1.0 cm path-length quartz cell and $2.5 \cdot 10^{-5}$ M solutions of the ligands in HPLC grade solvents. A xenon lamp of 150 W was used for both measurements. Emission was recorded using a fixed wavelength of 300 nm. CD spectra of (*P*,*P*,*P*)/(*M*,*M*,*M*)-**1** was collected in different solvents by accumulating 30 scans. In all the cases a fixed slit-width of 1 mm and 0.1s of integration time were selected. CPL spectra of compounds 1 were collected in the same solvents by accumulating 150 scans with 1 s of integration time.

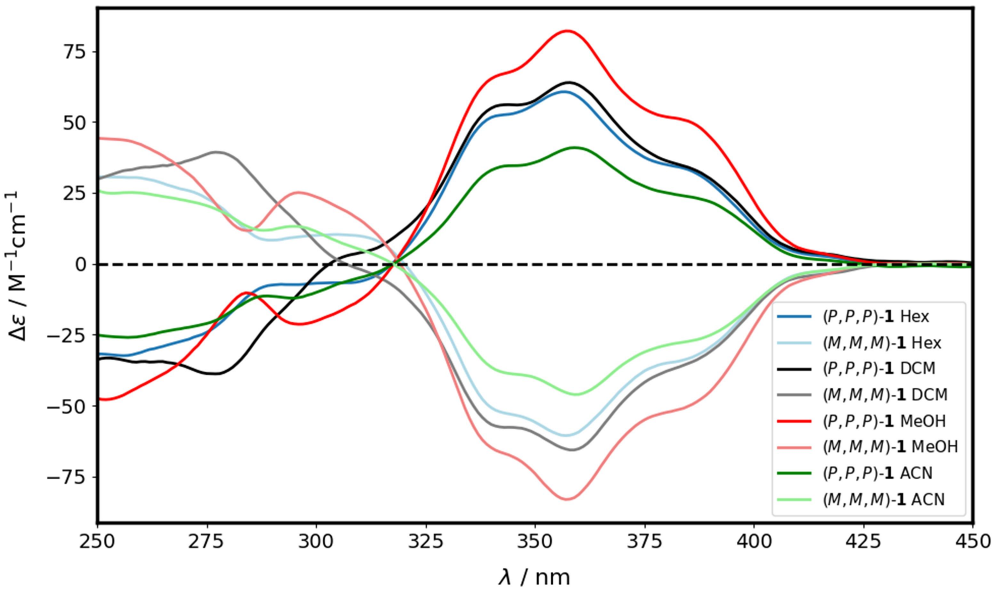

Figure S6: CD spectra of both enantiomers of compound **1** in different solvents.

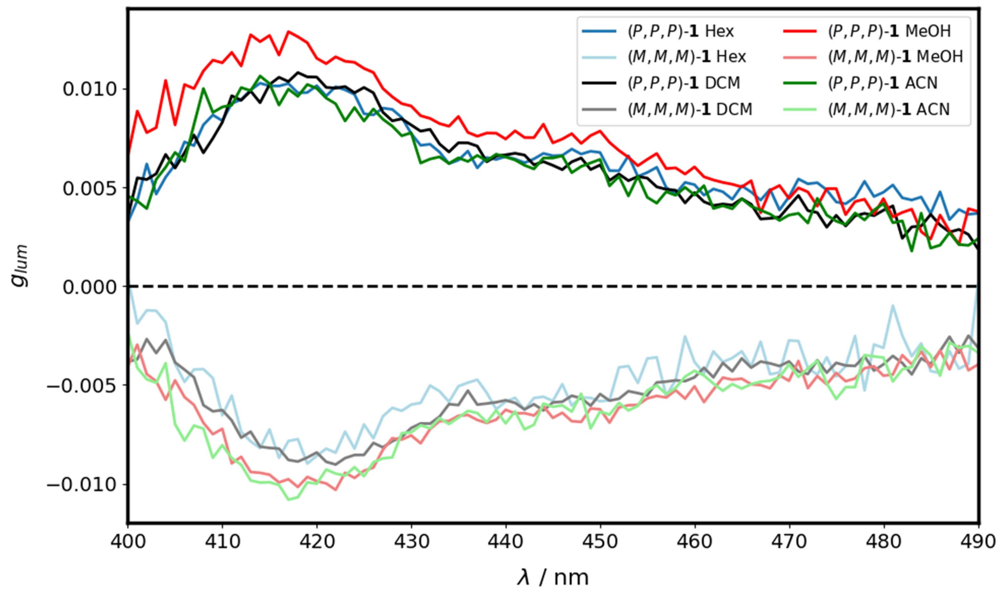

***Figure S7**:* g_{lum} values obtained for both enantiomers of compound **1** in different solvents.

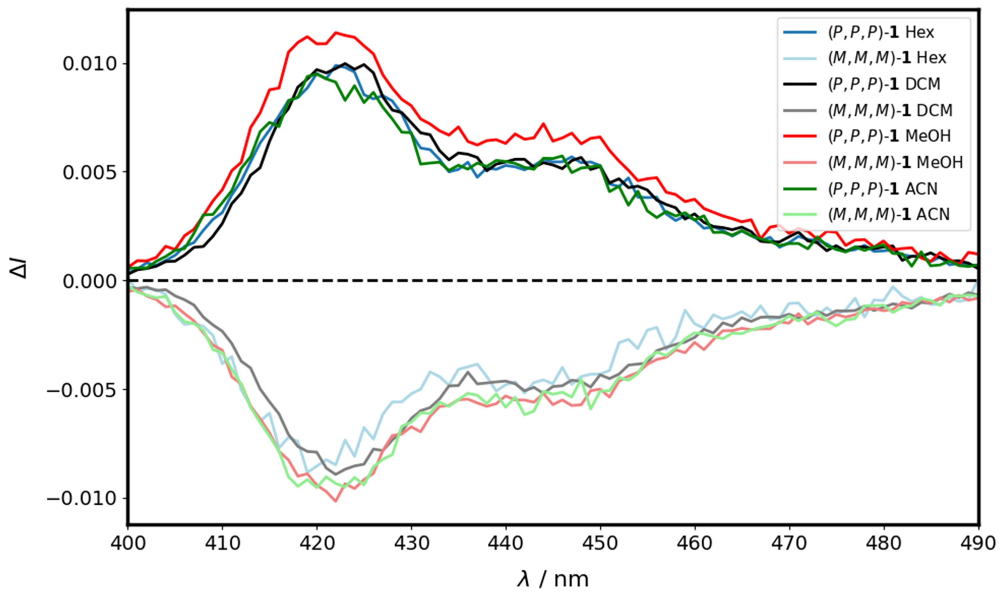

***Figure S8**:* *ΔI* values obtained both enantiomers of compound **1** in different solvents.

Table S1: Values of dissymmetry factor g_{abs} for compound **1**.

Solvent	(*P,P,P*) g_{abs}	λ/nm	(*M,M,M*) g_{abs}	λ/nm
Hexane	$1.00 \cdot 10^{-2}$	395	$-7.35 \cdot 10^{-3}$	398
DCM	$1.02 \cdot 10^{-2}$	397	$-9.83 \cdot 10^{-3}$	394
MeOH	$2.33 \cdot 10^{-2}$	400	$-2.40 \cdot 10^{-2}$	400
ACN	$5.65 \cdot 10^{-3}$	395	$-7.67 \cdot 10^{-3}$	399

Table S2: Values of dissymmetry factor g_{lum} for compound **1**.

Solvent	(*P,P,P*) g_{lum}	λ/nm	(*M,M,M*) g_{lum}	λ/nm
Hexane	$1.03 \cdot 10^{-2}$	414	$-8.98 \cdot 10^{-3}$	419
DCM	$1.08 \cdot 10^{-2}$	418	$-9.03 \cdot 10^{-3}$	422
MeOH	$1.29 \cdot 10^{-2}$	417	$-1.03 \cdot 10^{-2}$	422
ACN	$1.06 \cdot 10^{-2}$	414	$-1.08 \cdot 10^{-2}$	417

4.3. Quantum yields and fluorescence lifetimes.

Time-resolved fluorescence decay traces were collected via the time-correlated single photon counting (TCSPC) method using a FluoTime 200 fluorometer (PicoQuant GmbH). The excitation source was a 320 nm LED using a 20 MHz excitation frequency. The full width at half maximum (fwhm) of the laser pulses was around 40 ps. The fluorescence emission was collected at a 90° geometry, focused at the detector after crossing through a polarizer (set at the magic angle), 2 mm slits, and a 2 nm bandwidth monochromator. TCSPC was achieved by a TimeHarp200 board, set at 36 ps/channel. Fluorescence decay traces were collected for the necessary time to reach 20000 counts at the peak channel. For the analyzed compounds, decay traces were collected at three different wavelengths within ± 5 nm (Dl = - 5, 0, +5 nm) of the maximum of the main emission peak.

The fluorescence decay traces were fitted to a bi-exponential function by using an iterative reconvolution method based on the Levenberg-Marquardt algorithm for nonlinear least-squares error minimization deconvolution (FluoFit 4.4 package, PicoQuant GmbH). For each sample, the decay traces were fitted globally with the decay times linked as shared parameters, whereas the pre-exponential factors were local adjustable parameters. The quality of fittings was assessed by the value of the reduced chi-squared, χ^2, parameter and random distributions of the weighted residuals and the autocorrelation functions.

Quantum yields were determined by measuring both absorbance and fluorescence of compound (*P*,*P*,*P*)-**1** in different solvents, using quinine sulfate in 0.1 M H_2SO_4 as standard (Φ_r = 0.54). For the relative determination of the fluorescence quantum yield Φ in a series of solvents, eq. 1 was used.[4,5]

$$\Phi_x = \Phi_r \times \frac{F_x}{F_r} \times \frac{1 - 10^{-Ar(\lambda_{ex})}}{1 - 1 \quad ^{-Ax(\lambda_{ex})}} \times \frac{n_x^2}{n_r^2} \qquad \text{(Eq. 1)}$$

The subscripts *x* and *r* refer respectively to the sample and a reference (standard) fluorophore with known quantum yield Φ_r in a specific solvent; *F* stands for the spectrally corrected, integrated fluorescence spectra; $A(\lambda_{ex})$ denotes the absorbance at the used excitation wavelength λ_{ex}; and *n* represents the refractive index of the solvent (in principle at the average emission wavelength). To minimize inner filter effects, the absorbance at the excitation wavelength λ_{ex} was kept under 0.1. The measurements were performed using 10×10 mm cuvettes.

Table S3 summarized the fluorescence lifetimes and quantum yields obtained for described compound. This value, along with those measured in the previously described sections, is used to calculate the brightness B_{CPL},[6] defined as $B_{CPL} = \varepsilon_\lambda \times \Phi_F \times |g_{lum}|/2$, being ε_λ, the molar extinction coefficient at the excitation wavelength λ; Φ_F, the fluorescence quantum yield; and $|g_{lum}|$, the emission dissymmetry factor. The B_{CPL} in different solvent are reported in Table S4.

Table S3: Values of quantum yields Φ_F and fluorescence lifetimes τ for compound **1**.

**Solvent**	**Φ_F / %**	**τ_1 / ns**	**τ_2 / ns**	**τ_{av} / ns**
Hexane	1.8±0.1	7.96±0.03	1.86±0.17	7.54
DCM	3.8±0.3	10.52±0.04	2.03±0.14	10.26
ACN	4.7±0.7	9.84±0.04	2.28±0.15	9.26
MeOH	2.5±0.2	9.88±0.04	1.73±0.20	9.61

Table S4: Values of brightness B_{CPL} for compound **1**.

**Solvent**	**ε_λ/ M^{-1} cm^{-1}**	**Φ_F**	**g_{lum}**	**B_{CPL}/ M^{-1} cm^{-1}**
Hexane	16978	0.018	$1.03 \cdot 10^{-2}$	1.57
DCM	18140	0.038	$1.08 \cdot 10^{-2}$	3.72
ACN	17766	0.047	$1.29 \cdot 10^{-2}$	2.41
MeOH	14962	0.025	$1.06 \cdot 10^{-2}$	4.43

5. DFT calculations.

Theoretical calculations were performed to gain insight into the conformational equilibrium and chiroptical properties of compound **1**. All calculations were carried out using Gaussian 16,[7] considering the (*P,P,P*) enantiomer. The geometries considered can be found in the ZENODO repository by consulting the following link: https://doi.org/10.5281/zenodo.15535155.

5.1. Conformational equilibrium.

Initially, a conformational search was conducted using CREST,[8] which revealed that within a 3 kcal·mol^{-1} window only three conformers were present (**I**, **II**, **III**), all of which exhibited a fully folded molecular structure with differences arising from the staple conformation (Figure S9). Expanding the window to 6 kcal·mol^{-1} led to additional folded structures (IV) and semi-folded conformers (V) (Figures S9 and S10).

Subsequent DFT calculations employing the well-considered ωB97X-D/6-31G(d,p) level of theory confirmed these findings, demonstrating the efficient folding of the molecule and the chirality transfer process from the helicenes to the *o*-OPE. Additionally, further calculations on different semi-folded conformers revealed significant energy differences (nearly 10 kcal/mol) (Figure S10). Same results were found for the (*P,M,P*) conformer, in which the [6]helicenes would not induce a defined configuration in the *o*-OPE (Figure S11). These results highlight both the efficient chiral induction of the helix by the [6]helicene and the greater stability of the folded structures.

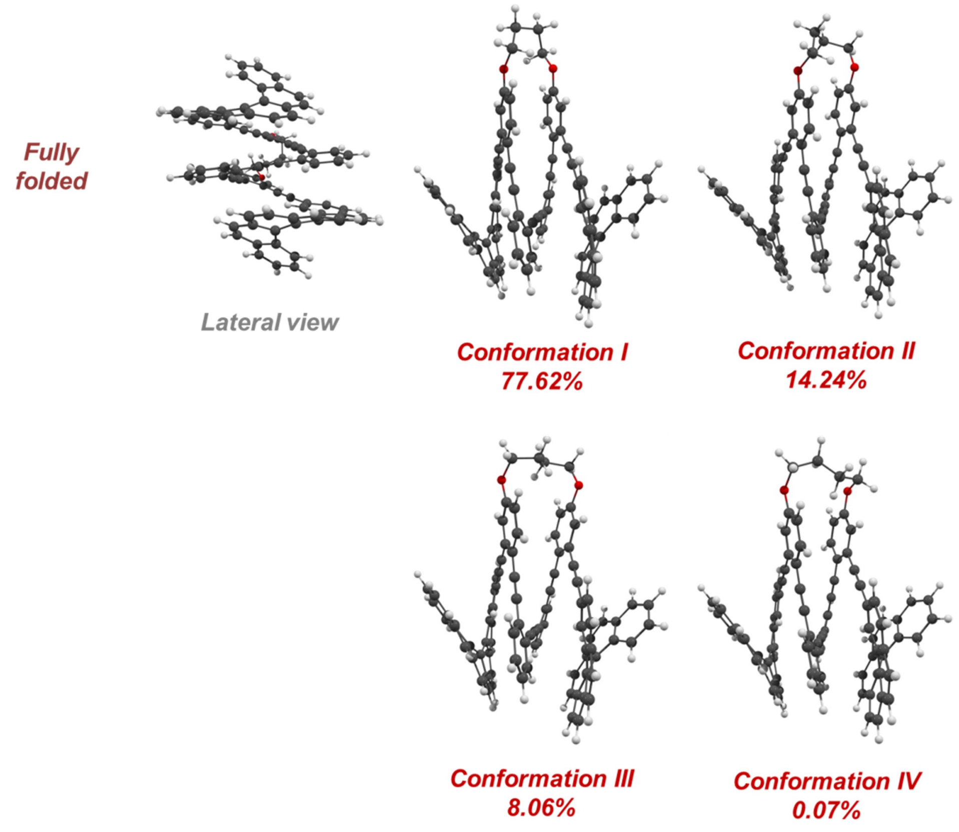

Figure S9: Fully folded conformers of (*P*,*P*,*P*)-**1**.

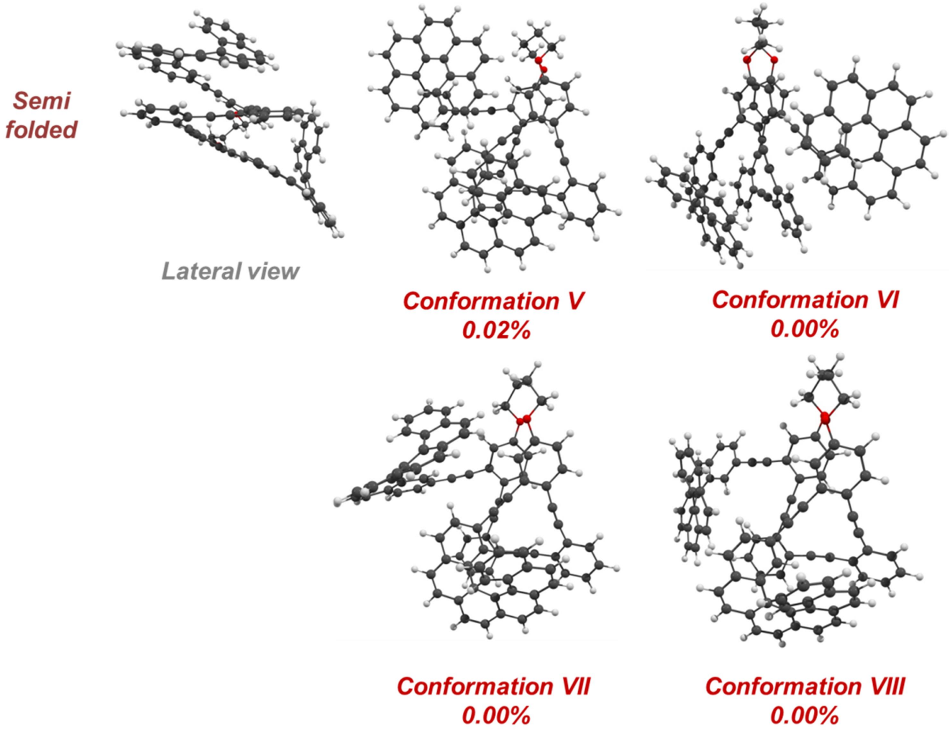

Figure S10: Semi folded conformers of (*P*,*P*,*P*)-**1.**

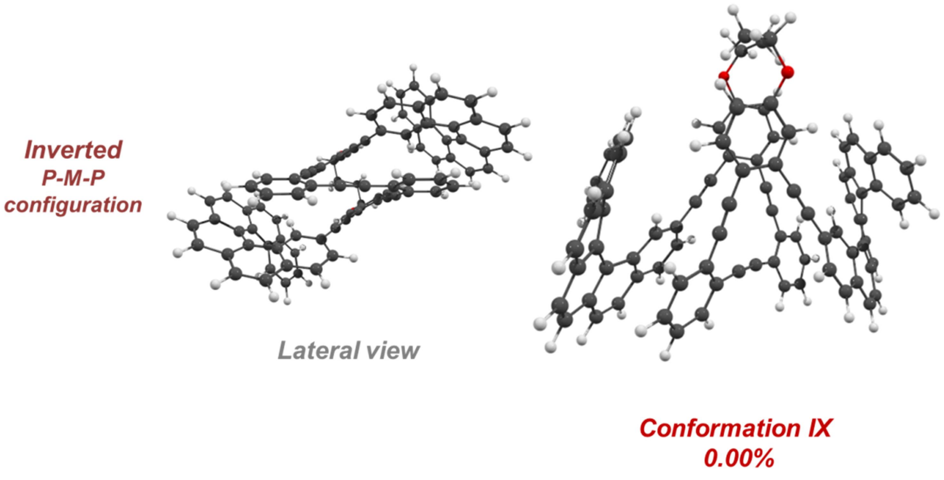

Figure S11: (*P*,*M*,*P*)-**1** conformer.

5.2. Chiroptical properties.

Initially, the chiroptical properties of the most populated conformer were evaluated using different functionals: B3LYP,[9] M06,[10] M06-2X,[10] ωB97X-D,[11] and CAM-B3LYP[12] (Figures S12-16) together with 6-31G(d,p) as basis set.[13] In all cases, implicit solvation in MeOH was considered and 50 vertical transitions were computed. All functionals accurately predicted the intensity of the experimentally observed signals; however, those with a low percentage of HF exchange, such as B3LYP and M06 (Figures S12 and S13), failed to reproduce the correct wavelengths. In contrast, long-range corrected functionals such as ωB97X-D and CAM-B3LYP accurately predicted both the transition intensities and the wavelengths at which they occur (Figures S14-S16). For this reason, the ωB97X-D functional was chosen to gain deeper insight into the chiroptical properties (Figure S16).

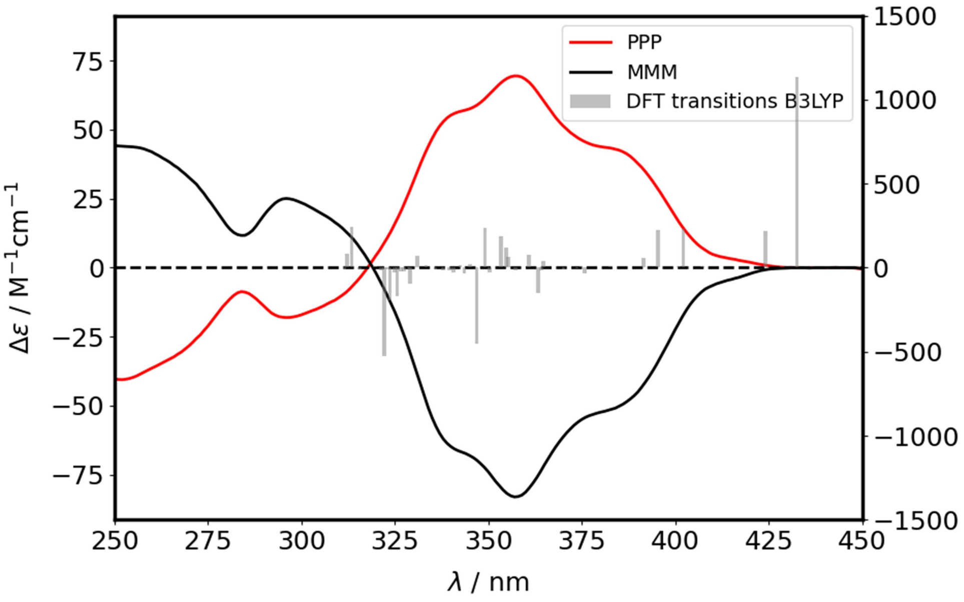

Figure S12: Experimental ECD in MeOH for (*M*,*M,M*)/(*P*,*P*,*P*)-**1** and DFT transitions calculated for (*P*,*P*,*P*)-**1** in MeOH using B3LYP functional.

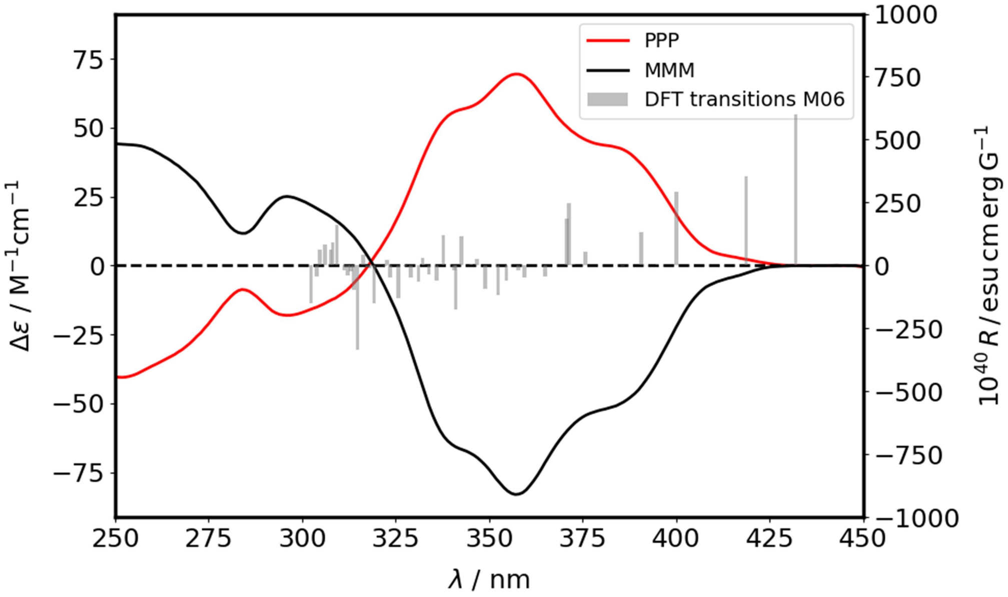

Figure S13: Experimental ECD in MeOH for (*M*,*M,M*)/(*P*,*P*,*P*)-**1** and DFT transitions calculated for (*P*,*P*,*P*)-**1** in MeOH using M06 functional.

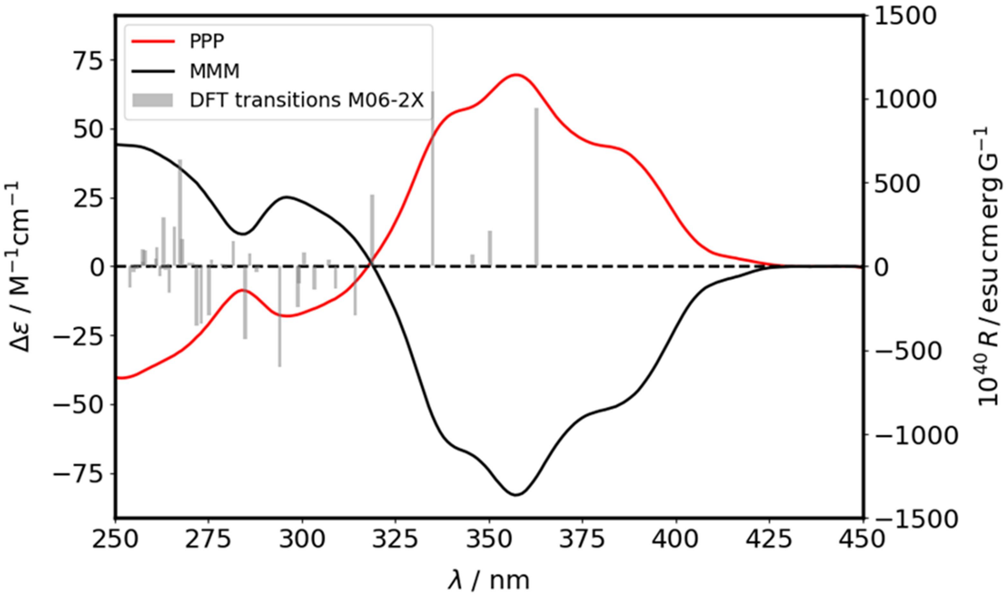

Figure S14: Experimental ECD in MeOH for (*M*,*M,M*)/(*P*,*P*,*P*)-**1** and DFT transitions calculated for (*P*,*P*,*P*)-**1** in MeOH using M06-2X functional.

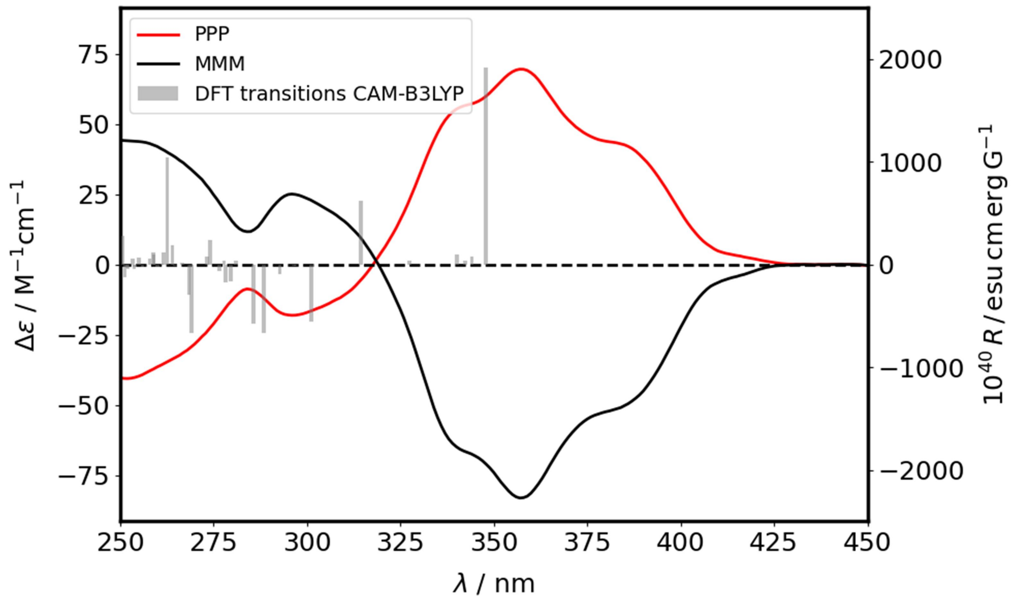

Figure S15: Experimental ECD in MeOH for (*M*,*M,M*)/(*P*,*P*,*P*)-**1** and DFT transitions calculated for (*P*,*P*,*P*)-**1** in MeOH using CAM-B3LYP functional.

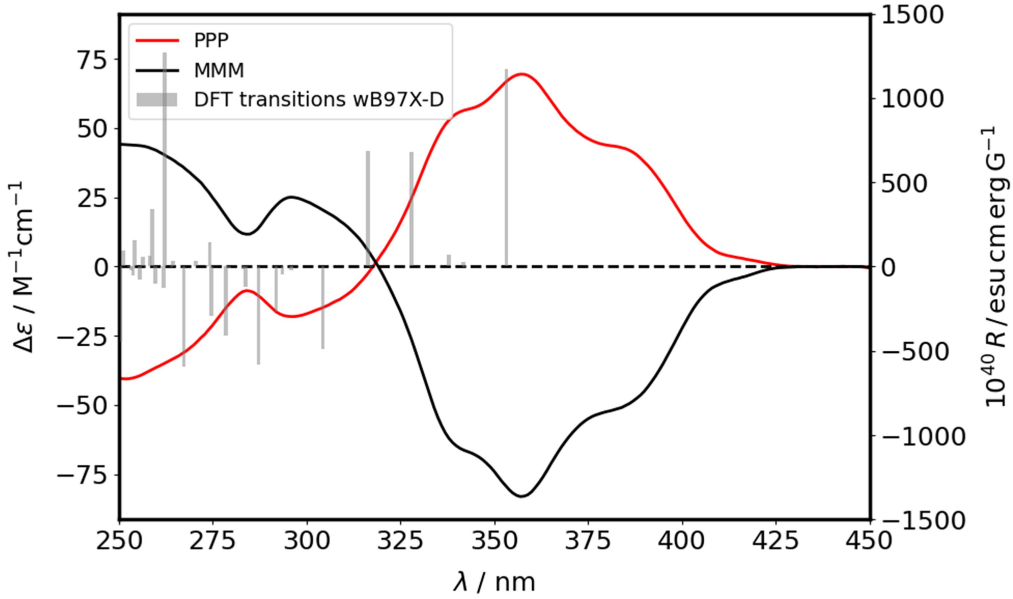

Figure S16: Experimental ECD in MeOH for (*M*,*M,M*)/(*P*,*P*,*P*)-**1** and DFT transitions calculated for (*P*,*P*,*P*)-**1** in MeOH using ωB97X-D functional.

Once the calculation conditions were selected, the ECD spectra of the considered conformers were computed, and the corresponding parameters were tabulated. The folded conformers exhibited remarkably high *g* values, primarily due to the high value of magnetic dipole transition moment and the optimal angle between this vector and the electric dipole transition moment (Table S5).

Table S5: TD-DFT study of (*P*,*P*,*P*)-**1** conformers for the $S_0 \rightarrow S_1$ transition.

Conformer	ΔG/kcal·mol^{-1}	Population/%	e·10^{18}/esu cm	m·10^{20}/erg G^{-1}	cos θ	g_{abs}
**I**	0.00	77.62	2.20	5.00	0.99	9.03E-02
**II**	1.00	14.24	2.27	4.90	0.98	8.54E-02
**III**	1.34	8.06	2.38	4.87	0.98	8.09E-02
**IV**	4.16	0.07	2.47	5.13	0.96	7.99E-02
**V**	9.50	0.00	2.72	4.04	0.76	4.51E-02
**VI**	4.97	0.02	2.64	4.70	0.39	2.83E-02
**VII**	8.78	0.00	2.20	4.39	0.70	5.62E-02
**VIII**	15.42	0.00	0.46	3.03	0.81	2.16E-01
**IX**	9.44	0.00	2.05	2.02	0.66	2.61E-02

The transition $S_0 \rightarrow S_1$ can be analysed in detail by examining the transition density (Figure S17), as well as the densities of the magnetic and electric dipole moments (Figure S18) using MultiWFN software.[14]

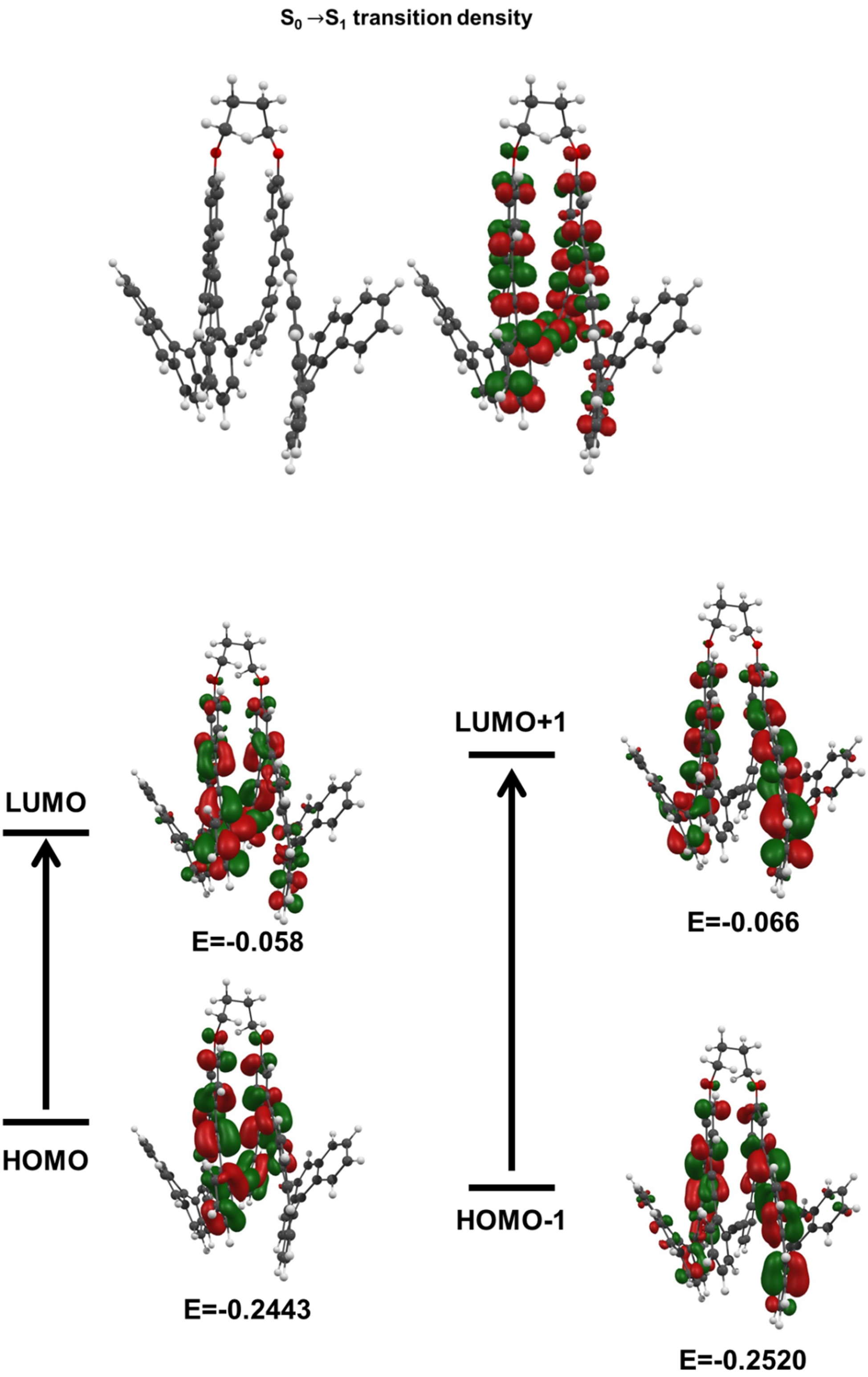

Figure S17: (Upper part) $S_0 \rightarrow S_1$ transition density. (Bottom part) MO involved in the transition $S_0 \rightarrow S_1$. Contour value=0.001.

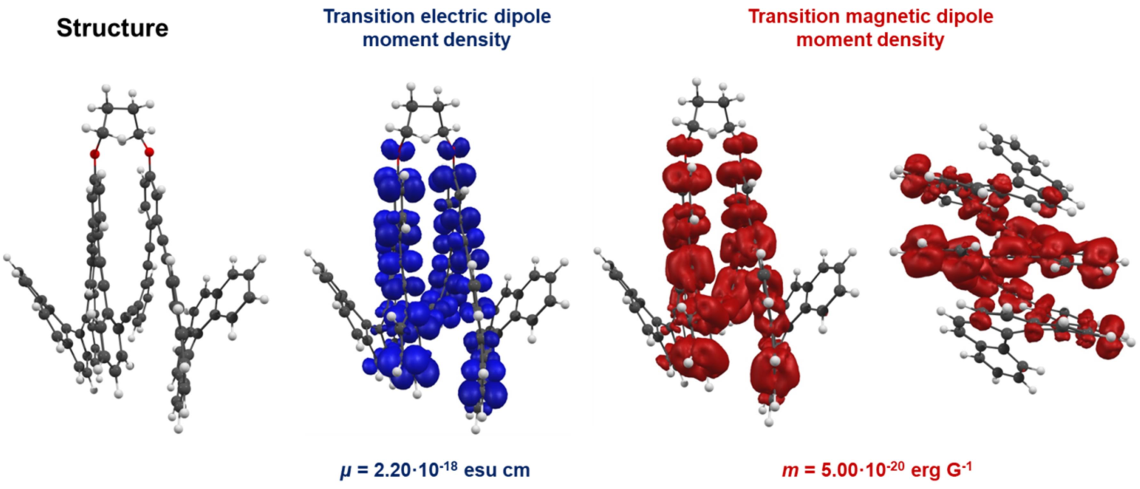

Figure S18: Electric and magnetic dipole transition densities for transition $S_0 \rightarrow S_1$. Contour value = 0.005.

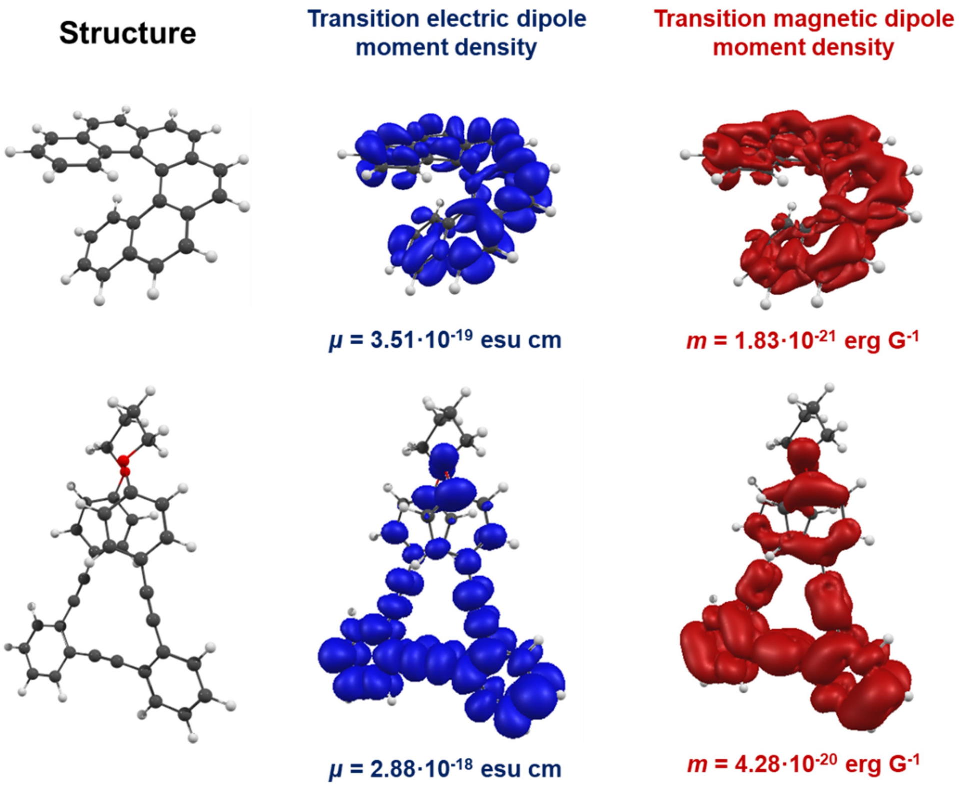

Figure S19: Electric and magnetic dipole transition densities of the [6]helicene and *o*-OPE components of compound **1** for transition $S_0 \rightarrow S_1$. Contour value = 0.005.

The $S_1 \rightarrow S_0$ transition was also analysed (Figure S20), and the relevant parameters are tabulated in Table S6. It can be observed that the theoretical *g* values for the emission process are significantly lower than their absorption counterparts, consistent with the experimental results, where the g_{lum} values are lower than the g_{abs} values.

Table S6: TD-DFT study of (*P*,*P*,*P*)-**1** conformers for the $S_1 \rightarrow S_0$ transition.

Conformer	Population/%	$e \cdot 10^{18}$/esu cm	$m \cdot 10^{20}$/erg G^{-1}	cos θ	g_{abs}
**I**	77.62	2.68	3.75	0.76	4.11E-02
**II**	14.24	3.03	5.28	0.99	6.93E-02
**III**	8.06	3.14	5.25	0.99	6.65E-02

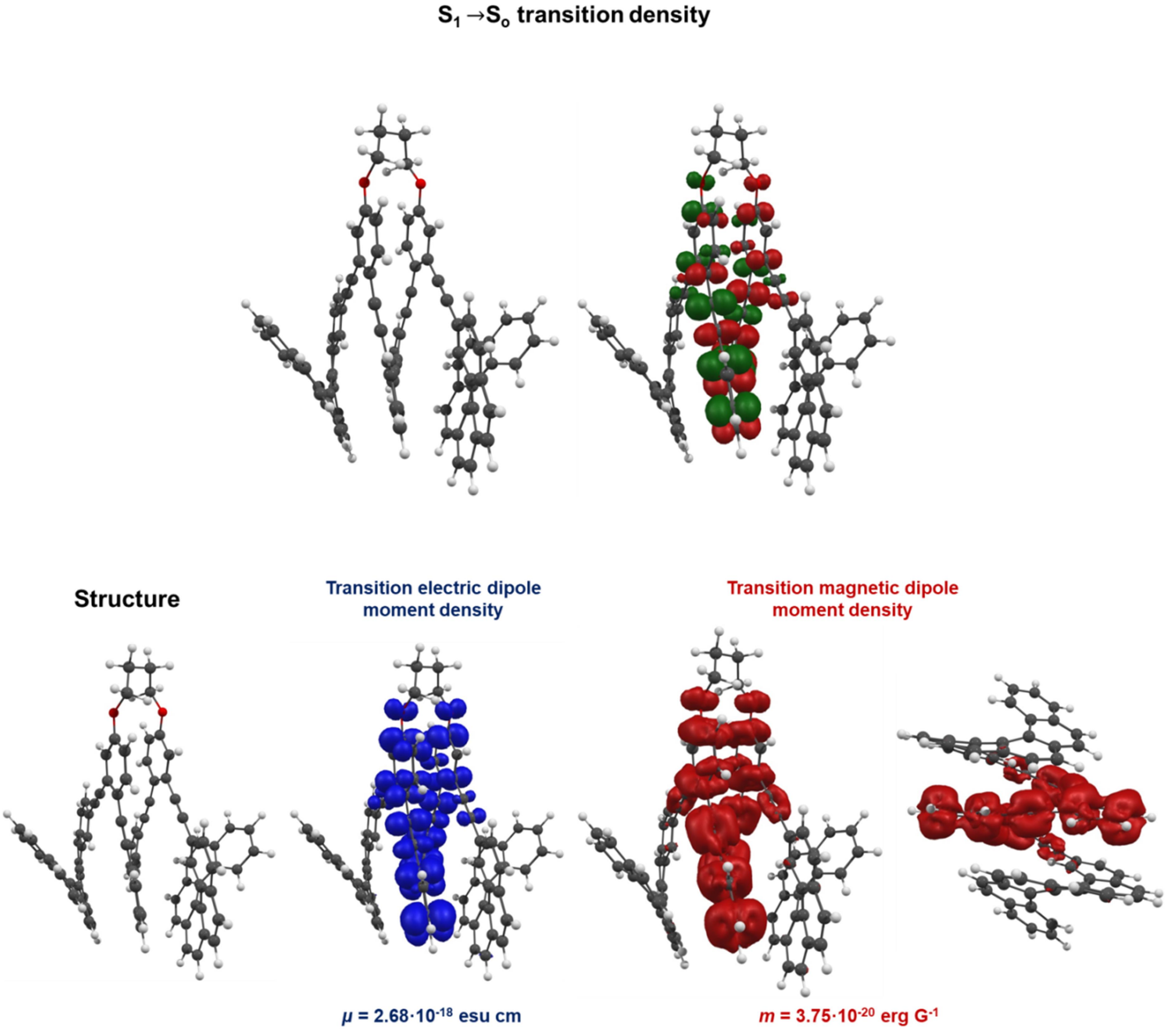

Figure S20: (Upper part) $S_1 \rightarrow S_0$ transition density. Contour value=0.001. (Bottom part) Electric and magnetic dipole transition densities for transition $S_1 \rightarrow S_0$. Contour value = 0.005.

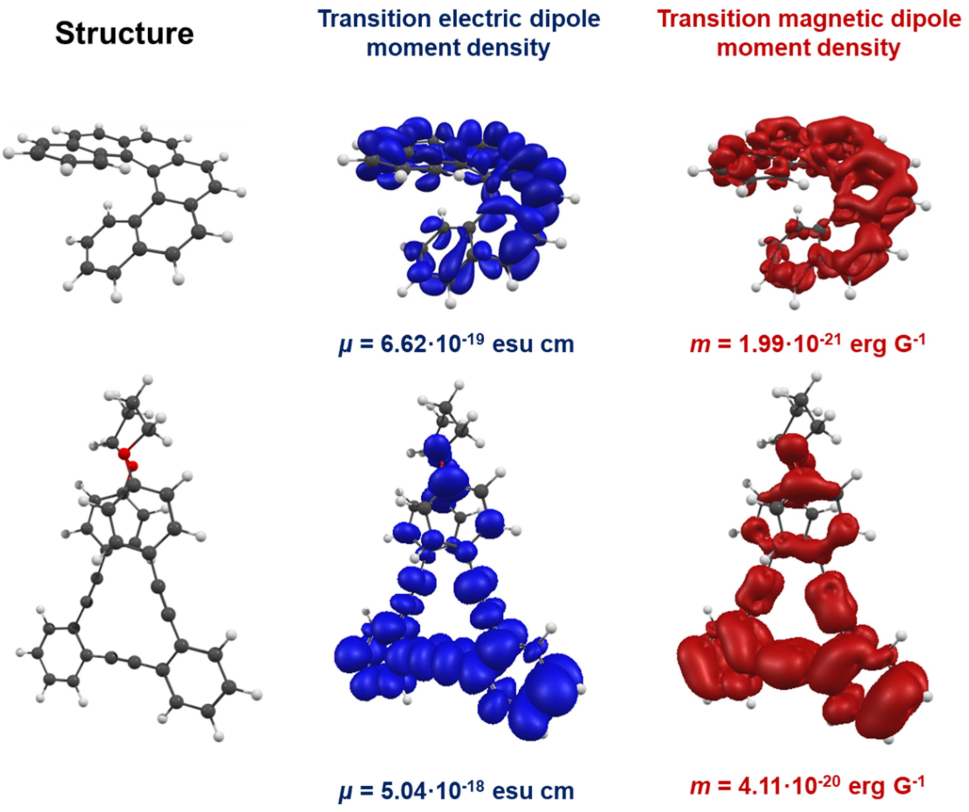

Figure S21: Electric and magnetic dipole transition densities of the [6]helicene and *o*-OPE components of compound **1** for transition $S_1 \rightarrow S_0$. Contour value = 0.005.

5.3. Effect of π-extension.

Compound **1** incorporates two [6]helicenes in its structure, the first configurationally stable member of the family of [n]helicenes. Some of us have recently reported that an increase in the internal area of a helix leads to a higher magnetic transition dipole moment, thereby enhancing chiroptical properties.[15] In this context, molecules analogous to compound **1** have been computationally studied, featuring a progressively increasing number of benzene rings from 1 to 6, to determine whether this trend also applies to this family of compounds—specifically, whether greater π-extension results in a higher magnetic dipole transition moment. The results confirm that our studies hold true for these structures.

This pattern continues with the next member of the series, incorporating [7]helicene. Moreover, and quite interestingly, in this molecule, the transition involving the entire helix ceases to be the first one, and it is observed that the $S_0 \rightarrow S_1$ transition becomes forbidden due to the predominance of the helicene over emissive *o*-OPE. (Figure S22) Consequently, the synthesized molecule incorporating a [6]helicene possesses a sufficient π-extension to be configurationally stable, yet not so extended as to lose its emissive properties. The same behavior can be observed when studying the emissive process (Figure S23). Additionally, for the emissive process, the magnetic dipole moment is lower and remains within a narrow range, which reduces the chiroptical properties and helps to explain why the g_{lum} factor is smaller than the observed g_{abs} factor.

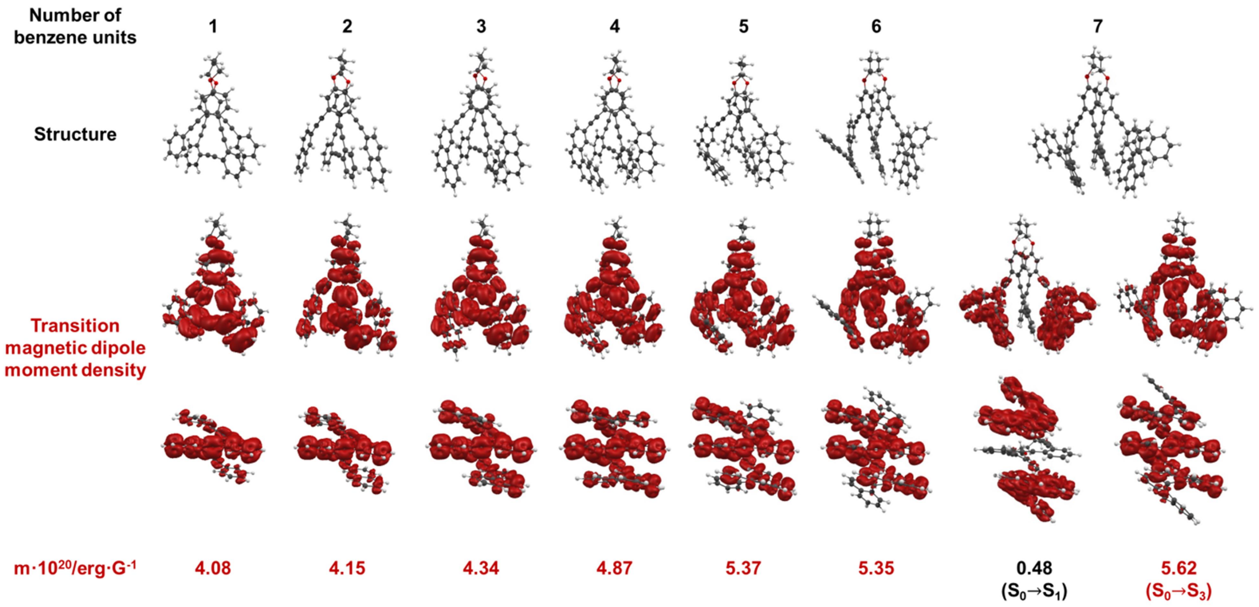

Figure S22: Effect of π-extension on magnetic transition dipole moment for the $S_0 \rightarrow S_1$ transition. Contour value = 0.005.

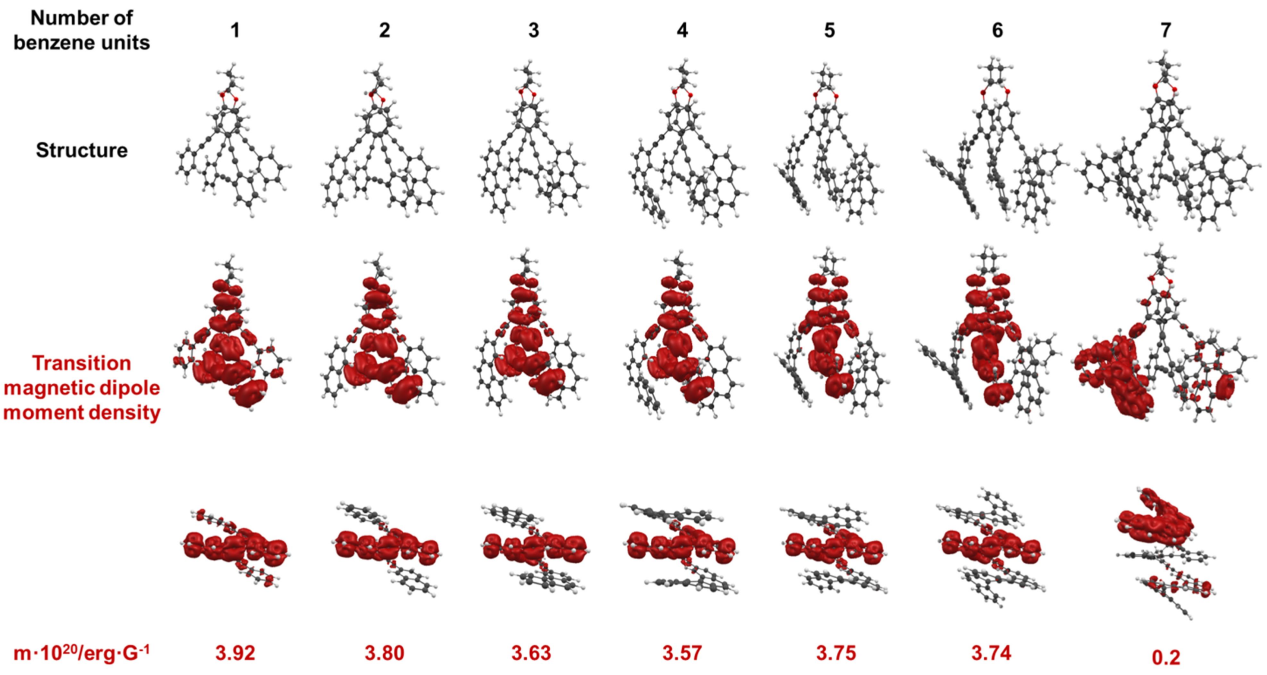

Figure S23: Effect of π-extension on magnetic transition dipole moment for the $S_1 \rightarrow S_0$ transition. Contour value = 0.005.

5.4. Cartesian coordinates of computed structures.

<u>(*P*)-[6]Helicene.</u>

C	0.000000000	3.829295000	-0.471886000
C	-0.584639000	2.729640000	-1.176361000
C	0.584639000	-2.729640000	-1.176361000
C	0.000000000	-3.829295000	-0.471886000
C	0.130927000	-1.283790000	0.787425000
C	0.711242000	-1.470597000	-0.535336000
C	0.000000000	0.000000000	1.449723000
C	-0.130927000	1.283790000	0.787425000
C	0.451102000	2.372691000	2.910215000
C	0.230884000	2.440659000	1.505000000
C	0.238601000	1.209320000	3.578837000
C	0.000000000	0.000000000	2.865697000
C	-0.230884000	-2.440659000	1.505000000

C	-0.332121000	-3.702364000	0.834654000
C	-1.101146000	2.910393000	-2.479380000
H	0.290246000	1.171174000	4.662951000
C	-0.451102000	-2.372691000	2.910215000
C	-0.238601000	-1.209320000	3.578837000
H	-0.290246000	-1.171174000	4.662951000
C	0.332121000	3.702364000	0.834654000
C	-0.711242000	1.470597000	-0.535336000
C	-1.783854000	1.902630000	-3.115515000
C	-1.998598000	0.685318000	-2.445847000
C	-1.478310000	0.478379000	-1.190299000
H	-0.104175000	-4.781886000	-0.983160000
H	-0.690384000	-4.556719000	1.401742000
H	-0.709233000	-3.283101000	3.442992000
H	0.709233000	3.283101000	3.442992000
H	0.690384000	4.556719000	1.401742000
H	0.104175000	4.781886000	-0.983160000
H	-0.969876000	3.875914000	-2.960113000
H	-2.182628000	2.054221000	-4.113338000
H	-2.584126000	-0.096916000	-2.917734000
H	-1.675151000	-0.459992000	-0.688641000
C	1.101146000	-2.910393000	-2.479380000
C	1.478310000	-0.478379000	-1.190299000
C	1.783854000	-1.902630000	-3.115515000
C	1.998598000	-0.685318000	-2.445847000
H	0.969876000	-3.875914000	-2.960113000
H	2.182628000	-2.054221000	-4.113338000
H	2.584126000	0.096916000	-2.917734000
H	1.675151000	0.459992000	-0.688641000

E = -1000.1305516 Hartrees.

(*P*)-*o*-OPE.

C	-2.359584000	-0.157048000	1.785370000
C	-1.036317000	-0.495546000	1.534323000
C	-0.698068000	-1.774116000	1.077673000
C	-1.717477000	-2.726909000	0.922604000
C	-3.037578000	-2.394411000	1.167689000
C	-3.369996000	-1.097250000	1.569160000
C	0.642547000	-2.106318000	0.714771000
C	1.753692000	-2.421024000	0.350731000
C	3.031556000	-2.887906000	-0.078956000
C	3.214936000	-4.260016000	-0.303618000
C	4.439817000	-4.757495000	-0.722035000
C	5.511141000	-3.889407000	-0.924351000
C	5.348345000	-2.529450000	-0.707522000
C	4.116432000	-2.008098000	-0.287144000
C	4.001629000	-0.600351000	-0.076310000
C	4.001599000	0.600414000	0.076230000
C	4.116348000	2.008155000	0.287133000
C	5.348253000	2.529519000	0.707525000
C	5.511029000	3.889471000	0.924380000
C	4.439700000	4.757552000	0.722050000
C	3.214836000	4.260067000	0.303597000
C	3.031464000	2.887954000	0.078940000
C	1.753620000	2.421092000	-0.350838000
C	0.642461000	2.106329000	-0.714794000
C	-0.698152000	1.774074000	-1.077652000
C	-1.036410000	0.495439000	-1.534123000
C	-2.359672000	0.156934000	-1.785173000
C	-3.370074000	1.097184000	-1.569135000
C	-3.037652000	2.394388000	-1.167811000

C	-1.717552000	2.726901000	-0.922737000
H	-2.598331000	0.845808000	2.119276000
H	-0.255554000	0.247444000	1.657570000
H	-1.463461000	-3.724261000	0.579678000
H	-3.836564000	-3.113178000	1.021287000
H	2.376510000	-4.928353000	-0.140678000
H	4.560046000	-5.822732000	-0.889422000
H	6.472054000	-4.272467000	-1.251323000
H	6.174661000	-1.844590000	-0.863781000
H	6.174577000	1.844665000	0.863770000
H	6.471937000	4.272537000	1.251360000
H	4.559920000	5.822790000	0.889437000
H	2.376411000	4.928397000	0.140627000
H	-0.255653000	-0.247576000	-1.657257000
H	-2.598437000	-0.845958000	-2.118967000
H	-3.836630000	3.113194000	-1.021547000
H	-1.463530000	3.724301000	-0.579952000
O	-4.696448000	0.830539000	-1.698240000
O	-4.696384000	-0.830604000	1.698149000
C	-5.131754000	0.496617000	1.411206000
H	-7.259159000	0.165268000	1.268397000
H	-5.274876000	1.045938000	2.349663000
C	-6.430782000	0.460194000	0.616148000
H	-4.363896000	1.015845000	0.831279000
H	-6.626702000	1.491579000	0.303857000
C	-6.430813000	-0.460187000	-0.616071000
H	-6.626738000	-1.491567000	-0.303767000
H	-7.259210000	-0.165252000	-1.268293000
C	-5.131821000	-0.496657000	-1.411190000
H	-5.275005000	-1.046028000	-2.349607000

H	-4.363926000	-1.015854000	-0.831281000

E = -1459.897276 Hartrees.

Transition state (TS) for the interconversion between (*P*)- and (*M*)-*o*-OPE enantiomers

C	-3.162701000	1.238731000	-1.031864000
C	-1.772633000	1.308131000	-1.053645000
C	-1.057324000	1.811116000	0.036311000
C	-1.770359000	2.203173000	1.181022000
C	-3.149645000	2.133054000	1.212633000
C	-3.858433000	1.677577000	0.095629000
C	0.355305000	2.038291000	-0.020021000
C	1.521024000	2.366523000	-0.031980000
C	2.810508000	2.988027000	-0.058682000
C	2.857790000	4.390446000	-0.075488000
C	4.067061000	5.068241000	-0.098813000
C	5.262973000	4.352926000	-0.106388000
C	5.236760000	2.967456000	-0.089176000
C	4.021454000	2.265124000	-0.063963000
C	4.071638000	0.839083000	-0.041827000
C	4.256813000	-0.356884000	-0.023603000
C	4.586042000	-1.745286000	-0.012882000
C	5.937161000	-2.120628000	-0.040914000
C	6.304812000	-3.457606000	-0.042224000
C	5.324092000	-4.447478000	-0.015504000
C	3.983257000	-4.093379000	0.015013000
C	3.592696000	-2.747079000	0.018288000
C	2.201434000	-2.425437000	0.050504000
C	1.005074000	-2.247498000	0.087603000
C	-0.419566000	-2.130554000	0.102149000
C	-1.095922000	-1.521287000	1.162806000
C	-2.485265000	-1.492080000	1.207121000

C	-3.219296000	-2.059769000	0.164995000
C	-2.553262000	-2.654538000	-0.911794000
C	-1.173079000	-2.694290000	-0.939827000
H	-3.685136000	0.846004000	-1.895432000
H	-1.234487000	0.976867000	-1.934731000
H	-1.226649000	2.587147000	2.037535000
H	-3.709598000	2.456117000	2.083418000
H	1.921302000	4.937338000	-0.071541000
H	4.077160000	6.153113000	-0.112125000
H	6.214012000	4.874813000	-0.125256000
H	6.160927000	2.399892000	-0.093611000
H	6.692091000	-1.342206000	-0.063920000
H	7.355221000	-3.728374000	-0.064883000
H	5.605038000	-5.495513000	-0.016749000
H	3.212622000	-4.856048000	0.039387000
H	-0.524743000	-1.074083000	1.968891000
H	-2.977721000	-1.025161000	2.050949000
H	-3.145718000	-3.085350000	-1.711427000
H	-0.659128000	-3.167150000	-1.769867000
O	-4.571602000	-2.100333000	0.093618000
O	-5.209658000	1.700683000	0.231689000
C	-6.055516000	1.389067000	-0.866591000
H	-7.008159000	1.856790000	-0.601696000
H	-5.687919000	1.877348000	-1.778324000
C	-6.271444000	-0.105059000	-1.098218000
H	-5.396996000	-0.563575000	-1.569172000
H	-7.091164000	-0.190242000	-1.820557000
C	-6.609821000	-0.902891000	0.167623000
H	-7.274147000	-0.328864000	0.824171000
H	-7.144228000	-1.816158000	-0.112836000

C	-5.368497000	-1.302078000	0.957056000
H	-5.636577000	-1.883438000	1.847977000
H	-4.824727000	-0.410919000	1.275267000

E = - 1459.38421 Hartrees.

Imaginary frequency = -8.4462 cm^{-1}.

Compound **1**. Conformer I.

C	3.148938000	2.042038000	3.391473000
C	2.963761000	1.384305000	2.185024000
C	1.707352000	0.882613000	1.823592000
C	0.619587000	1.043514000	2.708095000
C	0.821388000	1.725153000	3.915687000
C	2.071837000	2.220741000	4.256192000
C	1.574967000	0.245443000	0.553804000
C	-0.684759000	0.552052000	2.407974000
C	1.574962000	-0.244980000	-0.553298000
C	1.707131000	-0.882490000	-1.822939000
C	2.963278000	-1.385123000	-2.183968000
C	3.148319000	-2.043101000	-3.390301000
C	2.071322000	-2.221205000	-4.255270000
C	0.821102000	-1.724814000	-3.915095000
C	0.619463000	-1.042853000	-2.707660000
C	-0.684677000	-0.550617000	-2.407918000
C	-1.826382000	-0.192137000	-2.225840000
C	-1.826508000	0.193859000	2.225595000
C	-3.205398000	-0.106067000	2.019172000
C	-3.205299000	0.107694000	-2.019480000
C	-3.627307000	1.199189000	-1.222441000
C	-4.986888000	1.411635000	-1.008863000
C	-5.939387000	0.550172000	-1.550117000
C	-5.535093000	-0.514251000	-2.357897000

C	-4.179103000	-0.721978000	-2.579523000
C	-4.179264000	0.723498000	2.579263000
C	-5.535245000	0.515569000	2.357783000
C	-5.939479000	-0.548955000	1.550111000
C	-4.986911000	-1.410270000	1.008742000
C	-3.627334000	-1.197626000	1.222164000
O	-7.226728000	-0.817003000	1.219174000
O	-7.226619000	0.817963000	-1.218888000
H	4.126943000	2.440774000	3.639059000
H	3.788568000	1.263805000	1.491492000
H	-0.024293000	1.860333000	4.580813000
H	2.204605000	2.753954000	5.191748000
H	3.787932000	-1.265356000	-1.490112000
H	4.126107000	-2.442590000	-3.637543000
H	2.203974000	-2.754625000	-5.190723000
H	-0.024526000	-1.859585000	-4.580370000
H	-5.315725000	2.224935000	-0.373456000
H	-6.256964000	-1.186824000	-2.804521000
H	-6.257172000	1.188035000	2.804480000
H	-5.315702000	-2.223647000	0.373413000
C	0.948354000	-2.396590000	4.022198000
C	0.621871000	-3.620504000	3.356004000
C	1.029478000	-5.037636000	-2.025055000
C	2.018582000	-5.713257000	-2.801864000
C	2.825775000	-4.183819000	-0.536762000
C	1.422742000	-4.232139000	-0.925913000
C	3.325004000	-3.516951000	0.653856000
C	2.576870000	-3.284168000	1.873236000
C	4.245790000	-1.606926000	2.520288000
C	2.979178000	-2.225925000	2.709071000

C	5.091752000	-2.059050000	1.558723000
C	4.651375000	-3.020765000	0.606001000
C	3.763606000	-4.708405000	-1.447924000
C	3.334136000	-5.512350000	-2.550635000
C	-0.504956000	-4.375253000	3.749799000
H	6.100071000	-1.664777000	1.470976000
C	5.142715000	-4.373869000	-1.334081000
C	5.551025000	-3.477120000	-0.399373000
H	6.578502000	-3.125648000	-0.374041000
C	2.121186000	-1.772749000	3.762745000
C	1.471429000	-4.110923000	2.332568000
C	-0.752236000	-5.612512000	3.206082000
C	0.146621000	-6.148570000	2.267520000
C	1.229529000	-5.414923000	1.843350000
H	1.697710000	-6.337831000	-3.630544000
H	4.094173000	-5.956398000	-3.187392000
H	5.840961000	-4.780965000	-2.059796000
H	4.539619000	-0.811262000	3.197351000
H	2.414783000	-0.884436000	4.314896000
H	0.269014000	-2.012880000	4.777132000
H	-1.165003000	-3.964424000	4.508390000
H	-1.619681000	-6.184892000	3.518421000
H	-0.012296000	-7.147110000	1.873457000
H	1.913928000	-5.847442000	1.124091000
C	-0.336423000	-5.120495000	-2.384240000
C	0.429869000	-3.440494000	-0.312153000
C	-1.292376000	-4.407913000	-1.711352000
C	-0.902560000	-3.535177000	-0.668308000
H	-0.615066000	-5.757330000	-3.219081000
H	-2.339769000	-4.477809000	-1.984000000

H	0.696954000	-2.714660000	0.441777000
C	-2.675808000	-2.049690000	0.592712000
C	-1.870327000	-2.739488000	0.012043000
C	0.950781000	2.396235000	-4.023069000
C	0.624397000	3.620172000	-3.356877000
C	1.030604000	5.038173000	2.024308000
C	2.019814000	5.713467000	2.801269000
C	2.826950000	4.182799000	0.536985000
C	1.423820000	4.231935000	0.925686000
C	3.326222000	3.515456000	-0.653351000
C	2.578392000	3.282855000	-1.872962000
C	4.246874000	1.604840000	-2.519255000
C	2.980665000	2.224477000	-2.708646000
C	5.092667000	2.056667000	-1.557412000
C	4.652382000	3.018749000	-0.605020000
C	3.764754000	4.707210000	1.448278000
C	3.335344000	5.511712000	2.550605000
C	-0.501802000	4.375494000	-3.751384000
H	6.100757000	1.661914000	-1.469196000
C	5.143747000	4.372042000	1.334921000
C	5.551935000	3.474923000	0.400520000
H	6.579271000	3.123014000	0.375541000
C	2.123113000	1.771772000	-3.762876000
C	1.473426000	4.110023000	-2.332726000
C	-0.748994000	5.612731000	-3.207584000
C	0.149299000	6.148150000	-2.268118000
C	1.231584000	5.413964000	-1.843303000
H	1.698986000	6.338501000	3.629620000
H	4.095406000	5.955537000	3.187487000
H	5.841960000	4.779018000	2.060735000

H	4.540511000	0.808865000	-3.196039000
H	2.416679000	0.883368000	-4.314904000
H	0.271775000	2.012965000	-4.778529000
H	-1.161431000	3.965106000	-4.510578000
H	-1.615951000	6.185550000	-3.520477000
H	-0.009581000	7.146606000	-1.873828000
H	1.915426000	5.845877000	-1.123143000
C	-0.335372000	5.121962000	2.382981000
C	0.430742000	3.440531000	0.311930000
C	-1.291467000	4.409589000	1.710085000
C	-0.901755000	3.536115000	0.667616000
H	-0.613949000	5.759318000	3.217445000
H	-2.338921000	4.480175000	1.982319000
H	0.697729000	2.714066000	-0.441449000
C	-2.675737000	2.051256000	-0.593086000
C	-1.869849000	2.740726000	-0.012600000
H	-3.857923000	1.561982000	3.187643000
H	-3.857700000	-1.560411000	-3.187942000
C	-8.227315000	0.142292000	1.530492000
H	-7.879361000	1.147605000	1.264538000
H	-8.435526000	0.115365000	2.608651000
C	-9.473837000	-0.220887000	0.735103000
H	-10.342322000	0.226321000	1.230667000
H	-9.600995000	1.308225000	-0.801644000
H	-9.600973000	-1.307315000	0.801584000
H	-10.342204000	-0.225464000	-1.230789000
C	-9.473790000	0.221806000	-0.735152000
C	-8.227172000	-0.141295000	-1.530420000
H	-8.435240000	-0.114282000	-2.608604000
H	-7.879255000	-1.146634000	-1.264506000

E = -3610.0929503 Hartrees.

Compound **1**. Conformer II.

C	-3.156331000	2.230592000	3.281633000
C	-2.940269000	1.578412000	2.077122000
C	-1.704806000	0.984747000	1.792476000
C	-0.670291000	1.049279000	2.750596000
C	-0.901387000	1.724773000	3.955921000
C	-2.131326000	2.310125000	4.221295000
C	-1.529226000	0.353752000	0.525028000
C	0.609673000	0.467779000	2.514666000
C	-1.470528000	-0.129618000	-0.583608000
C	-1.544924000	-0.755039000	-1.863793000
C	-2.802159000	-1.164120000	-2.326329000
C	-2.938843000	-1.810855000	-3.545010000
C	-1.811644000	-2.073502000	-4.319774000
C	-0.559599000	-1.669388000	-3.879275000
C	-0.404229000	-0.998343000	-2.658431000
C	0.906127000	-0.592102000	-2.266449000
C	2.056392000	-0.301372000	-2.025166000
C	1.725716000	0.026175000	2.359383000
C	3.077045000	-0.387557000	2.170334000
C	3.436070000	-0.036365000	-1.775936000
C	3.856705000	1.041451000	-0.960131000
C	5.217068000	1.265341000	-0.760846000
C	6.176648000	0.444344000	-1.350525000
C	5.771753000	-0.635630000	-2.137718000
C	4.414429000	-0.858317000	-2.340960000
C	4.115021000	0.364462000	2.728106000
C	5.448574000	0.038897000	2.517700000
C	5.763631000	-1.067388000	1.725724000

C	4.746846000	-1.843628000	1.178495000
C	3.408695000	-1.516015000	1.384424000
O	7.025289000	-1.462759000	1.429014000
O	7.464389000	0.800615000	-1.100934000
H	-4.115303000	2.701049000	3.471088000
H	-3.722657000	1.532220000	1.327733000
H	-0.095409000	1.784629000	4.678871000
H	-2.287529000	2.837056000	5.156818000
H	-3.668828000	-0.979206000	-1.701269000
H	-3.920753000	-2.136410000	-3.871407000
H	-1.907599000	-2.599718000	-5.263758000
H	0.324030000	-1.868823000	-4.475352000
H	5.544353000	2.090652000	-0.140077000
H	6.490888000	-1.305218000	-2.593123000
H	6.227849000	0.647393000	2.961131000
H	5.010445000	-2.687445000	0.552587000
C	-1.466471000	-2.374335000	3.996165000
C	-1.224909000	-3.625718000	3.344754000
C	-1.387980000	-4.994577000	-2.049922000
C	-2.383006000	-5.562600000	-2.901899000
C	-3.189399000	-3.948003000	-0.697525000
C	-1.774746000	-4.146074000	-0.981579000
C	-3.700713000	-3.231644000	0.458071000
C	-3.022262000	-3.082002000	1.729976000
C	-4.548793000	-1.240773000	2.273112000
C	-3.369860000	-1.988790000	2.544996000
C	-5.364352000	-1.596235000	1.246814000
C	-4.958584000	-2.594902000	0.317026000
C	-4.106461000	-4.366526000	-1.681454000
C	-3.683834000	-5.215748000	-2.752803000

C	-0.216836000	-4.497133000	3.812673000
H	-6.316346000	-1.097008000	1.090914000
C	-5.444885000	-3.881397000	-1.669652000
C	-5.822864000	-2.947327000	-0.758775000
H	-6.804780000	-2.485490000	-0.807690000
C	-2.545566000	-1.629644000	3.660178000
C	-2.046084000	-4.022063000	2.259264000
C	-0.064824000	-5.753810000	3.278615000
C	-0.946071000	-6.190352000	2.274158000
C	-1.910866000	-5.344743000	1.779194000
H	-2.070118000	-6.222206000	-3.706138000
H	-4.436576000	-5.575483000	-3.448716000
H	-6.126226000	-4.207807000	-2.450152000
H	-4.807136000	-0.422321000	2.937260000
H	-2.782639000	-0.716238000	4.198340000
H	-0.806282000	-2.065721000	4.800906000
H	0.426361000	-4.159868000	4.620406000
H	0.711191000	-6.416164000	3.648356000
H	-0.867707000	-7.200569000	1.885629000
H	-2.585929000	-5.702889000	1.011930000
C	-0.015447000	-5.220039000	-2.307966000
C	-0.754571000	-3.451053000	-0.300309000
C	0.956735000	-4.594809000	-1.574251000
C	0.583169000	-3.672675000	-0.568161000
H	0.256647000	-5.890714000	-3.118091000
H	2.008491000	-4.767949000	-1.774206000
H	-1.001944000	-2.695530000	0.431196000
C	2.401233000	-2.297101000	0.750944000
C	1.574627000	-2.945386000	0.152716000
C	-0.397410000	2.469773000	-3.963810000

C	-0.045133000	3.665376000	-3.260199000
C	-0.784449000	5.063267000	2.086602000
C	-1.784754000	5.794583000	2.795704000
C	-2.511268000	4.348927000	0.449262000
C	-1.143064000	4.299359000	0.946762000
C	-2.958519000	3.727798000	-0.786079000
C	-2.133635000	3.454939000	-1.946128000
C	-3.853554000	1.899411000	-2.744690000
C	-2.537062000	2.431960000	-2.824160000
C	-4.740902000	2.399289000	-1.846069000
C	-4.314202000	3.320428000	-0.848090000
C	-3.480907000	4.925122000	1.293495000
C	-3.086836000	5.686017000	2.439223000
C	1.159473000	4.342844000	-3.550358000
H	-5.776936000	2.072979000	-1.843012000
C	-4.865867000	4.684169000	1.068400000
C	-5.257776000	3.826054000	0.091328000
H	-6.300916000	3.543861000	-0.018524000
C	-1.627623000	1.926858000	-3.809006000
C	-0.941861000	4.207774000	-2.305574000
C	1.443631000	5.557539000	-2.974575000
C	0.507529000	6.149591000	-2.108883000
C	-0.654372000	5.489708000	-1.784081000
H	-1.489556000	6.386430000	3.657286000
H	-3.863463000	6.173117000	3.022219000
H	-5.590490000	5.129225000	1.744234000
H	-4.145618000	1.131714000	-3.453976000
H	-1.933798000	1.063096000	-4.392483000
H	0.315001000	2.043539000	-4.663394000
H	1.851643000	3.891376000	-4.255294000

H	2.371157000	6.070569000	-3.207215000
H	0.700197000	7.133090000	-1.692699000
H	-1.366135000	5.964537000	-1.120108000
C	0.551981000	5.050412000	2.550809000
C	-0.159498000	3.451272000	0.397644000
C	1.509322000	4.286003000	1.939322000
C	1.145257000	3.454913000	0.854033000
H	0.805994000	5.656497000	3.415821000
H	2.535209000	4.284917000	2.291229000
H	-0.413932000	2.755063000	-0.387647000
C	2.907986000	1.898784000	-0.334338000
C	2.109396000	2.616149000	0.221909000
H	3.861600000	1.234236000	3.324396000
H	4.097080000	-1.696838000	-2.951376000
C	8.086054000	-0.527285000	1.547934000
H	7.748901000	0.462095000	1.223106000
H	8.418673000	-0.463097000	2.592851000
C	9.218583000	-1.024604000	0.658483000
H	10.043428000	-0.303605000	0.716761000
H	9.618692000	-1.784859000	-1.316775000
H	9.591799000	-1.968910000	1.068906000
H	7.935404000	-1.909946000	-0.822392000
C	8.810004000	-1.253348000	-0.801386000
C	8.535737000	0.014923000	-1.603865000
H	9.402319000	0.681206000	-1.556015000
H	8.364997000	-0.223974000	-2.661328000

E= -3610.0929615 Hartrees.

<u>Compound **1**. Conformer **III**.</u>

C	-2.966597000	-1.982877000	-3.481658000
C	-2.827625000	-1.352603000	-2.254377000

C	-1.589029000	-0.846546000	-1.842183000
C	-0.470001000	-0.978502000	-2.691958000
C	-0.625673000	-1.632280000	-3.921591000
C	-1.859998000	-2.129856000	-4.314655000
C	-1.492840000	-0.236484000	-0.557076000
C	0.817218000	-0.475441000	-2.337981000
C	-1.492866000	0.236482000	0.557142000
C	-1.589084000	0.846582000	1.842226000
C	-2.827759000	1.352390000	2.254488000
C	-2.966798000	1.982602000	3.481793000
C	-1.860191000	2.129761000	4.314752000
C	-0.625786000	1.632449000	3.921607000
C	-0.470050000	0.978719000	2.691954000
C	0.817206000	0.475714000	2.338027000
C	1.944135000	0.091750000	2.118858000
C	1.944148000	-0.091515000	-2.118752000
C	3.304123000	0.294842000	-1.926657000
C	3.304099000	-0.294600000	1.926676000
C	3.661703000	-1.432819000	1.166852000
C	5.002909000	-1.797226000	1.059109000
C	6.000138000	-1.063400000	1.694920000
C	5.661846000	0.088663000	2.409676000
C	4.326320000	0.454068000	2.518486000
C	4.326341000	-0.453820000	-2.518490000
C	5.661860000	-0.088391000	-2.409704000
C	6.000147000	1.063677000	-1.694939000
C	5.002922000	1.797469000	-1.059091000
C	3.661714000	1.433038000	-1.166829000
O	7.254819000	1.578356000	-1.587973000
O	7.254813000	-1.578054000	1.587891000

H	-3.931639000	-2.384663000	-3.771451000
H	-3.675727000	-1.256519000	-1.585394000
H	0.242177000	-1.744201000	-4.562038000
H	-1.957143000	-2.640591000	-5.267004000
H	-3.675881000	1.256125000	1.585556000
H	-3.931912000	2.384168000	3.771654000
H	-1.957393000	2.640454000	5.267117000
H	0.242070000	1.744496000	4.562024000
H	5.282800000	-2.673336000	0.486474000
H	6.419418000	0.693829000	2.893231000
H	6.419441000	-0.693541000	-2.893267000
H	5.282800000	2.673561000	-0.486422000
C	-0.890622000	2.524881000	-3.935753000
C	-0.616775000	3.746003000	-3.241017000
C	-1.126659000	5.052209000	2.140335000
C	-2.146503000	5.671689000	2.924481000
C	-2.880967000	4.147915000	0.631540000
C	-1.483547000	4.246014000	1.029725000
C	-3.344973000	3.487168000	-0.576127000
C	-2.577949000	3.310410000	-1.793037000
C	-4.179819000	1.592230000	-2.497316000
C	-2.932926000	2.257843000	-2.656832000
C	-5.048518000	1.986641000	-1.530584000
C	-4.650686000	2.937605000	-0.548859000
C	-3.843366000	4.612721000	1.549166000
C	-3.451392000	5.414774000	2.667612000
C	0.487633000	4.547991000	-3.603729000
H	-6.042165000	1.553339000	-1.461802000
C	-5.206071000	4.220918000	1.421972000
C	-5.572314000	3.331001000	0.463283000

H	-6.584052000	2.937672000	0.424317000
C	-2.045350000	1.856675000	-3.707393000
C	-1.497784000	4.185926000	-2.221304000
C	0.681792000	5.783011000	-3.033965000
C	-0.249802000	6.268909000	-2.100054000
C	-1.311166000	5.488703000	-1.705515000
H	-1.855658000	6.297046000	3.763579000
H	-4.231486000	5.813624000	3.310026000
H	-5.924067000	4.580517000	2.153457000
H	-4.440535000	0.806343000	-3.198789000
H	-2.300929000	0.971446000	-4.282887000
H	-0.187816000	2.180291000	-4.688075000
H	1.173029000	4.175634000	-4.359686000
H	1.532281000	6.392031000	-3.322713000
H	-0.133357000	7.265264000	-1.686114000
H	-2.021409000	5.882361000	-0.988908000
C	0.233774000	5.185783000	2.504559000
C	-0.460332000	3.496721000	0.414514000
C	1.218699000	4.515288000	1.829749000
C	0.865440000	3.634874000	0.780365000
H	0.484945000	5.825022000	3.346207000
H	2.261487000	4.623211000	2.107444000
H	-0.697752000	2.768860000	-0.347685000
C	2.676153000	2.214463000	-0.500813000
C	1.859768000	2.876884000	0.095908000
C	-0.890112000	-2.524704000	3.935459000
C	-0.616159000	-3.745807000	3.240725000
C	-1.126439000	-5.052277000	-2.140343000
C	-2.146259000	-5.671846000	-2.924457000
C	-2.880780000	-4.148176000	-0.631470000

C	-1.483374000	-4.246116000	-1.029732000
C	-3.344783000	-3.487512000	0.576244000
C	-2.577675000	-3.310654000	1.793085000
C	-4.179725000	-1.592714000	2.497525000
C	-2.932710000	-2.258128000	2.656904000
C	-5.048461000	-1.987240000	1.530870000
C	-4.650569000	-2.938113000	0.549081000
C	-3.843176000	-4.613063000	-1.549058000
C	-3.451163000	-5.415054000	-2.667538000
C	0.488504000	-4.547540000	3.603216000
H	-6.042182000	-1.554093000	1.462194000
C	-5.205915000	-4.221411000	-1.421772000
C	-5.572205000	-3.331576000	-0.463025000
H	-6.583985000	-2.938360000	-0.423992000
C	-2.045028000	-1.856754000	3.707304000
C	-1.497298000	-4.185967000	2.221231000
C	0.682780000	-5.782556000	3.033478000
C	-0.248947000	-6.268712000	2.099833000
C	-1.310558000	-5.488741000	1.705491000
H	-1.855385000	-6.297178000	-3.763564000
H	-4.231243000	-5.813955000	-3.309938000
H	-5.923909000	-4.581048000	-2.153241000
H	-4.440506000	-0.806897000	3.199055000
H	-2.300655000	-0.971520000	4.282770000
H	-0.187205000	-2.179913000	4.687594000
H	1.174013000	-4.174988000	4.358974000
H	1.533475000	-6.391375000	3.322042000
H	-0.132398000	-7.265068000	1.685926000
H	-2.020877000	-5.882565000	0.989046000
C	0.234006000	-5.185751000	-2.504572000

C	-0.460230000	-3.496694000	-0.414557000
C	1.218878000	-4.515198000	-1.829739000
C	0.865550000	-3.634785000	-0.780368000
H	0.485225000	-5.824961000	-3.346227000
H	2.261678000	-4.623035000	-2.107421000
H	-0.697730000	-2.768795000	0.347579000
C	2.676152000	-2.214269000	0.500850000
C	1.859829000	-2.876733000	-0.095910000
H	4.058066000	-1.341512000	-3.080893000
H	4.058046000	1.341737000	3.080926000
C	8.379680000	0.737201000	-1.795594000
H	8.344186000	0.271552000	-2.788638000
H	9.231003000	1.423257000	-1.786131000
C	8.519972000	-0.310658000	-0.698529000
H	7.694777000	-1.026026000	-0.767684000
H	9.443428000	0.877148000	0.870696000
H	9.443394000	-0.876890000	-0.870771000
H	7.694807000	1.026334000	0.767628000
C	8.519984000	0.310943000	0.698475000
C	8.379672000	-0.736909000	1.795551000
H	9.230995000	-1.422963000	1.786116000
H	8.344149000	-0.271251000	2.788586000

E= -3610.0897628 Hartrees.

Compound **1**. Conformer IV.

C	2.689299000	2.436707000	-3.465979000
C	2.649083000	1.765632000	-2.253369000
C	1.498899000	1.076533000	-1.849783000
C	0.371160000	1.058886000	-2.697764000
C	0.423752000	1.756896000	-3.911737000
C	1.569237000	2.440250000	-4.293855000

C	1.507265000	0.435827000	-0.575468000
C	-0.827049000	0.364783000	-2.358299000
C	1.615423000	-0.041750000	0.531782000
C	1.874965000	-0.639725000	1.800462000
C	3.202306000	-0.928867000	2.139827000
C	3.512862000	-1.539028000	3.345588000
C	2.494378000	-1.882662000	4.231329000
C	1.174148000	-1.599439000	3.911906000
C	0.843315000	-0.969052000	2.704996000
C	-0.528523000	-0.696480000	2.427951000
C	-1.716665000	-0.525803000	2.271914000
C	-1.889303000	-0.177213000	-2.150629000
C	-3.191477000	-0.722237000	-1.950029000
C	-3.133081000	-0.422233000	2.146430000
C	-3.749914000	0.600005000	1.399712000
C	-5.148388000	0.683988000	1.340140000
C	-5.939021000	-0.244210000	2.009788000
C	-5.329391000	-1.286191000	2.719790000
C	-3.952575000	-1.364767000	2.787238000
C	-4.289286000	-0.127214000	-2.578186000
C	-5.578743000	-0.618620000	-2.425639000
C	-5.797806000	-1.731229000	-1.607976000
C	-4.719299000	-2.329122000	-0.959134000
C	-3.421352000	-1.848413000	-1.125723000
O	-6.995203000	-2.337773000	-1.403262000
O	-7.296430000	-0.221326000	2.061889000
H	3.584155000	2.981671000	-3.747008000
H	3.504739000	1.779458000	-1.587472000
H	-0.453915000	1.754009000	-4.548517000
H	1.585884000	2.982650000	-5.233390000

H	3.983902000	-0.682729000	1.429634000
H	4.546546000	-1.773535000	3.576396000
H	2.728340000	-2.380557000	5.166496000
H	0.373460000	-1.865840000	4.592883000
H	-5.579195000	1.491237000	0.762688000
H	-5.960288000	-2.010723000	3.222014000
H	-6.393911000	-0.125823000	-2.941218000
H	-4.904590000	-3.182090000	-0.317847000
C	1.165530000	-2.320559000	-3.974822000
C	1.049122000	-3.571137000	-3.288548000
C	1.841208000	-4.846000000	2.083950000
C	2.945501000	-5.353170000	2.833292000
C	3.439247000	-3.750024000	0.528978000
C	2.073092000	-4.006334000	0.964716000
C	3.793085000	-3.029397000	-0.682425000
C	2.980003000	-2.927050000	-1.877939000
C	4.352967000	-1.023801000	-2.591606000
C	3.189962000	-1.830080000	-2.734152000
C	5.287674000	-1.330680000	-1.655085000
C	5.029118000	-2.336608000	-0.681642000
C	4.473980000	-4.113541000	1.413237000
C	4.206599000	-4.958482000	2.536415000
C	0.034638000	-4.490401000	-3.635274000
H	6.226582000	-0.786944000	-1.603551000
C	5.781775000	-3.574677000	1.251518000
C	6.019100000	-2.638394000	0.296867000
H	6.979714000	-2.135150000	0.234823000
C	2.239717000	-1.524965000	-3.761610000
C	1.998172000	-3.916651000	-2.293729000
C	-0.004544000	-5.744295000	-3.075056000

C	0.998352000	-6.129202000	-2.168264000
C	1.973298000	-5.236778000	-1.788889000
H	2.749709000	-6.007674000	3.677687000
H	5.045086000	-5.273460000	3.151245000
H	6.556264000	-3.861558000	1.957056000
H	4.501301000	-0.201479000	-3.284172000
H	2.378717000	-0.610617000	-4.331526000
H	0.411252000	-2.052453000	-4.708172000
H	-0.706601000	-4.191684000	-4.370924000
H	-0.787332000	-6.443398000	-3.350893000
H	1.005719000	-7.135950000	-1.763301000
H	2.740960000	-5.554317000	-1.094113000
C	0.515605000	-5.128433000	2.489902000
C	0.952863000	-3.384316000	0.376070000
C	-0.558257000	-4.578316000	1.842649000
C	-0.337838000	-3.673353000	0.777975000
H	0.363183000	-5.787782000	3.339690000
H	-1.573813000	-4.801985000	2.150686000
H	1.083582000	-2.640970000	-0.396581000
C	-2.343490000	-2.498035000	-0.460913000
C	-1.433348000	-3.046781000	0.115235000
C	0.610049000	2.497094000	3.977705000
C	0.071032000	3.644188000	3.312800000
C	0.123256000	5.054745000	-2.085402000
C	0.969165000	5.876429000	-2.889976000
C	2.067704000	4.530162000	-0.630761000
C	0.668403000	4.340059000	-0.988322000
C	2.696263000	3.967609000	0.552509000
C	2.023710000	3.625023000	1.789721000
C	3.960998000	2.253309000	2.407573000

C	2.613610000	2.655426000	2.621789000
C	4.699429000	2.831328000	1.424941000
C	4.085234000	3.698080000	0.477307000
C	2.885348000	5.193957000	-1.566533000
C	2.304594000	5.901858000	-2.665778000
C	-1.157515000	4.204098000	3.726925000
H	5.757172000	2.609277000	1.316685000
C	4.303096000	5.095498000	-1.481334000
C	4.874810000	4.288493000	-0.550576000
H	5.946779000	4.113248000	-0.546205000
C	1.865005000	2.075252000	3.696395000
C	0.806480000	4.261070000	2.269544000
C	-1.617675000	5.379088000	3.183564000
C	-0.839321000	6.050027000	2.224291000
C	0.341620000	5.503108000	1.780198000
H	0.531394000	6.428546000	-3.716593000
H	2.966239000	6.459569000	-3.322643000
H	4.908149000	5.606009000	-2.225110000
H	4.397239000	1.523541000	3.082067000
H	2.313978000	1.253280000	4.246825000
H	0.019597000	2.011879000	4.748778000
H	-1.724571000	3.695058000	4.501010000
H	-2.561371000	5.802187000	3.512386000
H	-1.169501000	7.005712000	1.830209000
H	0.930576000	6.038016000	1.045389000
C	-1.244864000	4.905039000	-2.413385000
C	-0.166437000	3.404494000	-0.342999000
C	-2.055545000	4.055462000	-1.709334000
C	-1.504494000	3.275302000	-0.665891000
H	-1.642895000	5.474898000	-3.248247000

H	-3.105948000	3.948527000	-1.957427000
H	0.233117000	2.744334000	0.412765000
C	-2.969648000	1.555071000	0.688780000
C	-2.311948000	2.349655000	0.057790000
H	-4.118850000	0.742018000	-3.204282000
H	-3.481820000	-2.166984000	3.345012000
C	-8.232585000	-1.692545000	-1.674726000
H	-8.167820000	-1.042237000	-2.554379000
H	-8.917756000	-2.506637000	-1.923982000
C	-8.740669000	-0.943184000	-0.448313000
H	-9.820184000	-0.781357000	-0.553800000
H	-7.003542000	0.349598000	-0.541382000
H	-8.598009000	-1.607777000	0.409873000
H	-8.522351000	1.204467000	-0.730599000
C	-8.033496000	0.391600000	-0.180835000
C	-8.008501000	0.744314000	1.301748000
H	-7.593678000	1.745910000	1.469181000
H	-9.019096000	0.729992000	1.719533000

E= -3610.0871178 Hartrees.

<u>Compound **1**. Conformer V.</u>

C	-0.281709000	-5.042180000	0.964533000
C	-1.083444000	-4.274479000	0.132406000
C	-1.015705000	-2.874797000	0.156494000
C	-0.118709000	-2.243151000	1.042462000
C	0.682386000	-3.033847000	1.876337000
C	0.606605000	-4.417540000	1.839440000
C	-1.839217000	-2.129149000	-0.737558000
C	0.033193000	-0.827024000	1.096317000
C	-2.543241000	-1.571977000	-1.549687000
C	-3.470672000	-0.980352000	-2.458956000

C	-4.634039000	-1.688212000	-2.785212000
C	-5.605395000	-1.122871000	-3.597073000
C	-5.430754000	0.166433000	-4.091847000
C	-4.274696000	0.873384000	-3.794481000
C	-3.274541000	0.313965000	-2.988234000
C	-2.106566000	1.085786000	-2.710935000
C	-1.163486000	1.823789000	-2.533265000
C	0.278125000	0.354504000	1.189749000
C	0.717575000	1.707570000	1.290023000
C	-0.158490000	2.812329000	-2.312459000
C	1.188239000	2.488125000	-2.021974000
C	2.105606000	3.509140000	-1.784716000
C	1.712456000	4.845492000	-1.799871000
C	0.392370000	5.177521000	-2.106677000
C	-0.521325000	4.161117000	-2.357171000
C	2.073470000	1.956907000	1.506727000
C	2.577790000	3.248888000	1.577221000
C	1.708567000	4.329369000	1.416145000
C	0.347756000	4.103412000	1.213443000
C	-0.157257000	2.808018000	1.132926000
O	2.089920000	5.630662000	1.408379000
O	2.679883000	5.741084000	-1.483318000
H	-0.345677000	-6.124961000	0.928779000
H	-1.782431000	-4.745362000	-0.550179000
H	1.375523000	-2.533722000	2.541700000
H	1.242517000	-5.010962000	2.488675000
H	-4.781495000	-2.674230000	-2.358003000
H	-6.510739000	-1.678711000	-3.815524000
H	-6.198571000	0.623620000	-4.707107000
H	-4.128684000	1.876825000	-4.178413000

H	3.130768000	3.266466000	-1.532712000
H	0.066247000	6.209851000	-2.142603000
H	3.640856000	3.395012000	1.726263000
H	-0.307753000	4.952760000	1.064353000
C	-2.672352000	-1.416911000	3.595511000
C	-3.691279000	-0.415700000	3.508028000
C	-6.579437000	1.492975000	-0.853097000
C	-7.848968000	1.169797000	-1.420902000
C	-6.348864000	-0.863874000	-0.105840000
C	-5.797585000	0.476833000	-0.246746000
C	-5.718161000	-1.936222000	0.644151000
C	-4.811685000	-1.770235000	1.763104000
C	-4.103480000	-4.100834000	1.473440000
C	-3.918733000	-2.817240000	2.057793000
C	-5.166023000	-4.336164000	0.660605000
C	-5.995004000	-3.262370000	0.228988000
C	-7.508760000	-1.163321000	-0.847135000
C	-8.267733000	-0.117570000	-1.461411000
C	-3.631494000	0.743567000	4.312549000
H	-5.377714000	-5.335985000	0.292554000
C	-7.895675000	-2.516284000	-1.062162000
C	-7.104144000	-3.529224000	-0.623816000
H	-7.327061000	-4.561625000	-0.877263000
C	-2.826902000	-2.601727000	2.959344000
C	-4.804341000	-0.620259000	2.654275000
C	-4.670468000	1.642108000	4.335880000
C	-5.823658000	1.386684000	3.573560000
C	-5.886643000	0.283537000	2.755204000
H	-8.439110000	1.963035000	-1.870641000
H	-9.193247000	-0.383856000	-1.964142000

H	-8.785057000	-2.718229000	-1.652149000
H	-3.414736000	-4.895899000	1.741306000
H	-2.100432000	-3.399971000	3.082368000
H	-1.809191000	-1.232836000	4.227807000
H	-2.752178000	0.901269000	4.930491000
H	-4.614988000	2.527973000	4.960188000
H	-6.671172000	2.062102000	3.629370000
H	-6.787110000	0.100169000	2.182370000
C	-6.072816000	2.810556000	-0.943805000
C	-4.477653000	0.805515000	0.125496000
C	-4.820875000	3.122871000	-0.485663000
C	-3.997588000	2.099698000	0.040425000
H	-6.696787000	3.576148000	-1.396216000
H	-4.437997000	4.135756000	-0.547245000
H	-3.799833000	0.038225000	0.471505000
C	-1.530402000	2.596024000	0.820233000
C	-2.667781000	2.384676000	0.468240000
C	6.046704000	1.272317000	1.832621000
C	5.297176000	0.085702000	2.110648000
C	3.478403000	-3.949158000	-1.442273000
C	3.988071000	-5.279073000	-1.571794000
C	5.790682000	-3.157774000	-1.025405000
C	4.372178000	-2.875145000	-1.196691000
C	6.767867000	-2.172738000	-0.615502000
C	6.514413000	-1.060450000	0.277080000
C	8.592377000	-0.030576000	-0.526996000
C	7.376118000	0.050901000	0.211398000
C	8.979642000	-1.212567000	-1.075016000
C	8.083104000	-2.319194000	-1.113013000
C	6.255060000	-4.425753000	-1.421372000

C	5.325024000	-5.495831000	-1.624690000
C	4.384286000	0.059438000	3.189887000
H	9.961147000	-1.322857000	-1.526556000
C	7.641236000	-4.624923000	-1.684841000
C	8.502620000	-3.573777000	-1.642266000
H	9.534066000	-3.684080000	-1.963597000
C	7.076346000	1.238426000	0.953298000
C	5.530776000	-1.090454000	1.351206000
C	3.750999000	-1.103574000	3.553945000
C	4.036090000	-2.288584000	2.853668000
C	4.893538000	-2.279611000	1.779378000
H	3.285884000	-6.094507000	-1.718581000
H	5.718733000	-6.486597000	-1.832584000
H	7.975064000	-5.605762000	-2.010257000
H	9.244393000	0.837309000	-0.556797000
H	7.710030000	2.108501000	0.807366000
H	5.828849000	2.172328000	2.401223000
H	4.204707000	0.978700000	3.740557000
H	3.060156000	-1.113482000	4.390776000
H	3.589191000	-3.226367000	3.168510000
H	5.111172000	-3.213358000	1.279016000
C	2.108594000	-3.683816000	-1.669565000
C	3.865054000	-1.560830000	-1.280382000
C	1.641016000	-2.399568000	-1.754042000
C	2.535005000	-1.316808000	-1.580464000
H	1.430711000	-4.518580000	-1.820864000
H	0.600778000	-2.191436000	-1.976290000
H	4.530999000	-0.714977000	-1.167839000
C	1.630960000	1.137434000	-1.912751000
C	2.061861000	0.018743000	-1.751363000

H	2.745131000	1.111828000	1.593537000
H	-1.553720000	4.410380000	-2.576692000
C	3.475735000	5.943141000	1.387625000
H	3.993555000	5.312906000	0.655101000
H	3.906089000	5.756244000	2.380942000
C	3.602669000	7.414743000	1.015600000
H	4.571462000	7.780554000	1.372548000
H	4.398915000	7.426951000	-1.005173000
H	2.836348000	7.960273000	1.577618000
H	3.408276000	8.819926000	-0.600508000
C	3.485862000	7.733705000	-0.482697000
C	2.311695000	7.084537000	-1.203435000
H	2.097442000	7.608622000	-2.144540000
H	1.412937000	7.104887000	-0.575839000

E= -3610.0755814 Hartrees.

Compound **1**. Conformer VI.

C	7.851768000	3.133143000	-0.245852000
C	7.038782000	2.198515000	-0.869255000
C	5.642553000	2.258772000	-0.751358000
C	5.063226000	3.295593000	0.011520000
C	5.900409000	4.236248000	0.629794000
C	7.279651000	4.157115000	0.508021000
C	4.865368000	1.244966000	-1.387322000
C	3.652926000	3.416735000	0.189332000
C	4.319349000	0.330493000	-1.963192000
C	3.742999000	-0.819945000	-2.582525000
C	4.516130000	-1.981603000	-2.700445000
C	3.969601000	-3.148176000	-3.214258000
C	2.636372000	-3.173587000	-3.614953000
C	1.861766000	-2.026146000	-3.523314000

C	2.399825000	-0.835599000	-3.018718000
C	1.563983000	0.315069000	-2.925026000
C	0.785472000	1.237947000	-2.849011000
C	2.477389000	3.613840000	0.402332000
C	1.099200000	3.916322000	0.610802000
C	-0.186176000	2.271377000	-2.719942000
C	-1.546438000	1.945376000	-2.555375000
C	-2.509660000	2.956758000	-2.433087000
C	-2.125880000	4.292226000	-2.449520000
C	-0.770821000	4.621373000	-2.579255000
C	0.177920000	3.626144000	-2.715880000
C	0.693418000	5.245497000	0.764108000
C	-0.645110000	5.588292000	0.908485000
C	-1.610193000	4.580561000	0.881888000
C	-1.228691000	3.251111000	0.737560000
C	0.113612000	2.901368000	0.620200000
O	-2.943528000	4.797602000	0.996873000
O	-2.978308000	5.344083000	-2.332795000
H	8.929888000	3.065461000	-0.348986000
H	7.471384000	1.391244000	-1.449748000
H	5.444835000	5.027124000	1.215749000
H	7.908153000	4.893264000	0.998342000
H	5.540842000	-1.965712000	-2.344890000
H	4.578142000	-4.044055000	-3.271396000
H	2.198813000	-4.091074000	-3.994268000
H	0.823319000	-2.029351000	-3.836189000
H	-3.541288000	2.660443000	-2.296212000
H	-0.486345000	5.667104000	-2.565253000
H	-0.927313000	6.627017000	1.035074000
H	-1.990947000	2.485402000	0.669691000

C	4.607988000	1.230451000	3.009943000
C	3.672118000	0.172064000	3.240030000
C	1.677835000	-3.724430000	-0.139933000
C	2.036053000	-5.074483000	-0.437675000
C	4.066500000	-3.232402000	0.324433000
C	2.690757000	-2.784934000	0.177406000
C	5.144382000	-2.399130000	0.824673000
C	4.992887000	-1.266695000	1.716670000
C	7.243125000	-0.528693000	1.078670000
C	5.992630000	-0.276755000	1.708846000
C	7.495052000	-1.739238000	0.515178000
C	6.452949000	-2.698911000	0.372981000
C	4.382927000	-4.514075000	-0.164916000
C	3.340853000	-5.435266000	-0.502791000
C	2.555366000	0.369015000	4.081838000
H	8.479404000	-1.979100000	0.123457000
C	5.739716000	-4.874921000	-0.401742000
C	6.728613000	-3.958413000	-0.233167000
H	7.752471000	-4.184364000	-0.516792000
C	5.760217000	0.986780000	2.342531000
C	3.901560000	-1.103664000	2.665824000
C	1.728199000	-0.676705000	4.415334000
C	2.015940000	-1.967194000	3.935621000
C	3.076457000	-2.172533000	3.084202000
H	1.248531000	-5.785525000	-0.670514000
H	3.623710000	-6.438038000	-0.810766000
H	5.956991000	-5.863240000	-0.796664000
H	8.005863000	0.242857000	1.114768000
H	6.512999000	1.761786000	2.229109000
H	4.404641000	2.210878000	3.429613000

H	2.374372000	1.363340000	4.479768000
H	0.876173000	-0.515640000	5.068447000
H	1.400131000	-2.807802000	4.239238000
H	3.285211000	-3.174135000	2.729326000
C	0.330268000	-3.302059000	-0.220838000
C	2.320003000	-1.428619000	0.262677000
C	-0.014985000	-1.988963000	-0.039503000
C	0.999474000	-1.030690000	0.187249000
H	-0.438934000	-4.035024000	-0.447167000
H	-1.048148000	-1.669868000	-0.089477000
H	3.073023000	-0.661668000	0.371757000
C	0.454095000	1.528275000	0.479526000
C	0.682218000	0.349457000	0.340888000
C	-4.361744000	1.658567000	1.939307000
C	-3.688763000	0.396042000	2.007735000
C	-4.248625000	-4.196014000	-1.227564000
C	-4.954048000	-5.418736000	-1.003862000
C	-6.006615000	-2.994122000	0.052590000
C	-4.791764000	-2.976479000	-0.748755000
C	-6.544534000	-1.835421000	0.738252000
C	-5.775044000	-0.711810000	1.241702000
C	-7.862073000	0.578628000	1.353608000
C	-6.439851000	0.518955000	1.409211000
C	-8.590243000	-0.561099000	1.224665000
C	-7.947422000	-1.795599000	0.922483000
C	-6.762209000	-4.182151000	0.048654000
C	-6.187204000	-5.398875000	-0.441995000
C	-2.346054000	0.326067000	2.445949000
H	-9.673218000	-0.544386000	1.303676000
C	-8.120102000	-4.165991000	0.477384000

C	-8.716389000	-2.988540000	0.800604000
H	-9.780794000	-2.942630000	1.011341000
C	-5.694425000	1.706498000	1.700645000
C	-4.393557000	-0.797131000	1.697555000
C	-1.718752000	-0.882477000	2.621059000
C	-2.436579000	-2.069101000	2.391708000
C	-3.736564000	-2.026443000	1.944680000
H	-4.517296000	-6.348674000	-1.355855000
H	-6.768307000	-6.312113000	-0.351939000
H	-8.687731000	-5.091433000	0.454421000
H	-8.349709000	1.536770000	1.507802000
H	-6.227591000	2.652723000	1.735533000
H	-3.806228000	2.569848000	2.140624000
H	-1.815629000	1.249553000	2.656927000
H	-0.680434000	-0.920117000	2.934660000
H	-1.958961000	-3.028440000	2.562925000
H	-4.271783000	-2.955942000	1.801070000
C	-3.051755000	-4.179620000	-1.981892000
C	-4.172828000	-1.779640000	-1.158027000
C	-2.432389000	-3.002099000	-2.313543000
C	-3.016070000	-1.775400000	-1.918739000
H	-2.637450000	-5.125860000	-2.318749000
H	-1.508171000	-2.995276000	-2.880257000
H	-4.605334000	-0.826672000	-0.886805000
C	-1.980382000	0.592775000	-2.428636000
C	-2.427576000	-0.517529000	-2.250339000
H	1.449002000	6.023841000	0.762341000
H	1.227177000	3.884378000	-2.805284000
C	-3.478469000	6.045685000	0.576161000
H	-2.869122000	6.453246000	-0.235902000

H	-3.477941000	6.755290000	1.413774000
C	-4.898043000	5.780497000	0.094020000
H	-5.326677000	6.728599000	-0.254084000
H	-6.041934000	4.488517000	-1.190847000
H	-5.501469000	5.453690000	0.948272000
H	-4.527422000	3.793287000	-0.638502000
C	-4.985576000	4.716543000	-1.005075000
C	-4.384247000	5.120391000	-2.349953000
H	-4.806036000	6.078211000	-2.670228000
H	-4.630495000	4.378250000	-3.119340000

E = - -3610.085657 Hartrees.

<u>Compound **1**. Conformer VII.</u>

C	3.986097000	-2.632602000	-3.445361000
C	4.460087000	-1.451406000	-2.893841000
C	3.600601000	-0.366983000	-2.682239000
C	2.246979000	-0.471696000	-3.070019000
C	1.779879000	-1.679538000	-3.602718000
C	2.640503000	-2.752963000	-3.782733000
C	4.096247000	0.793305000	-2.014074000
C	1.335796000	0.610593000	-2.905658000
C	4.560322000	1.721365000	-1.390177000
C	5.249361000	2.760943000	-0.697453000
C	6.641909000	2.845483000	-0.843185000
C	7.377260000	3.806061000	-0.165455000
C	6.728314000	4.711178000	0.673477000
C	5.351067000	4.647531000	0.822992000
C	4.591520000	3.679106000	0.149261000
C	3.179138000	3.662658000	0.355713000
C	1.997270000	3.759253000	0.602158000
C	0.501617000	1.478723000	-2.790455000

C	-0.561223000	2.404917000	-2.592903000
C	0.608831000	3.979142000	0.848958000
C	-0.332046000	2.920527000	0.837720000
C	-1.687733000	3.202007000	0.987824000
C	-2.130176000	4.514495000	1.144209000
C	-1.205627000	5.557838000	1.219146000
C	0.146677000	5.278797000	1.064391000
C	-0.333688000	3.782455000	-2.455711000
C	-1.373654000	4.653609000	-2.195145000
C	-2.679205000	4.168666000	-2.053009000
C	-2.935508000	2.812234000	-2.216778000
C	-1.880734000	1.928517000	-2.478551000
O	-3.625196000	5.086150000	-1.725078000
O	-3.477055000	4.681451000	1.198243000
H	4.662545000	-3.470205000	-3.575907000
H	5.494827000	-1.368052000	-2.579988000
H	0.728780000	-1.759618000	-3.857344000
H	2.260265000	-3.687681000	-4.181031000
H	7.134774000	2.128668000	-1.490570000
H	8.454103000	3.850883000	-0.291840000
H	7.295121000	5.466449000	1.207929000
H	4.836379000	5.346066000	1.473803000
H	-2.413307000	2.398821000	0.938934000
H	-1.521257000	6.580998000	1.379691000
H	-1.193528000	5.712671000	-2.051865000
H	-3.931373000	2.398910000	-2.118713000
C	-4.924959000	1.331334000	1.252645000
C	-5.554140000	0.444309000	0.322518000
C	-3.766390000	-4.560688000	-1.617812000
C	-4.068902000	-5.908294000	-1.250540000

C	-4.176959000	-3.863749000	0.739121000
C	-3.787219000	-3.544123000	-0.627946000
C	-4.332509000	-2.879513000	1.796798000
C	-4.675070000	-1.479239000	1.622158000
C	-3.810404000	-1.042873000	3.878271000
C	-4.270875000	-0.568611000	2.617512000
C	-3.829051000	-2.373039000	4.152633000
C	-4.091128000	-3.319352000	3.120939000
C	-4.260552000	-5.228785000	1.076287000
C	-4.260570000	-6.229995000	0.051422000
C	-6.296117000	0.954225000	-0.766321000
H	-3.590628000	-2.741639000	5.145852000
C	-4.272654000	-5.631865000	2.441980000
C	-4.091146000	-4.710994000	3.423429000
H	-3.972641000	-5.016061000	4.458892000
C	-4.360624000	0.841709000	2.382570000
C	-5.483412000	-0.957923000	0.528952000
C	-6.991092000	0.117761000	-1.605798000
C	-6.993305000	-1.265077000	-1.354651000
C	-6.262399000	-1.785507000	-0.312513000
H	-4.078735000	-6.673623000	-2.021031000
H	-4.409095000	-7.264144000	0.348562000
H	-4.334090000	-6.690715000	2.674735000
H	-3.522944000	-0.319626000	4.635254000
H	-3.948923000	1.515609000	3.128256000
H	-4.947994000	2.402839000	1.072934000
H	-6.331717000	2.030507000	-0.911243000
H	-7.559187000	0.522045000	-2.437217000
H	-7.580361000	-1.928317000	-1.981304000
H	-6.294569000	-2.851578000	-0.126452000

C	-3.381106000	-4.242024000	-2.941534000
C	-3.326861000	-2.270276000	-1.005153000
C	-2.978323000	-2.976614000	-3.288572000
C	-2.931352000	-1.973003000	-2.296628000
H	-3.388944000	-5.032849000	-3.686241000
H	-2.679716000	-2.743816000	-4.304730000
H	-3.257159000	-1.480477000	-0.273153000
C	-2.173735000	0.532363000	-2.545949000
C	-2.494235000	-0.634336000	-2.543868000
C	4.504653000	1.409964000	3.008342000
C	3.644179000	0.284455000	3.210531000
C	1.703203000	-3.519320000	-0.318916000
C	2.127759000	-4.824912000	-0.711819000
C	4.077571000	-2.900619000	0.057977000
C	2.671124000	-2.532781000	0.001305000
C	5.128425000	-2.031758000	0.556191000
C	4.957087000	-0.966154000	1.523564000
C	7.123846000	-0.053310000	0.825554000
C	5.896463000	0.081500000	1.532500000
C	7.417627000	-1.211321000	0.177898000
C	6.428943000	-2.225065000	0.029500000
C	4.446265000	-4.133549000	-0.513545000
C	3.447106000	-5.098831000	-0.856301000
C	2.567296000	0.356035000	4.121662000
H	8.392988000	-1.366632000	-0.274398000
C	5.809419000	-4.397516000	-0.829229000
C	6.749346000	-3.431680000	-0.656930000
H	7.769954000	-3.578961000	-0.998373000
C	5.628428000	1.283136000	2.264430000
C	3.911293000	-0.932547000	2.534764000

C	1.817183000	-0.756083000	4.417891000
C	2.149336000	-1.990974000	3.833861000
C	3.171422000	-2.074693000	2.918110000
H	1.373595000	-5.570028000	-0.948084000
H	3.774805000	-6.063648000	-1.233363000
H	6.065200000	-5.349131000	-1.286503000
H	7.840874000	0.760463000	0.872885000
H	6.328106000	2.108474000	2.167423000
H	4.271187000	2.345240000	3.508080000
H	2.353875000	1.308606000	4.598208000
H	0.990967000	-0.689571000	5.118369000
H	1.595628000	-2.884605000	4.102732000
H	3.413797000	-3.035444000	2.481192000
C	0.327921000	-3.187216000	-0.297770000
C	2.222371000	-1.208470000	0.183909000
C	-0.082837000	-1.915079000	-0.006462000
C	0.876155000	-0.899896000	0.207981000
H	-0.406571000	-3.955266000	-0.525237000
H	-1.133442000	-1.663702000	0.027417000
H	2.931373000	-0.400411000	0.295373000
C	0.090640000	1.576852000	0.639358000
C	0.453252000	0.440571000	0.442477000
H	0.682903000	4.153481000	-2.522376000
H	0.866200000	6.090147000	1.092220000
C	-4.935935000	4.618848000	-1.444225000
H	-4.883376000	3.749992000	-0.778376000
H	-5.419880000	4.309043000	-2.380617000
C	-5.714181000	5.743301000	-0.776867000
H	-6.782922000	5.561179000	-0.933389000
H	-5.912258000	5.071672000	1.279973000

H	-5.475136000	6.674284000	-1.303159000
H	-5.977152000	6.813714000	1.068624000
C	-5.465103000	5.907144000	0.728665000
C	-4.000729000	6.002207000	1.132466000
H	-3.894835000	6.482119000	2.114353000
H	-3.444449000	6.588462000	0.392234000

E= - 3610.0759906 Hartrees.

Compound **1**. Conformer VIII.

C	-0.133905000	-4.086097000	-1.607099000
C	1.020324000	-3.390268000	-1.933488000
C	1.041080000	-1.990384000	-1.917912000
C	-0.136772000	-1.284333000	-1.585648000
C	-1.290712000	-2.003682000	-1.250271000
C	-1.289567000	-3.391064000	-1.260392000
C	2.264600000	-1.320440000	-2.228851000
C	-0.172074000	0.138717000	-1.598280000
C	3.346559000	-0.841014000	-2.487101000
C	4.644768000	-0.426599000	-2.913849000
C	5.310115000	-1.220607000	-3.859774000
C	6.587498000	-0.901158000	-4.294638000
C	7.228075000	0.230811000	-3.792040000
C	6.582798000	1.031873000	-2.862219000
C	5.292792000	0.720170000	-2.406883000
C	4.685240000	1.584805000	-1.448443000
C	4.255546000	2.402376000	-0.664355000
C	-0.248557000	1.345999000	-1.612807000
C	-0.265694000	2.766966000	-1.539493000
C	3.699107000	3.463460000	0.110440000
C	2.613579000	3.252307000	0.995928000
C	1.961039000	4.347456000	1.556610000

C	2.369251000	5.649313000	1.262422000
C	3.517301000	5.858105000	0.492887000
C	4.155294000	4.768432000	-0.085369000
C	0.906007000	3.495114000	-1.795118000
C	0.955498000	4.860193000	-1.596228000
C	-0.157485000	5.530849000	-1.079649000
C	-1.356392000	4.846973000	-0.904338000
C	-1.427413000	3.469780000	-1.159458000
O	0.016556000	6.845389000	-0.782247000
O	1.584891000	6.639339000	1.748294000
H	-0.122449000	-5.170792000	-1.611846000
H	1.929741000	-3.922539000	-2.187745000
H	-2.187019000	-1.457065000	-0.979013000
H	-2.198471000	-3.925707000	-1.007016000
H	4.803979000	-2.102549000	-4.236379000
H	7.082324000	-1.531452000	-5.026354000
H	8.227110000	0.489534000	-4.127123000
H	7.071456000	1.914827000	-2.464689000
H	1.090141000	4.199461000	2.183769000
H	3.896190000	6.854202000	0.303761000
H	1.871984000	5.413929000	-1.758853000
H	-2.255017000	5.354652000	-0.577813000
C	-2.923617000	-0.914741000	2.338676000
C	-4.119178000	-0.130630000	2.297093000
C	-7.694184000	0.744158000	-1.708329000
C	-8.954720000	0.172965000	-2.068086000
C	-6.979450000	-1.473052000	-0.867696000
C	-6.671508000	-0.087044000	-1.187995000
C	-6.116633000	-2.335310000	-0.088842000
C	-5.147354000	-1.877214000	0.882043000

C	-4.085367000	-4.082772000	0.782248000
C	-4.053698000	-2.712575000	1.170638000
C	-5.178067000	-4.593227000	0.152304000
C	-6.214895000	-3.730216000	-0.306337000
C	-8.150119000	-2.024879000	-1.420871000
C	-9.144638000	-1.167208000	-1.992388000
C	-4.189452000	1.114041000	2.961306000
H	-5.256031000	-5.656378000	-0.055523000
C	-8.330435000	-3.437862000	-1.440094000
C	-7.348769000	-4.261380000	-0.985191000
H	-7.431268000	-5.338721000	-1.095092000
C	-2.918170000	-2.183654000	1.865265000
C	-5.261320000	-0.635253000	1.629989000
C	-5.371393000	1.810706000	3.029771000
C	-6.533579000	1.262208000	2.458417000
C	-6.478208000	0.069473000	1.777107000
H	-9.728744000	0.822965000	-2.465520000
H	-10.067265000	-1.616604000	-2.348221000
H	-9.230987000	-3.842850000	-1.892189000
H	-3.249649000	-4.718774000	1.061354000
H	-2.034224000	-2.807278000	1.955702000
H	-2.038633000	-0.496250000	2.803702000
H	-3.291855000	1.500959000	3.435284000
H	-5.416532000	2.764096000	3.546176000
H	-7.481868000	1.780600000	2.555841000
H	-7.385296000	-0.340894000	1.350677000
C	-7.432269000	2.117435000	-1.923739000
C	-5.375756000	0.459460000	-1.065740000
C	-6.189216000	2.646993000	-1.698255000
C	-5.126083000	1.799892000	-1.296802000

H	-8.235356000	2.748989000	-2.292749000
H	-5.993469000	3.700494000	-1.864894000
H	-4.546146000	-0.174563000	-0.785158000
C	-2.699610000	2.821717000	-1.100360000
C	-3.806366000	2.333902000	-1.163027000
C	6.505539000	-0.854587000	0.520427000
C	5.750292000	-1.163121000	1.695478000
C	0.737631000	-3.158856000	2.549413000
C	0.192542000	-4.469125000	2.723824000
C	2.420023000	-4.103079000	0.999980000
C	1.776516000	-2.950408000	1.609333000
C	3.677482000	-4.048503000	0.282708000
C	4.714756000	-3.055439000	0.479313000
C	5.709161000	-3.754710000	-1.651729000
C	5.633227000	-2.839473000	-0.564389000
C	4.934614000	-4.871057000	-1.665782000
C	3.904784000	-5.045080000	-0.697878000
C	1.726969000	-5.327370000	1.049514000
C	0.619085000	-5.491317000	1.942042000
C	5.881224000	-0.375960000	2.861294000
H	5.058366000	-5.627352000	-2.435485000
C	2.102532000	-6.393981000	0.185454000
C	3.096533000	-6.217739000	-0.725070000
H	3.323559000	-6.986999000	-1.457474000
C	6.508826000	-1.707163000	-0.530933000
C	4.920047000	-2.311699000	1.713262000
C	5.278399000	-0.749741000	4.037496000
C	4.554547000	-1.954084000	4.091313000
C	4.384442000	-2.714384000	2.958765000
H	-0.610343000	-4.611270000	3.441975000

H	0.148874000	-6.468797000	2.002050000
H	1.536970000	-7.320643000	0.221039000
H	6.443554000	-3.572918000	-2.430339000
H	7.139923000	-1.521677000	-1.395197000
H	7.120245000	0.040408000	0.511997000
H	6.488392000	0.523491000	2.814008000
H	5.387064000	-0.138753000	4.927700000
H	4.128212000	-2.289387000	5.031169000
H	3.834570000	-3.644650000	3.025010000
C	0.208671000	-2.055092000	3.256855000
C	2.100202000	-1.622090000	1.263753000
C	0.607293000	-0.776435000	2.970158000
C	1.528378000	-0.547068000	1.920424000
H	-0.550662000	-2.234694000	4.013059000
H	0.202693000	0.070894000	3.513716000
H	2.799440000	-1.422420000	0.462316000
C	2.184871000	1.927144000	1.293486000
C	1.879951000	0.788147000	1.563085000
H	1.792124000	2.958468000	-2.116853000
H	5.008648000	4.931162000	-0.735038000
C	-0.955979000	7.468555000	0.044357000
H	-1.167171000	6.830255000	0.912578000
H	-1.888342000	7.602628000	-0.520751000
C	-0.424430000	8.824689000	0.483517000
H	-1.279371000	9.428167000	0.808224000
H	0.159753000	8.461166000	2.545614000
H	-0.009938000	9.323450000	-0.400411000
H	0.928067000	9.849102000	1.791986000
C	0.613289000	8.815199000	1.613082000
C	1.856511000	7.976461000	1.348214000

H	2.714711000	8.353561000	1.920223000
H	2.105770000	8.006955000	0.281678000

E = - 3610.0654052 Hartrees.

Compound **1**. Conformer IX.

C	3.593263000	-4.520048000	1.504206000
C	2.379340000	-4.261912000	0.884162000
C	1.787620000	-2.992841000	0.949607000
C	2.457288000	-1.965312000	1.651522000
C	3.676479000	-2.245795000	2.282023000
C	4.238747000	-3.511334000	2.214194000
C	0.530882000	-2.803172000	0.292308000
C	1.950664000	-0.633868000	1.689079000
C	-0.530675000	-2.803149000	-0.292528000
C	-1.787482000	-2.992786000	-0.949714000
C	-2.379264000	-4.261814000	-0.884037000
C	-3.593258000	-4.519985000	-1.503934000
C	-4.238752000	-3.511352000	-2.214024000
C	-3.676433000	-2.245843000	-2.282066000
C	-2.457184000	-1.965327000	-1.651706000
C	-1.950484000	-0.633919000	-1.689440000
C	-1.585758000	0.519203000	-1.709055000
C	1.586048000	0.519291000	1.708624000
C	1.072774000	1.847384000	1.749767000
C	-1.072474000	1.847290000	-1.750109000
C	0.326510000	2.060918000	-1.783216000
C	0.827251000	3.357906000	-1.845417000
C	-0.035741000	4.451870000	-1.903359000
C	-1.417407000	4.249284000	-1.854691000
C	-1.918711000	2.955372000	-1.774656000
C	1.919047000	2.955441000	1.774605000

C	1.417757000	4.249343000	1.854830000
C	0.036091000	4.451952000	1.903388000
C	-0.826927000	3.358030000	1.845158000
C	-0.326213000	2.061032000	1.782782000
O	-0.573934000	5.659586000	2.045680000
O	0.574342000	5.659502000	-2.045405000
H	4.041629000	-5.504555000	1.423353000
H	1.871483000	-5.039983000	0.324836000
H	4.191139000	-1.444290000	2.802027000
H	5.197480000	-3.699048000	2.685815000
H	-1.871405000	-5.039827000	-0.324630000
H	-4.041673000	-5.504451000	-1.422851000
H	-5.197537000	-3.699091000	-2.685528000
H	-4.191081000	-1.444389000	-2.802160000
H	1.897447000	3.525934000	-1.874492000
H	-2.110712000	5.080831000	-1.887124000
H	2.111063000	5.080878000	1.887592000
H	-1.897121000	3.526080000	1.874186000
C	-5.291499000	2.971376000	-0.081521000
C	-5.474660000	2.181565000	1.097072000
C	-4.561524000	-3.327931000	1.907117000
C	-5.402715000	-4.480844000	1.867938000
C	-6.044348000	-2.229179000	0.240829000
C	-4.850545000	-2.215388000	1.075700000
C	-6.482624000	-1.111819000	-0.578707000
C	-6.204186000	0.290860000	-0.332974000
C	-6.737454000	0.755498000	-2.684018000
C	-6.203310000	1.171312000	-1.431995000
C	-7.320736000	-0.466187000	-2.801909000
C	-7.211767000	-1.422442000	-1.752594000

C	-6.726072000	-3.455277000	0.110844000
C	-6.417882000	-4.553919000	0.974419000
C	-5.194745000	2.721233000	2.372056000
H	-7.835147000	-0.753355000	-3.714257000
C	-7.665638000	-3.650843000	-0.941194000
C	-7.822823000	-2.700677000	-1.898609000
H	-8.439429000	-2.884586000	-2.773608000
C	-5.704253000	2.506192000	-1.285088000
C	-6.000151000	0.869050000	0.987033000
C	-5.481163000	2.015605000	3.515372000
C	-6.094098000	0.754664000	3.413928000
C	-6.347668000	0.198720000	2.182029000
H	-5.177798000	-5.315796000	2.525199000
H	-7.014840000	-5.457277000	0.886787000
H	-8.175488000	-4.606917000	-1.015452000
H	-6.742911000	1.459289000	-3.510950000
H	-5.651987000	3.133663000	-2.170646000
H	-4.875829000	3.970206000	0.018032000
H	-4.767195000	3.718464000	2.432551000
H	-5.262134000	2.438556000	4.490184000
H	-6.370478000	0.213478000	4.312810000
H	-6.827401000	-0.770307000	2.126733000
C	-3.405365000	-3.317565000	2.722264000
C	-3.902317000	-1.174433000	1.038022000
C	-2.538657000	-2.256622000	2.717395000
C	-2.788358000	-1.164944000	1.854886000
H	-3.206336000	-4.181297000	3.350133000
H	-1.651554000	-2.251292000	3.340433000
H	-4.009016000	-0.360317000	0.338585000
C	-1.210252000	0.943398000	1.788253000

C	-1.910923000	-0.040812000	1.816757000
C	5.290772000	2.971217000	0.081252000
C	5.474139000	2.181367000	-1.097285000
C	4.561481000	-3.328300000	-1.906722000
C	5.402602000	-4.481251000	-1.867225000
C	6.044276000	-2.229224000	-0.240589000
C	4.850514000	-2.215575000	-1.075543000
C	6.482544000	-1.111729000	0.578781000
C	6.203980000	0.290906000	0.332924000
C	6.737203000	0.755829000	2.683914000
C	6.202960000	1.171447000	1.431870000
C	7.320667000	-0.465761000	2.801889000
C	7.211761000	-1.422145000	1.752684000
C	6.725991000	-3.455308000	-0.110374000
C	6.417764000	-4.554143000	-0.973691000
C	5.194164000	2.720878000	-2.372319000
H	7.835145000	-0.752776000	3.714248000
C	7.665619000	-3.650674000	0.941642000
C	7.822875000	-2.700329000	1.898867000
H	8.439558000	-2.884072000	2.773846000
C	5.703619000	2.506210000	1.284854000
C	5.999875000	0.868959000	-0.987133000
C	5.480728000	2.015196000	-3.515567000
C	6.093842000	0.754353000	-3.414010000
C	6.347483000	0.198555000	-2.182058000
H	5.177655000	-5.316351000	-2.524286000
H	7.014699000	-5.457496000	-0.885852000
H	8.175501000	-4.606720000	1.016032000
H	6.742576000	1.459690000	3.510787000
H	5.651224000	3.133742000	2.170364000

H	4.874849000	3.969936000	-0.018364000
H	4.766449000	3.718032000	-2.432919000
H	5.261653000	2.438034000	-4.490418000
H	6.370300000	0.213131000	-4.312847000
H	6.827302000	-0.770424000	-2.126645000
C	3.405410000	-3.318049000	-2.721991000
C	3.902335000	-1.174571000	-1.038205000
C	2.538780000	-2.257035000	-2.717484000
C	2.788474000	-1.165188000	-1.855198000
H	3.206380000	-4.181917000	-3.349673000
H	1.651752000	-2.251792000	-3.340630000
H	4.008974000	-0.360304000	-0.338944000
C	1.210503000	0.943255000	-1.788853000
C	1.911099000	-0.041006000	-1.817312000
H	2.990454000	2.794340000	1.729127000
H	-2.990116000	2.794302000	-1.729085000
C	0.184591000	6.852761000	1.918883000
H	-0.487197000	7.636810000	2.279042000
H	1.055071000	6.831265000	2.586825000
C	0.598799000	7.122999000	0.476574000
H	1.131966000	8.080238000	0.437348000
H	-1.303059000	6.353152000	-0.149372000
H	1.303517000	6.353178000	0.149593000
H	-1.131544000	8.080211000	-0.437169000
C	-0.598361000	7.122979000	-0.476376000
C	-0.184170000	6.852691000	-1.918679000
H	-1.054664000	6.831169000	-2.586600000
H	0.487614000	7.636720000	-2.278892000

E = -3610.0766722 Hartrees.

Derivative **1** of Compound **1** in effect of π-extension section

C	5.069402000	-3.652655000	-1.362557000
C	4.828673000	-2.391682000	-0.837824000
C	3.537831000	-1.842296000	-0.840941000
C	2.475435000	-2.586371000	-1.397744000
C	2.736768000	-3.861742000	-1.919008000
C	4.018251000	-4.391937000	-1.902227000
C	3.359465000	-0.546511000	-0.262721000
C	1.139415000	-2.084359000	-1.441197000
C	3.359449000	0.546110000	0.262767000
C	3.537882000	1.841942000	0.840861000
C	4.828785000	2.391187000	0.837820000
C	5.069540000	3.652317000	1.362165000
C	4.018350000	4.391914000	1.901322000
C	2.736811000	3.861853000	1.918049000
C	2.475453000	2.586307000	1.397223000
C	1.139394000	2.084414000	1.440675000
C	-0.018856000	1.736669000	1.509482000
C	-0.018798000	-1.736493000	-1.510041000
C	-1.396763000	-1.368409000	-1.546989000
C	-1.396747000	1.368350000	1.546794000
C	-1.794253000	0.018893000	1.715254000
C	-3.146136000	-0.310352000	1.698828000
C	-4.118079000	0.673062000	1.500191000
C	-3.736100000	2.010325000	1.363118000
C	-2.386483000	2.340160000	1.384384000
C	-2.386291000	-2.340408000	-1.384390000
C	-3.735964000	-2.010830000	-1.362900000
C	-4.118223000	-0.673637000	-1.499934000
C	-3.146501000	0.309942000	-1.698814000
C	-1.794548000	-0.019040000	-1.715425000

O	-5.393310000	-0.231795000	-1.441402000
O	-5.393089000	0.230987000	1.441830000
H	6.074647000	-4.059928000	-1.347326000
H	5.639152000	-1.813386000	-0.407502000
H	1.913795000	-4.431123000	-2.336978000
H	4.197388000	-5.380949000	-2.310221000
H	5.639290000	1.812651000	0.407869000
H	6.074834000	4.059474000	1.347020000
H	4.197499000	5.381079000	2.308938000
H	1.913807000	4.431484000	2.335613000
H	-3.457779000	-1.343891000	1.792791000
H	-4.470677000	2.794379000	1.230345000
H	-4.470364000	-2.795028000	-1.230008000
H	-3.458346000	1.343419000	-1.792782000
C	3.182516000	4.680363000	-1.775782000
C	3.421092000	3.405868000	-2.282965000
C	1.904284000	5.030094000	-1.344323000
C	2.390970000	2.475942000	-2.350992000
C	0.868732000	4.107595000	-1.409585000
C	1.106835000	2.818369000	-1.906892000
H	1.717414000	6.021485000	-0.944854000
H	-0.124056000	4.366232000	-1.056867000
H	2.576433000	1.473417000	-2.720527000
C	-0.806906000	1.002342000	-1.851282000
C	0.061977000	1.843028000	-1.908041000
C	3.184274000	-4.679032000	1.776488000
C	3.422232000	-3.404522000	2.283936000
C	1.906355000	-5.029095000	1.344382000
C	2.391814000	-2.474902000	2.351551000
C	0.870493000	-4.106914000	1.409280000

C	1.107981000	-2.817673000	1.906842000
H	1.719972000	-6.020496000	0.944713000
H	-0.122055000	-4.365793000	1.056065000
H	2.576803000	-1.472364000	2.721290000
C	-0.806342000	-1.002256000	1.850880000
C	0.062815000	-1.842659000	1.907674000
H	-2.088757000	-3.376140000	-1.260269000
H	-2.089162000	3.375943000	1.260176000
C	-6.419743000	-1.140305000	-1.040748000
H	-6.085825000	-1.718117000	-0.171782000
H	-6.627786000	-1.832502000	-1.865635000
C	-7.655048000	-0.322559000	-0.696023000
H	-8.527783000	-0.978878000	-0.772569000
H	-7.774385000	-0.448249000	1.467733000
H	-7.774679000	0.447111000	-1.466846000
H	-8.527786000	0.977635000	0.773562000
C	-7.654979000	0.321429000	0.696883000
C	-6.419732000	1.139349000	1.041395000
H	-6.627718000	1.831497000	1.866339000
H	-6.086067000	1.717228000	0.172376000
H	3.994854000	-5.397350000	1.711071000
H	4.417940000	-3.127442000	2.614185000
H	4.417038000	3.129039000	-2.612702000
H	3.992853000	5.398925000	-1.710041000

E = -2074.1543062 Hartrees.

Derivative **2** of Compound **1** in effect of π-extension section

C	-4.353856000	-3.364534000	1.975924000
C	-4.119193000	-2.207970000	1.249080000
C	-2.830900000	-1.658785000	1.161542000
C	-1.766017000	-2.291854000	1.837259000

C	-2.022415000	-3.462334000	2.566908000
C	-3.300615000	-3.995263000	2.636023000
C	-2.657273000	-0.486693000	0.361442000
C	-0.429723000	-1.790295000	1.790816000
C	-2.657145000	0.486719000	-0.361503000
C	-2.830797000	1.658828000	-1.161574000
C	-4.119087000	2.208029000	-1.249045000
C	-4.353767000	3.364620000	-1.975844000
C	-3.300548000	3.995356000	-2.635967000
C	-2.022348000	3.462421000	-2.566904000
C	-1.765933000	2.291916000	-1.837301000
C	-0.429647000	1.790334000	-1.790950000
C	0.731746000	1.445206000	-1.796556000
C	0.731685000	-1.445216000	1.796383000
C	2.110543000	-1.079229000	1.765487000
C	2.110606000	1.079235000	-1.765601000
C	2.509856000	-0.278910000	-1.697106000
C	3.861542000	-0.599930000	-1.621215000
C	4.832500000	0.403829000	-1.594195000
C	4.449469000	1.744373000	-1.691232000
C	3.099824000	2.065047000	-1.771926000
C	3.099780000	-2.065026000	1.771792000
C	4.449424000	-1.744329000	1.691171000
C	4.832432000	-0.403768000	1.594252000
C	3.861457000	0.599967000	1.621282000
C	2.509769000	0.278922000	1.697093000
O	6.107633000	0.020463000	1.459166000
O	6.107707000	-0.020359000	-1.459023000
H	-5.354403000	-3.780533000	2.017552000
H	-4.929326000	-1.720954000	0.718211000

H	-1.197535000	-3.948726000	3.076038000
H	-3.475306000	-4.904799000	3.200835000
H	-4.929199000	1.721016000	-0.718142000
H	-5.354307000	3.780643000	-2.017404000
H	-3.475245000	4.904916000	-3.200739000
H	-1.197486000	3.948820000	-3.076058000
H	4.173733000	-1.634008000	-1.535478000
H	5.183661000	2.539975000	-1.694966000
H	5.183628000	-2.539920000	1.694884000
H	4.173631000	1.634061000	1.535652000
C	-2.599168000	4.793631000	0.916023000
C	-3.708180000	5.636944000	0.652422000
C	-4.125745000	3.240163000	2.038807000
C	-2.811114000	3.577800000	1.622535000
C	-5.179432000	4.074360000	1.766298000
C	-4.968064000	5.286481000	1.067228000
H	-3.544256000	6.563218000	0.109170000
H	-5.810740000	5.937077000	0.856458000
C	-1.284731000	5.116204000	0.482383000
C	-1.712768000	2.717998000	1.861327000
C	-0.238114000	4.269979000	0.720092000
C	-0.449918000	3.041776000	1.410625000
H	-1.124615000	6.044539000	-0.058046000
H	0.759555000	4.508794000	0.367779000
H	-1.878120000	1.774010000	2.370287000
C	1.518395000	1.304002000	1.657987000
C	0.627270000	2.119086000	1.576928000
C	-2.598975000	-4.793758000	-0.916083000
C	-3.707978000	-5.637091000	-0.652495000
C	-4.125591000	-3.240251000	-2.038759000

C	-2.810948000	-3.577895000	-1.622531000
C	-5.179266000	-4.074470000	-1.766270000
C	-4.967872000	-5.286622000	-1.067258000
H	-3.544029000	-6.563391000	-0.109294000
H	-5.810539000	-5.937233000	-0.856499000
C	-1.284524000	-5.116348000	-0.482499000
C	-1.712617000	-2.718074000	-1.861310000
C	-0.237921000	-4.270099000	-0.720188000
C	-0.449758000	-3.041859000	-1.410652000
H	-1.124383000	-6.044720000	0.057859000
H	0.759760000	-4.508918000	-0.367912000
H	-1.877989000	-1.774065000	-2.370227000
C	1.518491000	-1.304005000	-1.657999000
C	0.627407000	-2.119130000	-1.576904000
H	2.802272000	-3.106589000	1.829273000
H	2.802309000	3.106603000	-1.829496000
C	7.133533000	-0.944048000	1.221265000
H	6.799222000	-1.662882000	0.464972000
H	7.341149000	-1.483879000	2.153114000
C	8.369091000	-0.197932000	0.741403000
H	9.241702000	-0.831598000	0.929325000
H	8.488852000	-0.692156000	-1.368820000
H	8.488764000	0.692336000	1.369090000
H	9.241728000	0.831801000	-0.929031000
C	8.369125000	0.198115000	-0.741147000
C	7.133566000	0.944185000	-1.221085000
H	7.341220000	1.483999000	-2.152934000
H	6.799189000	1.663035000	-0.464834000
H	-4.282605000	2.304339000	2.567696000
H	-6.182044000	3.806721000	2.084043000

H	-4.282470000	-2.304402000	-2.567601000
H	-6.181887000	-3.806823000	-2.083978000

E= -2381.3540447

Derivative **3** of Compound **1** in effect of π-extension section

C	-3.657779000	-3.072339000	2.349437000
C	-3.407875000	-1.992784000	1.516568000
C	-2.101615000	-1.513714000	1.335415000
C	-1.036384000	-2.123798000	2.030760000
C	-1.308155000	-3.218977000	2.863347000
C	-2.603117000	-3.692044000	3.018351000
C	-1.909451000	-0.442452000	0.413539000
C	0.306866000	-1.653385000	1.911734000
C	-1.909538000	0.442457000	-0.413828000
C	-2.101759000	1.513768000	-1.335635000
C	-3.407995000	1.992911000	-1.516700000
C	-3.657888000	3.072494000	-2.349543000
C	-2.603244000	3.692122000	-3.018550000
C	-1.308293000	3.218987000	-2.863622000
C	-1.036536000	2.123814000	-2.031023000
C	0.306717000	1.653430000	-1.911948000
C	1.463902000	1.296392000	-1.874985000
C	1.464059000	-1.296367000	1.874791000
C	2.842775000	-0.932582000	1.817685000
C	2.842618000	0.932609000	-1.817914000
C	3.245786000	-0.419128000	-1.675950000
C	4.598836000	-0.730451000	-1.582375000
C	5.566729000	0.276373000	-1.614304000
C	5.179591000	1.607719000	-1.788877000
C	3.828981000	1.919071000	-1.883339000
C	3.829136000	-1.919058000	1.883207000

C	5.179751000	-1.607699000	1.788954000
C	5.566923000	-0.276341000	1.614461000
C	4.599027000	0.730472000	1.582387000
C	3.245955000	0.419143000	1.675785000
O	6.843700000	0.134623000	1.455389000
O	6.843440000	-0.134598000	-1.454796000
H	-4.671548000	-3.439968000	2.464499000
H	-4.216026000	-1.515605000	0.972973000
H	-0.485369000	-3.692576000	3.387746000
H	-2.790926000	-4.543710000	3.663439000
H	-4.216138000	1.515802000	-0.973036000
H	-4.671644000	3.440183000	-2.464534000
H	-2.791056000	4.543769000	-3.663663000
H	-0.485514000	3.692538000	-3.388074000
H	4.914832000	-1.757098000	-1.439957000
H	5.911122000	2.403895000	-1.841698000
H	5.911295000	-2.403855000	1.841915000
H	4.915035000	1.757125000	1.440033000
C	-2.007991000	4.670034000	0.648598000
C	-3.164874000	5.445845000	0.303316000
C	-3.504224000	2.969710000	1.656570000
C	-2.163910000	3.446331000	1.343742000
C	-3.746806000	1.732458000	2.291588000
C	-5.030964000	1.280546000	2.508882000
C	-4.618457000	3.750810000	1.262820000
C	-4.410972000	5.007774000	0.598435000
C	-5.925298000	3.274199000	1.503887000
C	-6.133537000	2.055439000	2.110351000
H	-7.142453000	1.694640000	2.281802000
H	-3.018609000	6.388147000	-0.216406000

H	-5.282188000	5.593912000	0.320513000
H	-6.768932000	3.883980000	1.192925000
C	-0.714828000	5.102886000	0.279825000
C	-1.011242000	2.700032000	1.645891000
C	0.395774000	4.345398000	0.558554000
C	0.248834000	3.117980000	1.243846000
H	-0.611432000	6.043082000	-0.253718000
H	1.382190000	4.667435000	0.243138000
H	-1.087644000	1.749504000	2.157038000
C	2.256298000	1.443206000	1.585179000
C	1.365084000	2.252956000	1.459083000
C	-2.008005000	-4.670109000	-0.648545000
C	-3.164879000	-5.445857000	-0.303089000
C	-3.504294000	-2.969772000	-1.656417000
C	-2.163964000	-3.446415000	-1.343705000
C	-3.746897000	-1.732598000	-2.291576000
C	-5.031062000	-1.280655000	-2.508776000
C	-4.618512000	-3.750776000	-1.262439000
C	-4.410999000	-5.007719000	-0.598020000
C	-5.925357000	-3.274124000	-1.503385000
C	-6.133619000	-2.055432000	-2.109981000
H	-7.142541000	-1.694609000	-2.281338000
H	-3.018593000	-6.388134000	0.216673000
H	-5.282206000	-5.593780000	-0.319907000
H	-6.768981000	-3.883824000	-1.192237000
C	-0.714822000	-5.102978000	-0.279879000
C	-1.011316000	-2.700146000	-1.645954000
C	0.395774000	-4.345506000	-0.558710000
C	0.248796000	-3.118106000	-1.244002000
H	-0.611395000	-6.043170000	0.253664000

H	1.382212000	-4.667563000	-0.243380000
H	-1.087741000	-1.749599000	-2.157058000
C	2.256151000	-1.443222000	-1.585371000
C	1.365005000	-2.253049000	-1.459299000
H	3.528046000	-2.954631000	1.999349000
H	3.527922000	2.954652000	-1.999490000
C	7.868305000	-0.846430000	1.288763000
H	7.533551000	-1.618313000	0.587095000
H	8.074425000	-1.316716000	2.257861000
C	9.104886000	-0.140080000	0.754323000
H	9.977021000	-0.757852000	0.990897000
H	9.224807000	-0.795818000	-1.311550000
H	9.224791000	0.795968000	1.311374000
H	9.976811000	0.758125000	-0.991095000
C	9.104802000	0.140188000	-0.754460000
C	7.868101000	0.846422000	-1.288730000
H	8.073968000	1.316503000	-2.257989000
H	7.533630000	1.618504000	-0.587132000
H	-5.186466000	-0.318671000	-2.986878000
H	-2.919558000	-1.106901000	-2.603443000
H	-5.186359000	0.318486000	2.986836000
H	-2.919464000	1.106649000	2.603217000

E= -2688.5620123

<u>Derivative **4** of Compound **1** in effect of π-extension section</u>

C	-3.218312000	3.009961000	-2.534132000
C	-3.014273000	1.998299000	-1.608789000
C	-1.745053000	1.434749000	-1.428296000
C	-0.663269000	1.900459000	-2.206307000
C	-0.889709000	2.919557000	-3.142917000
C	-2.153467000	3.470374000	-3.305595000

C	-1.598653000	0.417454000	-0.438568000
C	0.662083000	1.386273000	-2.055953000
C	-1.598614000	-0.417783000	0.439019000
C	-1.744877000	-1.435117000	1.428725000
C	-3.014046000	-1.998765000	1.609286000
C	-3.217949000	-3.010457000	2.534625000
C	-2.153016000	-3.470805000	3.306008000
C	-0.889309000	-2.919894000	3.143257000
C	-0.663007000	-1.900759000	2.206653000
C	0.662299000	-1.386495000	2.056177000
C	1.820337000	-1.035440000	1.987740000
C	1.820147000	1.035274000	-1.987660000
C	3.203980000	0.695204000	-1.893017000
C	3.204154000	-0.695311000	1.893041000
C	3.627487000	0.625704000	1.602124000
C	4.984696000	0.907687000	1.483420000
C	5.938859000	-0.101199000	1.630004000
C	5.533308000	-1.400935000	1.944038000
C	4.177880000	-1.681745000	2.067436000
C	4.177657000	1.681678000	-2.067477000
C	5.533103000	1.400921000	-1.944174000
C	5.938725000	0.101203000	-1.630155000
C	4.984613000	-0.907717000	-1.483482000
C	3.627383000	-0.625789000	-1.602102000
O	7.220543000	-0.277111000	-1.436215000
O	7.220642000	0.277160000	1.435931000
H	-4.207074000	3.440285000	-2.650216000
H	-3.836797000	1.634660000	-1.003039000
H	-0.055714000	3.276366000	-3.737532000
H	-2.305614000	4.261991000	-4.031643000

H	-3.836631000	-1.635173000	1.003592000
H	-4.206672000	-3.440854000	2.650774000
H	-2.305055000	-4.262456000	4.032042000
H	-0.055241000	-3.276671000	3.737791000
H	5.314437000	1.908648000	1.231605000
H	6.254941000	-2.196011000	2.083202000
H	6.254698000	2.196019000	-2.083418000
H	5.314410000	-1.908662000	-1.231680000
C	-1.547245000	-4.941049000	-0.432922000
C	-2.687672000	-5.667763000	0.022672000
C	-3.065214000	-3.282898000	-1.486367000
C	-1.723001000	-3.723879000	-1.147677000
C	-3.365293000	-2.186488000	-2.393301000
C	-2.460037000	-1.693997000	-3.368043000
C	-4.049233000	-0.029003000	-4.102288000
C	-2.788507000	-0.643266000	-4.194018000
C	-4.978129000	-0.528799000	-3.222396000
C	-4.673041000	-1.626484000	-2.384568000
C	-4.141973000	-3.939065000	-0.865062000
C	-3.931506000	-5.151143000	-0.137009000
H	-5.976250000	-0.102585000	-3.174324000
C	-5.458685000	-3.382748000	-0.921970000
C	-5.700145000	-2.229139000	-1.592646000
H	-6.690079000	-1.782635000	-1.593844000
H	-2.536220000	-6.601620000	0.555468000
H	-4.797156000	-5.656978000	0.280456000
H	-6.255096000	-3.887298000	-0.382879000
H	-4.292764000	0.814484000	-4.739232000
C	-0.243600000	-5.382683000	-0.100741000
C	-0.572302000	-2.936473000	-1.369830000

C	0.862739000	-4.625885000	-0.387743000
C	0.692903000	-3.362829000	-1.002011000
H	-0.134822000	-6.328353000	0.421973000
H	1.856137000	-4.962411000	-0.112029000
H	-0.664149000	-1.932867000	-1.756477000
C	2.657610000	-1.651793000	-1.402929000
C	1.791743000	-2.476506000	-1.215915000
C	-1.546943000	4.941286000	0.433118000
C	-2.687293000	5.668120000	-0.022483000
C	-3.065094000	3.283138000	1.486300000
C	-1.722831000	3.724060000	1.147742000
C	-3.365299000	2.186634000	2.393077000
C	-2.460147000	1.693984000	3.367836000
C	-4.049472000	0.028971000	4.101745000
C	-2.788727000	0.643166000	4.193661000
C	-4.978272000	0.528915000	3.221839000
C	-4.673071000	1.626695000	2.384172000
C	-4.141770000	3.939439000	0.864995000
C	-3.931174000	5.151573000	0.137073000
H	-5.976408000	0.102752000	3.173637000
C	-5.458518000	3.383193000	0.921749000
C	-5.700089000	2.229510000	1.592259000
H	-6.690049000	1.783062000	1.593333000
H	-2.535740000	6.602008000	-0.555194000
H	-4.796761000	5.657501000	-0.280407000
H	-6.254865000	3.887858000	0.382672000
H	-4.293087000	-0.814592000	4.738557000
C	-0.243244000	5.382856000	0.101058000
C	-0.572208000	2.936542000	1.369878000
C	0.863022000	4.625942000	0.388047000

C	0.693049000	3.362833000	1.002178000
H	-0.134363000	6.328576000	-0.421545000
H	1.856464000	4.962423000	0.112436000
H	-0.664151000	1.932902000	1.756406000
C	2.657669000	1.651677000	1.403022000
C	1.791819000	2.476417000	1.216056000
H	3.862507000	2.694460000	-2.294375000
H	3.862785000	-2.694543000	2.294339000
C	8.235295000	0.724779000	-1.359843000
H	7.892062000	1.554683000	-0.732332000
H	8.439680000	1.105963000	-2.367703000
C	9.476276000	0.077255000	-0.763850000
H	10.344306000	0.679775000	-1.049771000
H	9.604693000	0.901039000	1.240373000
H	9.604640000	-0.900908000	-1.240840000
H	10.344407000	-0.679616000	1.049254000
C	9.476334000	-0.077130000	0.763394000
C	8.235417000	-0.724697000	1.359472000
H	8.439889000	-1.105898000	2.367308000
H	7.892157000	-1.554597000	0.731967000
H	-1.505114000	-2.181149000	-3.511313000
H	-2.070060000	-0.296855000	-4.929666000
H	-1.505221000	2.181087000	3.511248000
H	-2.070330000	0.296657000	4.929328000

E = - 2995.7433981 Hartrees.

Derivative **5** of Compound **1** in effect of π-extension section

C	-3.060803000	2.854683000	-2.765575000
C	-2.881833000	1.921612000	-1.755514000
C	-1.632389000	1.330799000	-1.532121000
C	-0.542754000	1.697334000	-2.350333000

C	-0.738800000	2.645576000	-3.364149000
C	-1.985259000	3.219654000	-3.572363000
C	-1.506558000	0.384504000	-0.468577000
C	0.764168000	1.153549000	-2.166852000
C	-1.506540000	-0.384662000	0.468634000
C	-1.632277000	-1.330964000	1.532187000
C	-2.881677000	-1.921811000	1.755653000
C	-3.060561000	-2.854943000	2.765693000
C	-1.984957000	-3.219926000	3.572376000
C	-0.738510000	-2.645826000	3.364062000
C	-0.542562000	-1.697541000	2.350277000
C	0.764342000	-1.153747000	2.166720000
C	1.912696000	-0.779196000	2.074341000
C	1.912528000	0.779013000	-2.074469000
C	3.293624000	0.442399000	-1.956716000
C	3.293790000	-0.442561000	1.956549000
C	3.713338000	0.832714000	1.507432000
C	5.071197000	1.100222000	1.357669000
C	6.026544000	0.118713000	1.629616000
C	5.622795000	-1.131671000	2.105675000
C	4.267943000	-1.397420000	2.258563000
C	4.267745000	1.397280000	-2.258748000
C	5.622607000	1.131613000	-2.105766000
C	6.026375000	-0.118723000	-1.629608000
C	5.071064000	-1.100280000	-1.357704000
C	3.713204000	-0.832844000	-1.507536000
O	7.308132000	-0.469546000	-1.388206000
O	7.308331000	0.469645000	1.388502000
H	-4.036374000	3.306415000	-2.909460000
H	-3.712107000	1.640551000	-1.118410000

H	0.104654000	2.925915000	-3.985597000
H	-2.115433000	3.954557000	-4.359596000
H	-3.712012000	-1.640743000	1.118635000
H	-4.036123000	-3.306677000	2.909631000
H	-2.115041000	-3.954857000	4.359598000
H	0.104995000	-2.926201000	3.985424000
H	5.400048000	2.062399000	0.983321000
H	6.345150000	-1.901433000	2.346120000
H	6.344924000	1.901427000	-2.346153000
H	5.399950000	-2.062412000	-0.983273000
C	-1.330261000	-1.223916000	-4.898126000
C	-0.933829000	-2.492417000	-4.433733000
C	-1.026051000	-5.467749000	0.261101000
C	-2.019717000	-6.336753000	0.807745000
C	-2.831857000	-4.056815000	-0.710176000
C	-1.415791000	-4.304163000	-0.453762000
C	-3.353417000	-3.010677000	-1.566049000
C	-2.661014000	-2.438177000	-2.715465000
C	-4.383321000	-0.678801000	-2.840419000
C	-3.141778000	-1.231693000	-3.285722000
C	-5.139926000	-1.345696000	-1.937563000
C	-4.659546000	-2.539761000	-1.308309000
C	-3.754711000	-4.824763000	0.032558000
C	-3.327624000	-5.990221000	0.745943000
H	-6.123934000	-0.980024000	-1.659163000
C	-5.113003000	-4.417185000	0.138181000
C	-5.525024000	-3.254871000	-0.434545000
H	-6.533927000	-2.884231000	-0.280751000
C	-2.431015000	-0.618166000	-4.343239000
C	-1.585467000	-3.082540000	-3.375615000

H	-1.702590000	-7.231307000	1.334959000
H	-4.083535000	-6.592605000	1.241001000
H	-5.791987000	-5.013292000	0.740325000
H	-4.731617000	0.247035000	-3.286864000
H	-2.781685000	0.341014000	-4.713593000
H	-0.790723000	-0.746643000	-5.709540000
C	0.345138000	-5.720736000	0.506732000
C	-0.405100000	-3.368020000	-0.763548000
C	1.313193000	-4.832758000	0.119145000
C	0.931348000	-3.620833000	-0.506075000
H	0.618244000	-6.630053000	1.033721000
H	2.361927000	-5.029831000	0.312785000
H	-0.666549000	-2.410887000	-1.191527000
C	2.748087000	-1.829213000	-1.176827000
C	1.916861000	-2.652617000	-0.866958000
C	-1.330335000	1.223659000	4.897990000
C	-0.933845000	2.492198000	4.433748000
C	-1.025774000	5.467892000	-0.260884000
C	-2.019366000	6.337062000	-0.807392000
C	-2.831678000	4.057022000	0.710254000
C	-1.415610000	4.304248000	0.453827000
C	-3.353313000	3.010824000	1.566028000
C	-2.660961000	2.438180000	2.715399000
C	-4.383357000	0.678884000	2.840145000
C	-3.141794000	1.231657000	3.285526000
C	-5.139913000	1.345920000	1.937350000
C	-4.659471000	2.540036000	1.308241000
C	-3.754483000	4.825147000	-0.032345000
C	-3.327311000	5.990651000	-0.745604000
H	-6.123937000	0.980330000	1.658897000

C	-5.112821000	4.417698000	-0.137974000
C	-5.524920000	3.255340000	0.434597000
H	-6.533860000	2.884805000	0.280796000
C	-2.431079000	0.617995000	4.342995000
C	-1.585423000	3.082449000	3.375663000
H	-1.702157000	7.231651000	-1.334498000
H	-4.083176000	6.593167000	-1.240569000
H	-5.791764000	5.013957000	-0.740012000
H	-4.731709000	-0.246986000	3.286475000
H	-2.781775000	-0.341226000	4.713223000
H	-0.790848000	0.746282000	5.709379000
C	0.345447000	5.720817000	-0.506455000
C	-0.405001000	3.367968000	0.763477000
C	1.313424000	4.832721000	-0.118961000
C	0.931460000	3.620724000	0.506075000
H	0.618620000	6.630192000	-1.033311000
H	2.362182000	5.029738000	-0.312525000
H	-0.666528000	2.410804000	1.191340000
C	2.748155000	1.829036000	1.176765000
C	1.916904000	2.652427000	0.866931000
H	3.952405000	2.374460000	-2.608215000
H	3.952627000	-2.374624000	2.607988000
C	8.322656000	0.534267000	-1.443680000
H	7.979242000	1.438580000	-0.929461000
H	8.526489000	0.780399000	-2.492785000
C	9.564093000	-0.027445000	-0.767150000
H	10.432117000	0.530291000	-1.132830000
H	9.691839000	1.061564000	1.106462000
H	9.691741000	-1.061433000	-1.106786000
H	10.432277000	-0.530127000	1.132403000

C	9.564183000	0.027571000	0.766836000
C	8.322868000	-0.534199000	1.443557000
H	8.526955000	-0.780454000	2.492583000
H	7.979315000	-1.438445000	0.929326000
H	-1.270010000	-4.064169000	-3.045285000
H	-0.111198000	-3.012356000	-4.913822000
H	-1.269917000	4.064102000	3.045449000
H	-0.111228000	3.012068000	4.913933000

E = -3302.9318829 Hartrees.

Derivative **7** of Compound **1** in effect of π-extension section

C	1.748847000	-3.058506000	3.548350000
C	1.201204000	-2.867747000	2.288456000
C	0.739223000	-1.608164000	1.887985000
C	0.831406000	-0.522380000	2.784869000
C	1.396810000	-0.730000000	4.050161000
C	1.852361000	-1.985203000	4.430100000
C	0.203470000	-1.471309000	0.570973000
C	0.377284000	0.786899000	2.443345000
C	-0.203119000	-1.471479000	-0.570836000
C	-0.738402000	-1.608675000	-1.888002000
C	-1.199442000	-2.868548000	-2.288629000
C	-1.746589000	-3.059642000	-3.548691000
C	-1.850553000	-1.986387000	-4.430447000
C	-1.395952000	-0.730887000	-4.050342000
C	-0.831049000	-0.522927000	-2.784879000
C	-0.377875000	0.786639000	-2.443204000
C	-0.043631000	1.932888000	-2.237469000
C	0.042230000	1.932905000	2.237550000
C	-0.245918000	3.311074000	2.008470000
C	0.243824000	3.311213000	-2.008530000

C	1.279786000	3.722616000	-1.135172000
C	1.484406000	5.079557000	-0.899056000
C	0.667338000	6.040890000	-1.496923000
C	-0.336462000	5.644898000	-2.385048000
C	-0.537218000	4.291676000	-2.625805000
C	0.534605000	4.291958000	2.625743000
C	0.333161000	5.645076000	2.384978000
C	-0.670846000	6.040548000	1.496859000
C	-1.487421000	5.078793000	0.899008000
C	-1.282107000	3.721954000	1.135121000
O	-0.920187000	7.320299000	1.142976000
O	0.916005000	7.320774000	-1.143034000
H	2.113142000	-4.040212000	3.830870000
H	1.135619000	-3.694310000	1.589501000
H	1.472305000	0.110148000	4.731875000
H	2.291027000	-2.124567000	5.412370000
H	-1.133522000	-3.695080000	-1.589673000
H	-2.110113000	-4.041591000	-3.831352000
H	-2.288848000	-2.126016000	-5.412846000
H	-1.471824000	0.109233000	-4.732044000
H	2.253305000	5.402356000	-0.207167000
H	-0.968626000	6.371580000	-2.879432000
H	0.964929000	6.372085000	2.879385000
H	-2.256519000	5.401202000	0.207158000
C	-2.621052000	-0.807338000	3.762206000
C	-3.766398000	-0.480641000	2.978810000
C	-4.964253000	-0.972663000	-2.249416000
C	-5.567327000	-1.964055000	-3.082944000
C	-4.089782000	-2.777835000	-0.793470000
C	-4.167946000	-1.370537000	-1.146481000

C	-3.530366000	-3.281966000	0.445096000
C	-3.437983000	-2.535598000	1.689699000
C	-1.806225000	-4.194071000	2.485723000
C	-2.438461000	-2.922473000	2.609998000
C	-2.156705000	-5.041172000	1.483753000
C	-3.017642000	-4.599823000	0.435712000
C	-4.529002000	-3.709270000	-1.752111000
C	-5.299483000	-3.277329000	-2.878148000
C	-4.400443000	0.793746000	3.136774000
H	-1.745500000	-6.044758000	1.431125000
C	-4.168912000	-5.082906000	-1.634103000
C	-3.353823000	-5.490828000	-0.625775000
H	-2.984808000	-6.511177000	-0.584125000
C	-2.045039000	-2.033147000	3.652292000
C	-4.272323000	-1.407342000	2.048170000
C	-5.523994000	1.107030000	2.445936000
C	-6.188077000	0.111073000	1.661074000
C	-5.606094000	-1.174487000	1.521253000
H	-6.181387000	-1.643869000	-3.919290000
H	-5.677141000	-4.032330000	-3.561422000
H	-4.495195000	-5.777053000	-2.402785000
H	-1.072545000	-4.482540000	3.231600000
H	-1.223784000	-2.325563000	4.300095000
H	-2.253020000	-0.081625000	4.480925000
H	-3.938197000	1.517114000	3.802010000
H	-5.973437000	2.091913000	2.531386000
C	-5.098930000	0.402226000	-2.558309000
C	-3.409049000	-0.376905000	-0.494961000
C	-4.422918000	1.358798000	-1.847295000
C	-3.536657000	0.962362000	-0.816111000

H	-5.737409000	0.688800000	-3.388761000
H	-4.528521000	2.411114000	-2.087965000
H	-2.683108000	-0.653378000	0.256078000
C	-2.092777000	2.756413000	0.469287000
C	-2.760469000	1.934434000	-0.116808000
C	2.620806000	-0.807074000	-3.762268000
C	3.766050000	-0.479303000	-2.979161000
C	4.964853000	-0.969500000	2.249714000
C	5.568772000	-1.960328000	3.083284000
C	4.091856000	-2.775484000	0.793802000
C	4.168953000	-1.368083000	1.146732000
C	3.532668000	-3.280214000	-0.444640000
C	3.439466000	-2.534146000	-1.689397000
C	1.808797000	-4.194045000	-2.484758000
C	2.440039000	-2.921990000	-2.609401000
C	2.160180000	-5.040711000	-1.482749000
C	3.020989000	-4.598490000	-0.434971000
C	4.531903000	-3.706470000	1.752526000
C	5.302078000	-3.273831000	2.878499000
C	4.399054000	0.795526000	-3.137683000
H	1.749828000	-6.044632000	-1.429886000
C	4.172936000	-5.080407000	1.634729000
C	3.358050000	-5.489098000	0.626555000
H	2.989880000	-6.509757000	0.585045000
C	2.045732000	-2.033271000	-3.651878000
C	4.272878000	-1.405347000	-2.048349000
C	5.522527000	1.109905000	-2.447210000
C	6.187500000	0.114734000	-1.662119000
C	5.606539000	-1.171237000	-1.521706000
H	6.182552000	-1.639589000	3.919623000

H	5.680368000	-4.028487000	3.561805000
H	4.499870000	-5.774198000	2.403458000
H	1.075186000	-4.483201000	-3.230437000
H	1.224565000	-2.326497000	-4.299423000
H	2.252067000	-0.081816000	-4.481080000
H	3.936126000	1.518293000	-3.803094000
H	5.971242000	2.095078000	-2.533134000
C	5.098309000	0.405500000	2.558618000
C	3.409310000	-0.375081000	0.495121000
C	4.421526000	1.361488000	1.847566000
C	3.535716000	0.964301000	0.816281000
H	5.736486000	0.692628000	3.389110000
H	4.526200000	2.413896000	2.088241000
H	2.683832000	-0.652257000	-0.256088000
C	2.090899000	2.757459000	-0.469312000
C	2.758916000	1.935796000	0.116856000
H	1.327438000	3.982781000	3.298363000
H	-1.329898000	3.982088000	-3.298416000
C	-0.001622000	8.338567000	1.541455000
H	1.026252000	7.997579000	1.375251000
H	-0.136316000	8.545003000	2.610152000
C	-0.296118000	9.577462000	0.709124000
H	0.096083000	10.447726000	1.244968000
H	1.378605000	9.702095000	-0.666358000
H	-1.384088000	9.701408000	0.666337000
H	-0.101953000	10.447633000	-1.245010000
C	0.290700000	9.577592000	-0.709140000
C	-0.003156000	8.338533000	-1.541452000
H	0.131355000	8.545065000	-2.610153000
H	-1.030826000	7.996962000	-1.375180000

C	6.394141000	-2.188595000	-0.932699000
C	7.448674000	0.380566000	-1.080312000
C	8.155652000	-0.610713000	-0.441907000
C	7.634197000	-1.917406000	-0.401920000
H	6.020805000	-3.204483000	-0.908889000
H	8.214492000	-2.718823000	0.043837000
H	9.125092000	-0.397586000	-0.003300000
H	7.861930000	1.381317000	-1.169963000
C	-6.392779000	-2.192727000	0.932546000
C	-7.449344000	0.375675000	1.078924000
C	-8.155432000	-0.616429000	0.440809000
C	-7.632960000	-1.922731000	0.401443000
H	-6.018594000	-3.208317000	0.909204000
H	-8.212553000	-2.724775000	-0.044099000
H	-9.124961000	-0.404233000	0.001946000
H	-7.863414000	1.376134000	1.168083000

E= -3917.3117597 Hartrees.

6. Molecular dynamics simulations.

The previously described DFT calculations have explained the remarkable chiroptical properties of compound **1**, as well as elucidating the observed differences between g_{abs} and g_{lum} values. However, the theoretically predicted g_{abs} and g_{lum} values are significantly higher than those obtained experimentally, regardless of the functional employed.

One possible explanation for these discrepancies lies in the molecular flexibility, particularly due to the presence of alkynes. This flexibility, combined with solvent interactions, could cause the molecule to fluctuate between various semi-open conformations, in which the angle between the dipole moments deviates further from **1**, thereby diminishing the chiroptical response. The conformational equilibrium unequivocally demonstrates that the most stable conformers are those that are full-folded. However, this representation of the system may be too static to study the full behavior in depth.

To gain deeper insight into the influence of the solvent, molecular dynamics simulations were performed using GROMACS[16] and the following protocol: The three-dimensional structure of **1** was generated using Avogadro software[17] and the topology file of each molecule coordinate was generated using the online server SwissParams[18] with the CHARMM force field.[19] For every solvent studied, two distinct systems were built. The first system was composed of 26 molecules in a 12x12x12 nm cubic box. These systems, higher in concentration than the experiments, were designed to study the feasibility of developing unexpected intermolecular

interactions, the presence of aromatic stacking between conformers, and the role of entropic forces on the whole system. Once this preliminary study was done, the second system consisted of 52 molecules in a 35x35x35 nm box, which was a compromise solution between the employed experimental concentration ($2.5\cdot10^{-5}$ M) and a system big enough to contain a number of structures over 50 with wall-clock time simulations of 5 to 7 days. These systems were developed to account for the population distribution of the conformers, as a tool to directly extract structures and provide a partial description of the statistical distribution in a sample size greater than 30.

MD simulations were carried out using GROMACS 2023 software, with the inbuilt CHARMM27 force field. This force field is not only versatile but also extensively utilized in diverse supramolecular simulations.[20] After preparing these simulation boxes, energy minimization was performed using the steepest descent algorithm. To ensure a strictly minimized system, especially considering the systems where the solvent was not previously optimized, the cut-off value for the maximum tolerable force was kept at < 800 kJ/mol, and the number of steps was increased up to 500,000. Afterwards, the energy-minimized structures were equilibrated at both constant volume and pressure. The LINCS algorithm was utilized to constrain only the bond lengths involving hydrogen. To achieve the desired temperature of 298 K, the modified Berendsen thermostat known as V-rescale was employed for 100 ps. To achieve a pressure of 1 bar, the Berendsen barostat algorithm was utilized for 500 ps. Compressibility was adjusted according to $1.20\cdot10^{-4}$ and the Tau parameter was increased up to 5 to ensure slow and smooth coupling. Particle Mesh Ewald model was considered to account for long-range interactions, with 1nm cut-off for short-range electrostatic and van der Waals interactions. After the equilibration phases, the simulations were carried out for 5 ns for the different systems

After completing the MD simulation, the molecular structures were extracted and further analyzed using TD-DFT calculations at the ωB97X-D/6-31G(d,p) level of theory. Key parameters of the $S_1 \rightarrow S_0$ transition were tabulated, and the dissymmetry factor *g* was calculated. For the (*P*,*P*,*P*) compound, the weighted g_{lum}-factor was found to be $1.4\cdot10^{-2}$ (Table S7), in agreement with experimental values, highlighting the importance of accounting for molecular dynamics in flexible systems. In contrast, for the (*P*,*P*,*M*) molecule, the weighted g_{lum}-factor was significantly lower at $3.8\cdot10^{-3}$, consistent with the lack of optical activity observed for this compound, attributed to the interconversion of the central *o*-OPE unit between the *P* and *M* configurations (Table S8). The output files can be found in the ZENODO repository by consulting the following link: https://doi.org/10.5281/zenodo.15535155.

Table S7: Relevant transition parameters from TD-DFT for (*P*,*P*,*P*)-**1** MD-Derived Structures.

Molecule	$\mu\cdot10^{18}$/esu cm	$m\cdot10^{20}$/erg G-1	cos θ	g_{lum}
**1**	0.83	0.99	0.66	3.15E-02
**2**	0.97	1.12	0.79	3.61E-02
**3**	1.38	1.46	0.24	1.03E-02
**4**	0.85	1.17	0.25	1.35E-02
**5**	0.66	1.29	0.70	5.44E-02
**6**	1.19	1.35	0.56	2.54E-02
**7**	1.41	1.52	0.40	1.71E-02
**8**	3.01	1.53	0.54	1.10E-02
**9**	0.59	2.66	-0.32	-5.65E-02
**10**	2.22	1.64	0.47	1.40E-02
**11**	1.39	1.39	0.26	1.04E-02
**12**	1.11	1.65	0.15	8.92E-03
**13**	1.47	1.45	0.59	2.32E-02
**14**	1.75	1.50	0.62	2.13E-02
**15**	1.83	1.77	0.38	1.47E-02

**16**	4.05	4.23	0.46	1.94E-02
**17**	1.28	1.36	0.25	1.08E-02
**18**	0.53	0.83	0.62	3.85E-02
**19**	1.61	1.88	0.06	2.58E-03
**20**	1.34	1.68	0.42	2.12E-02
**21**	3.26	2.33	0.53	1.50E-02
**22**	2.86	2.54	0.63	2.23E-02
**23**	0.90	0.92	0.33	1.37E-02
**24**	1.12	1.10	0.33	1.30E-02
**25**	1.50	1.77	0.64	3.03E-02
**26**	1.57	0.92	0.35	8.26E-03
**27**	3.10	2.21	0.27	7.78E-03
**28**	1.64	2.35	0.17	9.84E-03
**29**	1.56	2.15	0.07	3.78E-03
**30**	1.59	1.32	0.50	1.66E-02
**31**	1.57	1.39	0.26	9.07E-03
**32**	1.11	1.17	0.23	9.78E-03
**33**	0.88	0.87	0.33	1.29E-02
**34**	1.36	1.35	0.23	9.27E-03
**35**	1.04	1.38	0.41	2.19E-02
**36**	2.76	1.37	0.00	-4.04E-05
**37**	2.90	2.99	0.40	1.64E-02
**38**	1.36	1.23	0.39	1.42E-02
**39**	0.15	0.23	0.14	9.17E-03
**40**	1.77	2.02	0.62	2.84E-02
**41**	1.17	1.39	0.18	8.42E-03
**42**	1.28	1.10	0.23	7.86E-03
**43**	2.17	2.02	0.70	2.59E-02
**44**	3.28	2.56	0.28	8.80E-03
**45**	1.13	1.50	0.14	7.17E-03
**46**	2.38	2.15	0.31	1.11E-02
**47**	3.63	2.42	0.66	1.75E-02
**48**	0.72	0.66	-0.11	-4.08E-03
**49**	1.05	0.99	0.70	2.67E-02
**50**	2.54	1.89	0.19	5.73E-03
**51**	0.53	0.38	0.11	3.30E-03
**52**	2.31	2.04	0.28	9.92E-03
			**Average g_{lum}**	**1.40E-02**

Table S8: Relevant transition parameters from TD-DFT for (*P*,*P*,*M*)-**1** MD-Derived Structures.

Molecule	$\mu \cdot 10^{18}$/esu cm	$m \cdot 10^{20}$/erg G-1	cos θ	g_{lum}
**1**	1.42	1.03	0.29	8.31E-03
**2**	1.82	2.55	0.29	1.65E-02
**3**	1.78	1.96	0.22	9.51E-03
**4**	0.43	0.40	0.03	9.98E-04
**5**	0.72	0.56	0.16	5.12E-03
**6**	0.84	1.19	-0.19	-1.07E-02
**7**	0.91	0.71	0.21	6.50E-03
**8**	1.41	1.83	0.05	2.67E-03
**9**	0.48	0.25	0.31	6.56E-03
**10**	1.95	1.91	-0.41	-1.62E-02
**11**	0.20	0.21	-0.53	-2.24E-02
**12**	0.33	0.27	0.34	1.13E-02
**13**	0.94	1.20	0.72	3.68E-02
**14**	0.87	1.11	-0.14	-6.95E-03
**15**	0.16	0.12	0.62	1.80E-02

**16**	1.07	1.34	0.67	3.36E-02
**17**	0.90	1.28	-0.05	-2.91E-03
**18**	0.60	0.75	0.53	2.64E-02
**19**	1.40	1.13	-0.10	-3.15E-03
**20**	1.15	1.34	-0.12	-5.41E-03
**21**	0.60	0.34	-0.29	-6.62E-03
**22**	1.15	1.18	-0.48	-1.96E-02
**23**	1.05	1.31	0.18	8.84E-03
**24**	0.71	0.59	0.40	1.33E-02
**25**	1.28	0.67	-0.16	-3.39E-03
**26**	1.52	1.40	0.75	2.78E-02
**27**	0.91	0.62	0.57	1.55E-02
**28**	0.77	0.53	-0.10	-2.65E-03
**29**	1.93	1.96	0.60	2.43E-02
**30**	1.69	1.89	0.20	9.04E-03
**31**	1.84	1.55	0.20	6.89E-03
**32**	1.06	0.30	0.39	4.44E-03
**33**	1.03	1.35	0.00	1.70E-04
**34**	0.61	0.68	0.32	1.42E-02
**35**	0.72	0.28	-0.53	-8.25E-03
**36**	1.52	1.37	0.22	8.04E-03
**37**	1.32	1.20	-0.17	-6.16E-03
**38**	1.45	1.63	0.24	1.08E-02
**39**	1.00	1.06	-0.51	-2.15E-02
**40**	1.67	1.43	0.14	4.96E-03
**41**	0.44	0.59	-0.04	-2.07E-03
**42**	0.55	0.10	0.75	5.33E-03
**43**	1.41	1.47	-0.56	-2.35E-02
**44**	0.30	0.43	0.43	2.44E-02
**45**	0.60	0.58	0.64	2.51E-02
**46**	0.14	0.05	0.10	1.55E-03
**47**	0.94	0.93	-0.72	-2.84E-02
**48**	0.90	1.04	-0.47	-2.18E-02
**49**	1.09	1.06	-0.13	-5.11E-03
**50**	0.80	0.67	0.04	1.24E-03
**51**	0.84	0.80	0.50	1.90E-02
**52**	1.39	1.16	-0.43	-1.45E-02
			**Average g_{lum}**	**3.38E-03**

6.1. Input files for MD simulations

Minim.mdp

```
; minim.mdp - used as input into grompp to generate em.tpr
; Parameters describing what to do, when to stop and what to save
integrator  = steep         ; Algorithm (steep = steepest descent
minimization)
emtol       = 800.0        ; Stop minimization when the maximum force <
1000.0 kJ/mol/nm
emstep      = 0.005          ; Minimization step size
nsteps      = 500000         ; Maximum number of (minimization) steps to
perform

; Parameters describing how to find the neighbors of each atom and how to
calculate the interactions
```

```
nstlist         = 1         ; Frequency to update the neighbor list and long
range forces
cutoff-scheme   = Verlet    ; Buffered neighbor searching
ns_type         = grid      ; Method to determine neighbor list (simple,
grid)
coulombtype     = PME       ; Treatment of long range electrostatic
interactions
rcoulomb        = 1.0       ; Short-range electrostatic cut-off
rvdw            = 1.0       ; Short-range Van der Waals cut-off
pbc             = xyz       ; Periodic Boundary Conditions in all 3
dimensions
```

nvt.md

```
title                   = T_293
; Run parameters
integrator              = md        ; leap-frog integrator
nsteps                  = 100000     ; 2 * 100000 = 200 ps
dt                      = 0.0016     ; 2 fs
; Output control
nstxout                 = 500       ; save coordinates every 1.0 ps
nstvout                 = 500       ; save velocities every 1.0 ps
nstenergy               = 500       ; save energies every 1.0 ps
nstlog                  = 500       ; update log file every 1.0 ps
; Bond parameters
continuation            = no        ; first dynamics run
constraint_algorithm    = lincs     ; holonomic constraints
constraints             = h-bonds   ; bonds involving H are constrained
lincs_iter              = 2         ; accuracy of LINCS
lincs_order             = 6         ; also related to accuracy
; Nonbonded settings
cutoff-scheme           = Verlet    ; Buffered neighbor searching
ns_type                 = grid      ; search neighboring grid cells
nstlist                 = 10        ; 20 fs, largely irrelevant with Verlet
rcoulomb                = 1.0       ; short-range electrostatic cutoff (in
nm)
rvdw                    = 1.0       ; short-range van der Waals cutoff (in
nm)
DispCorr                = EnerPres  ; account for cut-off vdW scheme
; Electrostatics
coulombtype             = PME       ; Particle Mesh Ewald for long-range
electrostatics
pme_order               = 4         ; cubic interpolation
fourierspacing          = 0.16      ; grid spacing for FFT
; Temperature coupling is on
tcoupl                  = V-rescale             ; modified Berendsen
thermostat
tc-grps                 = System ; two coupling groups - more accurate
tau_t                   = 0.1    ; time constant, in ps
ref_t                   = 298    ; reference temperature, one for each group,
in K
; Pressure coupling is off
pcoupl                  = no        ; no pressure coupling in NVT
; Periodic boundary conditions
pbc                     = xyz       ; 3-D PBC
; Velocity generation
gen_vel                 = yes       ; assign velocities from Maxwell
distribution
gen_temp                = 298       ; temperature for Maxwell distribution
```

```
gen_seed                = -1        ; generate a random seed
```

npt.md

```
title                   = T_298
; Run parameters
integrator              = md        ; leap-frog integrator
nsteps                  = 100000     ; 2 * 100000 = 200 ps
dt                      = 0.0016     ; 2 fs
; Output control
nstxout                 = 500       ; save coordinates every 1.0 ps
nstvout                 = 500       ; save velocities every 1.0 ps
nstenergy               = 500       ; save energies every 1.0 ps
nstlog                  = 500       ; update log file every 1.0 ps
; Bond parameters
continuation            = yes        ; first dynamics run
constraint_algorithm    = lincs     ; holonomic constraints
constraints             = h-bonds   ; bonds involving H are constrained
lincs_iter              = 2         ; accuracy of LINCS
lincs_order             = 6         ; also related to accuracy
; Nonbonded settings
cutoff-scheme           = Verlet    ; Buffered neighbor searching
ns_type                 = grid      ; search neighboring grid cells
nstlist                 = 10        ; 20 fs, largely irrelevant with Verlet
scheme
rcoulomb                = 1.0       ; short-range electrostatic cutoff (in
nm)
rvdw                    = 1.0       ; short-range van der Waals cutoff (in
nm)
DispCorr                = EnerPres  ; account for cut-off vdW scheme
; Electrostatics
coulombtype             = PME       ; Particle Mesh Ewald for long-range
electrostatics
pme_order               = 4         ; cubic interpolation
fourierspacing          = 0.16      ; grid spacing for FFT
; Temperature coupling is on
tcoupl                  = V-rescale             ; modified Berendsen
thermostat
tc-grps                 = System ; two coupling groups - more accurate
tau_t                   = 0.1    ; time constant, in ps
ref_t                   = 298    ; reference temperature, one for each group,
in K
; Pressure coupling is on
pcoupl                  = Berendsen     ; Pressure coupling on in NPT
pcoupltype              = isotropic             ; uniform scaling of box
vectors
tau_p                   = 5.0                   ; time constant, in ps
ref_p                   = 1.0                   ; reference pressure, in bar
compressibility         = 1.2e-4                ; isothermal compressibility
of water, bar^-1
refcoord_scaling        = com
; Periodic boundary conditions
pbc                     = xyz       ; 3-D PBC
; Velocity generation
gen_vel                 = no        ; Velocity generation is off
```

md.mdp

```
title                    = MD_298
; Run parameters
;define = -DPOSRES_SLAB
;refcoord_scaling = com
;define = -DFLEXIBLE
integrator               = md        ; leap-frog integrator
nsteps                   = 5000000000     ; 0.002 * 100000 = 500 ps
dt                       = 0.0018     ; 2 fs
; Output control
nstxout                  = 0         ; suppress bulky .trr file by specifying
nstvout                  = 0         ; 0 for output frequency of nstxout,
nstfout                  = 0         ; nstvout, and nstfout
nstenergy                = 500000      ; save energies every 10.0 ps
nstlog                   = 500000      ; update log file every 10.0 ps
nstxout-compressed       = 500000      ; save compressed coordinates every
10.0 ps
compressed-x-grps        = System    ; save the whole system
; Bond parameters
continuation             = yes       ; Restarting after NPT
constraint_algorithm     = lincs     ; holonomic constraints
constraints              = h-bonds   ; bonds involving H are constrained
lincs_iter               = 1         ; accuracy of LINCS
lincs_order              = 4         ; also related to accuracy
; Neighborsearching
cutoff-scheme            = Verlet    ; Buffered neighbor searching
ns_type                  = grid      ; search neighboring grid cells
nstlist                  = 10        ; 20 fs, largely irrelevant with Verlet
scheme
rcoulomb                 = 1.0       ; short-range electrostatic cutoff (in
nm)
rvdw                     = 1.0       ; short-range van der Waals cutoff (in
nm)
; Electrostatics
coulombtype              = PME       ; Particle Mesh Ewald for long-range
electrostatics
pme_order                = 4         ; cubic interpolation
fourierspacing           = 0.2      ; grid spacing for FFT
; Temperature coupling is on
tcoupl                   = V-rescale             ; modified Berendsen
thermostat
tc-grps                  = System   ; two coupling groups - more accurate
tau_t                    = 0.1      ; time constant, in ps
ref_t                    = 298      ; reference temperature, one for each
group, in K
; Pressure coupling is on
pcoupl                   = Parrinello-Rahman     ; Pressure coupling on in NPT
pcoupltype               = isotropic             ; uniform scaling of box
vectors
tau_p                    = 2                   ; time constant, in ps
ref_p                    = 1.0                   ; reference pressure, in bar
compressibility          = 4.5e-5                ; isothermal compressibility
of water, bar^-1
; Periodic boundary conditions
pbc                      = xyz       ; 3-D PBC
; Dispersion correction
DispCorr                 = EnerPres  ; account for cut-off vdW scheme
; Velocity generation
gen_vel                  = no        ; Velocity generation is off
;freeze Pd
```

```
freezegrps = Pd
freezedim = Y Y Y
```

7. References.

(1) Ortuño, A. M.; Reiné, P.; Resa, S.; Álvarez De Cienfuegos, L.; Blanco, V.; Paredes, J. M.; Mota, A. J.; Mazzeo, G.; Abbate, S.; Ugalde, J. M.; Mujica, V.; Longhi, G.; Miguel, D.; Cuerva, J. M. Extended Enantiopure *Ortho* -Phenylene Ethylene (*o* -OPE)-Based Helical Systems as Scaffolds for Supramolecular Architectures: A Study of Chiroptical Response and Its Connection to the CISS Effect. *Org. Chem. Front.* **2021**, *8* (18), 5071–5086. https://doi.org/10.1039/D1QO00822F.

(2) Walczak, R. M.; Cowart, J. S.; Reynolds, J. R. Tethered PProDOTs: Conformationally Restricted 3,4-Propylenedioxythiophene Based Electroactive Polymers. *J. Mater. Chem.* **2007**, *17* (3), 254–260. https://doi.org/10.1039/B610232H.

(3) Belviso, S.; Marsico, G.; Franzini, R.; Villani, C.; Abbate, S.; Longhi, G. Inherently Chiral Helicene-Substituted Thioalkyl Porphyrazine Complexes: Synthesis and Electronic and Chiroptical Properties. *Dalton Trans.* **2022**, *51* (43), 16453–16464. https://doi.org/10.1039/D2DT02665A.

(4) Valeur, B.; Berberan-Santos, M. N. *Molecular Fluorescence: Principles and Applications*, 1st ed.; Wiley, 2012. https://doi.org/10.1002/9783527650002.

(5) *Principles of Fluorescence Spectroscopy*; Lakowicz, J. R., Ed.; Springer US: Boston, MA, 2006. https://doi.org/10.1007/978-0-387-46312-4.

(6) Arrico, L.; Di Bari, L.; Zinna, F. Quantifying the Overall Efficiency of Circularly Polarized Emitters. *Chem. A European J.* **2021**, *27* (9), 2920–2934. https://doi.org/10.1002/chem.202002791.

(7) Turner, A. J.; Hick, P. E. Inhibition of Aldehyde Reductase by Acidic Metabolites of the Biogenic Amines. *Biochem Pharmacol* **1975**, *24* (18), 1731–1733. https://doi.org/10.1016/0006-2952(75)90016-7.

(8) Pracht, P.; Bohle, F.; Grimme, S. Automated Exploration of the Low-Energy Chemical Space with Fast Quantum Chemical Methods. *Phys. Chem. Chem. Phys.* **2020**, *22* (14), 7169–7192. https://doi.org/10.1039/C9CP06869D.

(9) Stephens, P. J.; Devlin, F. J.; Chabalowski, C. F.; Frisch, M. J. Ab Initio Calculation of Vibrational Absorption and Circular Dichroism Spectra Using Density Functional Force Fields. *J. Phys. Chem.* **1994**, *98* (45), 11623–11627. https://doi.org/10.1021/j100096a001.

(10) Zhao, Y.; Truhlar, D. G. The M06 Suite of Density Functionals for Main Group Thermochemistry, Thermochemical Kinetics, Noncovalent Interactions, Excited States, and Transition Elements: Two New Functionals and Systematic Testing of Four M06-Class Functionals and 12 Other Functionals. *Theor. Chem. Acc.* **2008**, *120* (1–3), 215–241. https://doi.org/10.1007/s00214-007-0310-x.

(11) Chai, J.-D.; Head-Gordon, M. Long-Range Corrected Hybrid Density Functionals with Damped Atom–Atom Dispersion Corrections. *Phys. Chem. Chem. Phys.* **2008**, *10* (44), 6615. https://doi.org/10.1039/b810189b.

(12) Yanai, T.; Tew, D. P.; Handy, N. C. A New Hybrid Exchange–Correlation Functional Using the Coulomb-Attenuating Method (CAM-B3LYP). *Chem. Phys. Lett.* **2004**, *393* (1–3), 51–57. https://doi.org/10.1016/j.cplett.2004.06.011.

(13) Hehre, W. J.; Ditchfield, R.; Pople, J. A. Self—Consistent Molecular Orbital Methods. XII. Further Extensions of Gaussian—Type Basis Sets for Use in Molecular Orbital Studies of Organic Molecules. *J. Chem. Phys.* **1972**, *56* (5), 2257–2261. https://doi.org/10.1063/1.1677527.

(14) Lu, T.; Chen, F. Multiwfn: A Multifunctional Wavefunction Analyzer. *J. Comput. Chem.* **2012**, *33* (5), 580–592. https://doi.org/10.1002/jcc.22885.

(15) Uceda, R. G.; Cruz, C. M.; Míguez-Lago, S.; De Cienfuegos, L. Á.; Longhi, G.; Pelta, D. A.; Novoa, P.; Mota, A. J.; Cuerva, J. M.; Miguel, D. Can Magnetic Dipole Transition Moment Be Engineered? *Angew Chem Int Ed* **2024**, *63* (4), e202316696. https://doi.org/10.1002/anie.202316696.

(16) Abraham, M. J.; Murtola, T.; Schulz, R.; Páll, S.; Smith, J. C.; Hess, B.; Lindahl, E. GROMACS: High Performance Molecular Simulations through Multi-Level Parallelism from Laptops to Supercomputers. *SoftwareX* **2015**, *1–2*, 19–25. https://doi.org/10.1016/j.softx.2015.06.001.

(17) Hanwell, M. D.; Curtis, D. E.; Lonie, D. C.; Vandermeersch, T.; Zurek, E.; Hutchison, G. R. Avogadro: An Advanced Semantic Chemical Editor, Visualization, and Analysis Platform. *J. Cheminform* **2012**, *4* (1), 17. https://doi.org/10.1186/1758-2946-4-17.
(18) Zoete, V.; Cuendet, M. A.; Grosdidier, A.; Michielin, O. SwissParam: A Fast Force Field Generation Tool for Small Organic Molecules. *J. Comput. Chem.* **2011**, *32* (11), 2359–2368. https://doi.org/10.1002/jcc.21816.
(19) Jo, S.; Kim, T.; Iyer, V. G.; Im, W. CHARMM-GUI: A Web-based Graphical User Interface for CHARMM. *J. Comput. Chem.* **2008**, *29* (11), 1859–1865. https://doi.org/10.1002/jcc.20945.
(20) Frederix, P. W. J. M.; Patmanidis, I.; Marrink, S. J. Molecular Simulations of Self-Assembling Bio-Inspired Supramolecular Systems and Their Connection to Experiments. *Chem. Soc. Rev.* **2018**, *47* (10), 3470–3489. https://doi.org/10.1039/C8CS00040A.
